# Supplementary material for: Protocol for the challenge non-typhoidal Salmonella (CHANTS) study: a first-in-human, in-patient, double-blind, randomised, safety and dose-escalation controlled human infection model in the UK
Source: BMJ Open. 2024 Jan 10;14(1):e076477. doi: 10.1136/bmjopen-2023-076477 (PMC10806722; doi:10.1136/bmjopen-2023-076477)

Supplementary Materials

Table 1: Exploratory Objectives

**Exploratory**	1	To investigate how the human microbiota interacts with oral challenge of *S.* Typhimurium 4/74 or D23580 strains	Metagenomic analysis of stool and/or saliva to measure constituent microbiological flora (and their metabolome/transcriptome) at baseline and post-challenge time points
	2	To investigate *Salmonella* Typhimurium virulence factors and gene expression	Exploratory analysis of stool samples at several time points following challenge to assess bacterial transcriptome by RNA-seq
	3	To describe and compare additional host immune responses following oral challenge of *S.* Typhimurium 4/74 or D23580 strains.	Analysis of B-cell repertoire, plasma cytokine profile and kinetics.
	4	To evaluate environmental contamination with *Salmonella enterica* in the near patient environment	The pattern and proportion of environmental samples testing positive for *Salmonella* Typhimurium by culture or molecular methods.
	5	To evaluate host-pathogen interactions following *Salmonella* infection including the presence and mechanisms of *Salmonella* persister infected macrophages.	Exploratory analysis of infected immune cells from blood including, but not limited to, single-cell RNA-seq.

Table 2: Eligibility criteria for participation

**Inclusion Criteria**
Agree to give informed consent for participation in the study.
Aged between 18 and 50 years inclusive at time of challenge.
In good health as determined by medical history, physical examination, and clinical judgment of the study team.
Agree (in the study team's opinion) to comply with all study requirements, including capacity to adhere to good personal hygiene and infection control precautions.
Agree to allow his or her General Practitioner (and/or Consultant if appropriate), to be notified of participation in the study.
Agree to allow study staff to contact his or her GP to access the participant's medical history and vaccination records.
Agree to allow UKHSA and the local health protection unit to be informed of their participation in the study.
Agree to give his or her close contacts written information informing them of the participant's involvement in the study and offer them voluntary screening for Salmonella carriage.
Agree to a period of inpatient quarantine whilst symptomatic (In exceptional circumstances, the duration of inpatient quarantine may be extended.)
Agree to have 24-hour contact with study staff during the four weeks post challenge and can ensure that they are contactable by mobile phone for the duration of the study period until antibiotic completion.
Agree to allow the study team to hold the name and 24-hour contact number of a close friend, relative or housemate who will be kept informed of the study participant's whereabouts for the duration of the challenge period after quarantine (from the time of challenge until completion of antibiotic course). This person will be contacted if study staff are unable to contact the participant after discharge from inpatient quarantine.
Have internet access to allow completion of the e-diary and real-time safety monitoring.
Agree to avoid antipyretic/anti-inflammatory treatment from the time of challenge (Day 0) until advised by a study doctor or until 14 days after challenge.
Agree to refrain from donating blood for the duration of the study.
Agree to provide their National Insurance/Passport number for the purposes of Trial over-volunteering prevention system (TOPS) registration and for payment of reimbursement expenses.
Participants must have received at least two doses of a SARS-CoV-2 vaccines that has been approved for use by the MHRA (or other national regulatory authority) four weeks prior to enrolment.
Proficient in English at a level sufficient to understand, retain, weigh up and communicate the study details as outlined in the participant information sheet, in the opinion of the study investigator.
**Exclusion Criteria**
History of microbiologically confirmed *Salmonella* infection[1].
History of receipt of oral Ty21a typhoid vaccination
History of **significant[2]** organ-specific and/or systemic disease that could interfere with trial conduct or completion. Including, for example, but not restricted to:<br>- Cardiovascular disease (ischaemic heart disease, valvular heart disease, vascular disease including aneurysmal disease or endovascular prostheses, prolonged corrected QT interval (>450 milliseconds) on ECG screening).

- Respiratory disease[3]<br>- Haematological disease including sickle cell disease and sickle cell trait.<br>- Endocrine disorders, including specifically diabetes mellitus.<br>- Renal or bladder disease, including history of renal calculi.<br>- Biliary tract disease, including specifically colic, asymptomatic gallstones, cholecystectomy.<br>- Gastro-intestinal disease including specifically: a current requirement for antacids, H_2-receptor antagonists, proton pump inhibitors or laxatives, Inflammatory bowel disease, confirmed diagnosis of irritable bowel syndrome as defined by the Rome IV criteria,<br>- Neurological disease<br>- Metabolic disease<br>- Autoimmune disease<br>- Psychiatric illness requiring hospitalisation.<br>- Known or suspected drug and/or alcohol misuse disorder<br>- Chronic/Active Infectious disease including active tuberculosis.<br>- Severe infection requiring hospitalisation for intravenous antibiotics within the last 10 years. Exceptions to this would include a short course of intravenous antibiotics for appendicitis, biliary sepsis, diverticulitis, and cellulitis.<br>- History of recent malaria infection in the past 12 months from screening<br>- History of joint replacement<br>- History of any orthopaedic/osseous implanted prosthesis<br>- Presence of any other internal implanted device e.g., permanent pacemaker.
Have any known or suspected impairment of immune function (as defined in the green book[140]), alteration of immune function, or prior immune exposure that may alter immune function to *Salmonella* infection resulting from, for example:<br>- Congenital or acquired immunodeficiency, including IgA deficiency.<br>- Human Immunodeficiency Virus infection or symptoms/signs suggestive of an HIV-associated condition<br>- Evidence of severe primary immunodeficiency, for example, severe combined immunodeficiency, Wiskott-Aldrich syndrome, and other combined immunodeficiency syndromes.<br>- Currently being treated for malignant disease with immunosuppressive chemotherapy or radiotherapy, or who have received such treatment within at least the last six months.<br>- Individuals who have received a solid organ transplant and are currently on immunosuppressive treatment.<br>- Individuals who have received a bone marrow transplant, until within 12 months of finishing all immunosuppressive treatment,<br>- Individuals receiving systemic high dose steroids[4] until at least three months after treatment has stopped.<br>- Individuals receiving other types of immunosuppressive[5] drugs (alone or in combination with lower doses of steroids) until at least six months after terminating such treatment.<br>- Receipt of immunoglobulin or any blood product transfusion within 3 months of study start.<br>- History of cancer (except squamous cell or basal cell carcinoma of the skin and cervical carcinoma in situ).
Moderate or severe depression or anxiety as classified by the Hospital Anxiety and Depression Score at screening or challenge that is deemed clinically significant by the study doctors[6].
Weight less than 50kg[7]
Anyone taking long-term medication (e.g., analgesia, anti-inflammatories, or antibiotics) that may affect symptom reporting or interpretation of the study results.
Contraindication to cephalosporin, fluroquinolone, or macrolide antibiotics.

Female participants who are pregnant, lactating or who are unwilling to ensure that they or their partner use effective contraception[8] 30 days prior to challenge and continue to do so until two negative stool samples, a minimum of 3 weeks after completion of antibiotic treatment, have been obtained.
Full-time, part-time, or voluntary occupations involving:<br>- Clinical healthcare work[9]<br>- Social work with direct contact with young children (defined as those attending pre-school groups or nursery or aged under 2 years), or other clinically vulnerable children, adolescents.<br>- Clinical or social work with direct contact with highly susceptible patients or persons in whom *Salmonella* infection would have particularly serious consequences (unless willing to avoid work until demonstrated not to be infected with *Salmonella* in accordance with guidance from Public Health England and willing to allow study staff to inform their employer).
Full time, part time or voluntary occupations involving:<br>- Commercial food handling (involving preparing or serving unwrapped foods not subjected to further heating)
Close household contact with:<br>- Children aged less than 2 years<br>- Any individual who is immunocompromised.<br>- Pregnant women<br>- Household contacts aged over 70 years
Scheduled elective surgery or other procedures requiring general anaesthesia during the study period.
Participants who have participated in another research study involving an investigational product that might affect risk of *Salmonella* infection or compromise the integrity of the study within the 30 days prior to enrolment (e.g. significant volumes of blood already taken in previous study)[10].
Detection of any abnormal results from screening investigations, unless deemed not clinically significant.
Screening blood test positive for HLA-B*27 Antigen
Any other social, psychological or health issues which, in the opinion of the study staff, may:<br>- Put the participant or their contacts at risk because of participation in the study.<br>- Adversely affect the interpretation of the primary endpoint data.<br>- Impair the participant's ability to participate in the study.
Having previously received any experimental *Salmonella* vaccine as part of a clinical trial
Have participated in previous *Salmonella* Typhi or Paratyphi challenge studies (with ingestion of challenge agent).
Any employee of the sponsor or research site personnel directly affiliated with this study or their immediate family members.
Inability to comply with any of the study requirements (at the discretion of the study staff).

[1] Including enteric fever (*Salmonella* Typhi and/or *Salmonella* Paratyphi infection)

[2] Individuals with specific medical co-morbidities (e.g., childhood asthma) will be considered for enrolment after review with the principal investigator and clinical oversight group, following discussion with their GP and/or consultant as appropriate. The rationale for enrolment in these cases will be recorded in the CRF.

[3] Participants with well controlled asthma may be considered for enrolment at the discretion of the study investigators and following consultation with their general practitioner.

[4] Defined as those receiving at least 40mg of prednisolone per day for more than one week or equivalent.

[5] Azathioprine, cyclosporin, methotrexate, cyclophosphamide, leflunomide and other cytokine inhibitors.

[6] If elevated scores are due to temporary significant life events, the questionnaire may be repeated after resolution of the event with a view to inclusion if normal.
[7] Or a Body Mass Index (BMI) that, in the opinion of the study doctors, may adversely impair the interpretation of the study results or affect the safe performance of any study procedures.
[8] All forms of contraception are considered effective with the exception of barrier methods and natural family planning. Accepted methods include hormonal contraceptive pills, intrauterine contraceptive devices/systems, long-acting injectable hormonal contraception, contraceptive implants.
[9] This includes medical, dental, nursing or midwifery students (or other allied-healthcare professionals) with direct patient contact. Such students who are not undertaking patient-facing rotations until demonstrated not to be infected with *Salmonella* species may be considered for enrolment.
[10] As assessed by both participant questioning and registration with The Over Volunteering Prevention System (TOPS) database.

Table 3: Temporary Exclusion Criteria at time of bacterial challenge

**Temporary Exclusion Criteria at Challenge**
Positive lateral flow test for SARS-CoV-2
Significant acute or acute-on-chronic infection within the previous 7 days or have experienced fever (>37.5°C) or subjective febrile symptoms within the previous 3 days
History of any antibiotic therapy during the previous 5 days
Any systemic corticosteroid (or equivalent) treatment in the previous 14 days, or for more than seven consecutive days within the past 3 months
Therapy with antacids, proton pump inhibitors or H2-receptor antagonists within 24 hours prior to challenge
Detection of gastrointestinal pathogens (other than *Salmonella*) in stool culture/PCR collected at the pre-challenge assessment (Day-7) including *Shigella* spp., *Campylobacter* spp., *E. Coli* O157, *Giardia* spp and *Cryptosporidium* spp, until two subsequent samples are negative. Detection of *Salmonella* spp. in stool prior to challenge (Day – 7) will result in exclusion from the study
Detection of extended spectrum beta lactamase producing organisms (ESBLs) or Carbapenem resistant organisms (CRO) until two subsequent samples are negative.

Table 4: Clinical sample collection plan for patients undergoing primary challenge. Values correspond to volume of blood collected in millilitres.

	Pre-challenge		Challenge period																				Follow up			
			Inpatient quarantine								Outpatient assessments							Post diagnosis/treatment								
	Screening	Day -7	Day 0	Day 1	Day 2	Day 3	Day 4	Day 5	Day 6	Day 7	Day 8	Day 9	Day 10	Day 11	Day 12	Day 13	Day 14	SD	SD +24h	SD +48h	SD +72h	SD +96h	Day 28	Day 90	Day 180	Day 365
Stool sample		x	x	x	x	x	x	x	x	x	x	x	x	x	x	x	x	x	x	x	x	x	x	x	x	x
Blood culture BACTEC			10	10	10	10	10	10	10	10	10	10	10	10	10	10	10	10	10	10	10	10				
FBC	1	1	1		1		1		1		1		1		1		1	1	1	1	1	1	1	1	1	
U&E/CRP/LFT	2	2	2		2		2		2		2		2		2		2	2	2	2	2	2	2	2	2	
Additional screening investigations*	10																									
Bacterial quantification																		10								
Serum sample			10							10							10	10					10	10	10	10
Plasma Sample			3							3							3	3					3			
PBMC			25				25			25							25	25					25	25	25	25
Functional genomics			3	3	3	3				3								3					3	3		
Whole blood PCR			10	10	10	10	10	10	10	10																
DNA sample (including epigenetics)			x																				x		x	
Saliva sample			x							x							x						x		x	

*HIV/HBV/HV serology, HLA-B27, Haemoglobinopathy screen, Coeliac serology, HbA1c

Table 5: Simulation results comparing CRM with rule-based model.

Operating characteristics for CRM-based approach under 5 following scenarios:

Scenario 1 target dose at dose 1 with attack rate: 0.675, 0.8, 0.9, 0.95.
Scenario 2 target dose at dose 2 with attack rate: 0.5, 0.675, 0.8, 0.9.
Scenario 3 target dose at dose 4 with attack rate: 0.3, 0.4, 0.5, 0.675.
Scenario 4 target dose beyond dose 4 with attack rate: 0.2, 0.3, 0.4, 0.5.
Scenario 5 target dose under dose 1 with attack rate: 0.8, 0.9, 0.95, 0.975.

	**CRM-based***	**Rule-based**
**Scenario 1**		
Median sample size (IQR)	20 (20-25)	20 (20-25)
Probability of choosing the dose		
First dose too high	17.2%	16.4%
1 (67.5%)	69.1%	58.4%
2 (80%)	13.7%	8.6%
3 (90%)		
4 (95%)		
Fail to find a dose		16.6%
**Scenario 2**		
Median sample size (IQR)	30 (25-35)	30 (25-35)
Probability of choosing the dose		
First dose too high	0.7%	0.8%
1 (50%)	20.6%	17.3%
2 (67.5%)	64.9%	49.4%
3 (80%)	13.7%	3.9%
4 (90%)	0.1%	
Failed to find a dose	N/A	28.6%
**Scenario 3**		
Median sample size	35 (35-40)	30 (20-40)
Probability of choosing the dose		
1 (30%)	0.02%	0.6%
2 (40%)	2.2%	4.4%
3 (50%)	34.1%	14.1%
4 (67.5%)	59.1%	11.0%
Last dose too low	4.4%	0%
Fail to find a dose	N/A	69.9%
**Scenario 4**		
Median sample size	35 (35-40)	30 (25-35)
Probability of choosing the dose		
1 (20%)	0%	0%
2 (30%)	0.1%	0.8%
3 (40%)	5.3%	3.1%
4 (50%)	46.0%	10.7%
Last dose too low	48.6%	

Fail to find a dose	N/A	85.4%
**Scenario 5**		
Median sample size	20 (20-20)	20 (20-20)
Probability of choosing the dose		
First dose too high	64.7%	63.2%
1 (80%)	35.0%	32.3%
2 (90%)	0.3%	0.1%
3 (95%)	0%	0%
4 (97.5%)	0%	0%
Fail to find a dose	N/A	4.4%

*Parameters for the CRM-based design:

- Prior attack rate at the four doses: 0.6, 0.75, 0.85, 0.9;
- The attack rate is 67.5%;
- Starting at dose 1

Table 6 – ICMJE Data Availability Statement

**Which data in particular will be shared?**	**Are individual deidentified participant data (including data dictionaries) available?**	**Are additional, related documents available (e.g., study protocol, statistical analysis plan, etc.)?**	**When will the data become available and for how long?**	**By what access criteria will the data be shared (including with whom, for what types of analyses, and by what mechanism)?**
Individual participant data that underlie the results reported in the trial, after deidentification	Yes, pertaining to established endpoints of the trial	Study protocol	Following publication of the trial, ending 36 months from publication date	Investigators whose proposed use of data has been approved by an independent ethics review committee, identified for this purpose

Figure 1: Antibiotic Treatment Algorithm

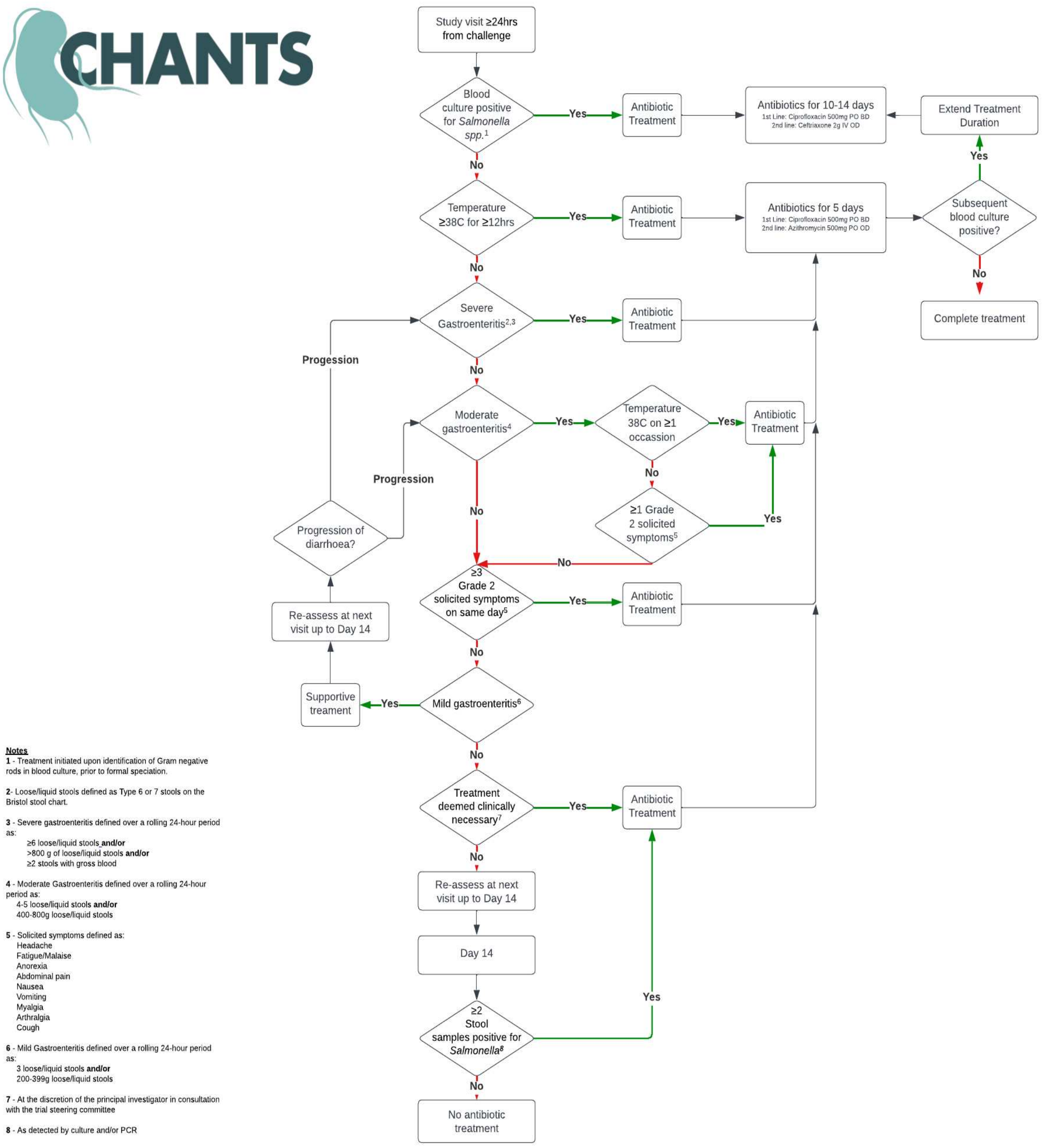

Notes

1 - Treatment initiated upon identification of Gram negative rods in blood culture, prior to formal speciation.

2- Loose/liquid stools defined as Type 6 or 7 stools on the Bristol stool chart.

3 - Severe gastroenteritis defined over a rolling 24-hour period as:
- ≥6 loose/liquid stools **and/or**
- >800 g of loose/liquid stools **and/or**
- ≥2 stools with gross blood

4 - Moderate Gastroenteritis defined over a rolling 24-hour period as:
- 4-5 loose/liquid stools **and/or**
- 400-800g loose/liquid stools

5 - Solicited symptoms defined as:
- Headache
- Fatigue/Malaise
- Anorexia
- Abdominal pain
- Nausea
- Vomiting
- Myalgia
- Arthralgia
- Cough

6 - Mild Gastroenteritis defined over a rolling 24-hour period as:
- 3 loose/liquid stools **and/or**
- 200-399g loose/liquid stools

7 - At the discretion of the principal investigator in consultation with the trial steering committee

8 - As detected by culture and/or PCR

Figure 2: Quarantine Discharge Algorithm

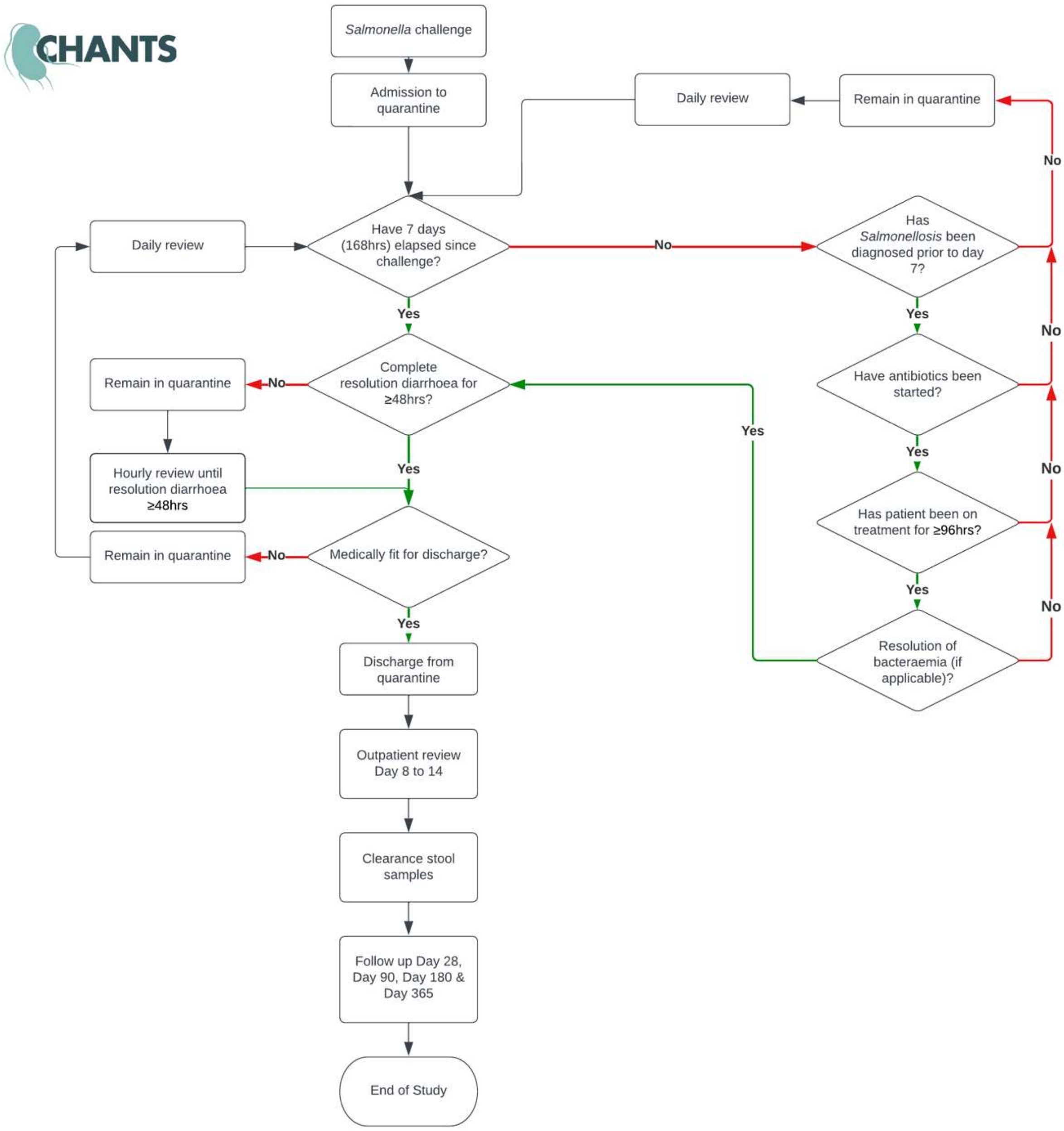

Supplement: Supplementary data [file bmjopen-2023-076477supp001.pdf]
