# Supplementary material for: Protocol for the challenge non-typhoidal Salmonella (CHANTS) study: a first-in-human, in-patient, double-blind, randomised, safety and dose-escalation controlled human infection model in the UK
Source: BMJ Open. 2024 Jan 10;14(1):e076477. doi: 10.1136/bmjopen-2023-076477 (PMC10806722; doi:10.1136/bmjopen-2023-076477)
Supplement: Author's manuscript [file bmjopen-2023-076477.draft_revisions.pdf]

BMJ Open is committed to open peer review. As part of this commitment we make the peer review history of every article we publish publicly available.

When an article is published we post the peer reviewers' comments and the authors' responses online. We also post the versions of the paper that were used during peer review. These are the versions that the peer review comments apply to.

The versions of the paper that follow are the versions that were submitted during the peer review process. They are not the versions of record or the final published versions. They should not be cited or distributed as the published version of this manuscript.

BMJ Open is an open access journal and the full, final, typeset and author-corrected version of record of the manuscript is available on our site with no access controls, subscription charges or pay-per-view fees (http://bmjopen.bmj.com).

If you have any questions on BMJ Open's open peer review process please email info.bmjopen@bmj.com

Protocol for the Challenge Nontyphoidal *Salmonella* (CHANTS) Study: a first-in-human, double-blind, randomised, safety and dose-escalation controlled human infection model

Journal:	*BMJ Open*
Manuscript ID	bmjopen-2023-076477
Article Type:	Protocol
Date Submitted by the Author:	08-Jun-2023

Complete List of Authors:	Smith, Christopher; Imperial College London, Department of Infectious Disease<br>Smith, Emma; Imperial College London, Department of Infectious Disease<br>Rydlova, Anna; Imperial College London, Department of Infectious Disease<br>Varro, Robert; Imperial College London, Department of Infectious Disease<br>Hinton, Jay; University of Liverpool, Institute of Infection, Veterinary and Ecological Sciences<br>Gordon, Melita; Kamuzu University of Health Sciences, Malawi Liverpool Wellcome Trust Clinical Research Programme; University of Liverpool, Institute of Infection, Veterinary and Ecological Sciences<br>Choy, Robert; PATH<br>Liu , Xinxue ; Oxford University, Oxford Vaccine Group, Department of Paediatrics ; NIHR Oxford Biomedical Research Centre<br>Pollard, Andrew; Oxford University, Oxford Vaccine Group, Department of Paediatrics; NIHR Oxford Biomedical Research Centre<br>Chiu, Christopher; Imperial College London, Department of Infectious Disease<br>Cooke, Graham; Imperial College London, Department of Infectious Disease<br>Gibani, Malick; Imperial College London, Department of Infectious Disease

Keywords:	INFECTIOUS DISEASES, Tropical medicine < INFECTIOUS DISEASES, Neglected Diseases, Microbiology < NATURAL SCIENCE DISCIPLINES, Immunology < NATURAL SCIENCE DISCIPLINES

SCHOLARONE™ Manuscripts

Protocol for the Challenge Nontyphoidal *Salmonella* (CHANTS) Study: a first-in-human, double-blind, randomised, safety and dose-escalation controlled human infection model.

Authors

Christopher Smith[1], Emma Smith[1], Anna Rydlova[1], Robert Varro[1], Jay C.D. Hinton[2], Melita A. Gordon[2,3], Robert K.M. Choy[4], Xinxue Liu[5,6], Andrew J. Pollard[5,6], Christopher Chiu[1], Graham S. Cooke[1], Malick M. Gibani[1]

[1]Department of Infectious Disease, Imperial College London, UK
[2]Institute of Infection, Veterinary and Ecological Sciences, University of Liverpool, UK
[3]Malawi-Liverpool-Wellcome Trust Clinical Research Programme, Malawi
[4]PATH, Seattle, USA
[5]Oxford Vaccine Group, Department of Paediatrics, University of Oxford, UK
[6]NIHR Oxford Biomedical Research Centre, Oxford, UK

Corresponding Author

Malick M. Gibani, Department of Infectious Disease, Imperial College London, St Mary's Campus, Norfolk Place, London, W2 1PG: m.gibani@imperial.ac.uk.

Keywords

Salmonella; *Salmonella* Typhimurium; Non-Typhoidal *Salmonella*; Human Challenge Model; Controlled Human Infection Model; Vaccines.

Protocol Version: 1.2

Study Sponsor

Imperial College London is the main research Sponsor for this study. For further information regarding the sponsorship conditions, please contact the Head of Regulatory Compliance at:

Research Governance and Integrity Team
Imperial College London and Imperial College Healthcare NHS Trust
Room 215, Level 2, Medical School Building
Norfolk Place
London, W2 1PG
Tel: 0207 594 1862

For peer review only - http://bmjopen.bmj.com/site/about/guidelines.xhtml

1 ABSTRACT

Introduction

Invasive nontyphoidal *Salmonella* (iNTS) serovars are a major cause of community-acquired bloodstream infections in sub-Saharan Africa (SSA). In this setting, *Salmonella enterica* serovar Typhimurium accounts for two-thirds of infections and is associated with an estimated case-fatality rate of 15-20%. Several iNTS vaccine candidates are in early-stage assessment which – if found effective – would provide a valuable public health tool to reduce iNTS disease burden. The CHANTS study aims to develop a first-in-human *Salmonella* Typhimurium controlled human infection model (CHIM) which can act as a platform for future vaccine evaluation, in addition to providing novel insights into iNTS disease pathogenesis.

Methods and Analysis

This double-blind, safety and dose-escalation study will randomise 40-80 healthy UK participants aged 18-50 to receive oral challenge with one of two strains of *S.* Typhimurium belonging to the ST19 (strain 4/74) or ST313 (strain D23580) lineages. 4/74 is a global strain often associated with diarrhoeal illness predominantly in high-income settings, whilst D23580 is an archetypal strain representing invasive disease-causing isolates found in SSA. The primary objective is to determine the minimum infectious dose (CFU) required for 60-75% of participants to develop clinical or microbiological features of systemic salmonellosis. Secondary endpoints are to describe and compare the clinical, microbiological, and immunological responses following challenge. Dose escalation or de-escalation will be undertaken by continual-reassessment methodology and limited within pre-specified safety thresholds. Exploratory objectives are to describe mechanisms of iNTS virulence, identify putative immune correlates of protection, and describe host-pathogen interactions in response to infection.

Ethics and Dissemination

Ethical approval has been obtained from the NHS Health Research Authority (London – Fulham Research Ethics Committee 21/PR/0051; IRAS Project ID 301659). The study findings will be disseminated in international peer-reviewed journals and presented at national/international stakeholder meetings. Study outcome summaries will be provided to both funders and participants.

Trial Registration

Clinicaltrials.gov (NCT05870150).

For peer review only - http://bmjopen.bmj.com/site/about/guidelines.xhtml

2 STRENGTHS AND LIMITATIONS

1. This first-in-human CHIM offers a novel platform for the detailed investigation of *S.* Typhimurium pathogenesis and host-pathogen interactions within a controlled infection model.
2. The strains used in this model (ST19 and ST313) have undergone extensive genomic, phenotypic, and transcriptomic characterisation, and are representative of currently circulating strains accountable for both diarrhoeal and invasive disease phenotypes.
3. A double-blind, randomised study design permits objective evaluation of differential clinical phenotypes induced by bacterial challenge. A prolonged follow-up phase also permits assessment of short- and long-term consequences of bacterial challenge, including microbial dynamics of infection, and clinical and immune responses.
4. The continual reassessment method for dose-finding, compared with rule-based design, improves trial efficiency, and increases the probability of finding the correct challenge dose. Establishment of a reliable and reproducible challenge dose will provide a platform for future iNTS CHIM studies, including homologous and heterologous re-challenge, and vaccine and therapeutic randomised trials.
5. The main limitations of this study are: i) the selection of clinically relevant study endpoints pertaining to future vaccine efficacy studies and ii) recruitment of a study population whose response to challenge is likely to differ from the populations most susceptible to iNTS, namely children and immunocompromised adults in sub-Saharan Africa.

For peer review only - http://bmjopen.bmj.com/site/about/guidelines.xhtml

3 INTRODUCTION

Nontyphoidal *Salmonella* (NTS) infections are most commonly associated with a self-limiting diarrhoeal illness (dNTS) [1]. In contrast, invasive nontyphoidal *Salmonella* disease (iNTS) typically presents as a non-specific febrile illness – often without diarrhoea – following invasion into sterile sites causing bloodstream infection (BSI), meningitis, or sepsis [2]. iNTS is particularly common in sub-Saharan Africa (SSA) where most recent estimates reported 535,000 cases, 77,500 deaths, and 4·26 million lost DALYs in 2017 [3]. Case fatality rates are estimated to range between 15-20%. The high case fatality reflects that invasive disease occurs almost exclusively in individuals with impaired immunity. Recognised host risk factors include malnutrition, malaria, and chronic anaemia in children, and HIV infection and malignancy in adults [2,4]. Effective treatment is also increasingly challenging; highlighted by the WHO's inclusion of *Salmonella enterica* as one of the priority pathogens posing the greatest risk to human health through expanding antimicrobial resistance (AMR) [5].

iNTS serovars isolated in community-acquired BSI exhibit geographic variation. In Africa, *Salmonella enterica* subspecies *enterica* serovars Typhimurium and Enteritidis (henceforth *S.* Typhimurium and *S.* Enteritidis) are responsible for >90% of all iNTS BSI, with *S.* Typhimurium accounting for two thirds of this burden [6–8]. Sequencing of isolates from across Africa has identified *S.* Typhimurium multilocus sequence type (ST) 313 as the major cause of invasive disease, which is distinct from sequence types 19 and 34 that are more frequently associated with diarrhoeal disease (dNTS) in high-income settings such as the UK. ST313 has been shown to have undergone sequential evolutionary changes that converge towards a genotype associated with invasive disease [9].

To date, there are no licensed iNTS vaccines available for use in humans. Development of effective vaccines may contribute to reduced case numbers, reduced antibiotic use, and prevention of antimicrobial resistance [10]. Several candidate vaccines are under evaluation, including in bivalent or multi-valent formulations which could target both typhoidal and nontyphoidal *Salmonella* serovars [11]. Vaccine development for iNTS is hampered – in part – by an incomplete understanding of mechanisms and determinants of immunity during natural infection. Investigation into these mechanisms could support iNTS vaccine development programmes through identification of immune correlates of protection.

Controlled human infection models (CHIM) have a strong track record in accelerating vaccine development, particularly for enteric pathogens [12,13]. For example, the live attenuated cholera

For peer review only - http://bmjopen.bmj.com/site/about/guidelines.xhtml

vaccine CVD 103-HgR was licensed as a travel vaccine on the basis of safety, immunogenicity and efficacy data arising from CHIM studies [14]. More recently, a programme of typhoidal *Salmonella* CHIM studies demonstrated efficacy of a Vi-tetanus toxoid (Vi-TT) conjugate vaccine against *S.* Typhi (Typbar-TCV, Bharat Biotech) [15]. These data contributed to recommendations from WHO SAGE to support programmatic use of Vi-TT in high burden settings and were supported by subsequent phase III trials demonstrating similar efficacy in Nepal, Bangladesh, and Malawi [16–18].

The CHANTS study has been funded by the Wellcome Trust to establish a first-in-human NTS CHIM, with the aim of addressing key knowledge gaps in dNTS and iNTS disease pathogenesis and immunity. We will conduct a double-blind, randomised, safety and dose-escalation study with the primary objective of determining the infectious dose required for 60-75% of participants to develop systemic salmonellosis. Successfully establishing a reproducible NTS CHIM could provide valuable insights into mechanisms of protective immunity and serve as a platform for future evaluation of candidate iNTS vaccines and therapeutics.

4 METHODS AND ANALYSIS

4.1 Study design

The CHANTS study is a double-blinded, randomised, quarantine-based, controlled human infection model which aims to establish the infectious dose required for healthy participants to develop salmonellosis following oral challenge with either *S.* Typhimurium ST313 (strain D23580) or ST19 (strain 4/74). A predicted total of 40-80 healthy adult volunteers who are eligible, and consenting, will be randomised 1:1 to receive oral challenge with either D23580 or 4/74. The study will be conducted at Imperial College London with clinical support from partner tertiary hospitals at Imperial College Healthcare NHS Trust.

4.2 Patient and public involvement

Patient and public engagement activities have been conducted throughout the protocol design stage, gaining valuable insights from the involvement of key stakeholders. Focus groups have been conducted with support of the NIHR Imperial Biomedical Research Centre patient experience research group. Discussions with members of the public in this forum – including past participants of previous

For peer review only - http://bmjopen.bmj.com/site/about/guidelines.xhtml

CHIMs – have identified key considerations for the safe and acceptable conduct of this trial and supported the development of clear and thorough participant-facing information to underpin informed consent. Future similar activities are planned in regions with high iNTS burden to establish acceptability of potential future transfer of an iNTS CHIM to settings most affected by the disease under investigation.

4.3 Study objectives and outcomes

The primary objective is to determine the minimum infectious dose in colony forming units (CFU) required for 60-75% of healthy participants to develop clinical or microbiological features of systemic salmonellosis following oral challenge with either *S.* Typhimurium D23580 or 4/74. Systemic salmonellosis is defined as the development of either: (1) fever ≥38°C on ≥2 occasions ≥12 hours apart, or (2) *S.* Typhimurium bacteraemia at any time point. As outlined above, exposure to *S.* Typhimurium can result in heterogeneous disease phenotypes, ranging from asymptomatic gastrointestinal colonisation to bloodstream infection. Secondary endpoints include determining the proportion of participants who develop outcomes along this disease spectrum, including *Salmonella* colonisation, diarrhoeal disease (dNTS), systemic symptoms, or severe salmonellosis. Exploratory study outcomes aim to describe the immune response to *S.* Typhimurium infection (Table 1: Primary, secondary, and exploratory study objectives and outcomes).

In this first-in-human study, we have a unique opportunity to elucidate mechanisms of bacterial virulence in dNTS and iNTS, identify immune correlates of protection, describe host-pathogen interactions, changes in host microbiota in response to infection, and develop novel diagnostic platforms.

4.4 Recruitment and eligibility

Several strategies will be employed to recruit study participants, including advertising throughout local hospitals, GP surgeries, and higher education institutions; media and website advertising; direct email to members of the NIHR Imperial Clinical Research Facility healthy volunteer database; direct mail-out and email to potentially eligible participants identified via National Health Applications and Infrastructure Services (NHAIS). Consenting healthy adult males and non-pregnant females aged 18-50 without pre-existing risk factors for severe iNTS or chronic infection will be recruited. Strict eligibility criteria have been designed to mitigate risks of severe disease, chronic carriage, or

For peer review only - http://bmjopen.bmj.com/site/about/guidelines.xhtml

development of focal seeded infection. Detailed screening will include comprehensive medical history, physical examination, baseline blood counts, biochemical profiles, screening for immunosuppression, and abdominal ultrasonography to exclude subclinical biliary or abdominal aortic disease (see supplementary Table 1 for full eligibility criteria). Individuals who have had documented prior *Salmonella* infection, or received live-attenuated oral Ty21a typhoid vaccine, will be excluded due to potential cross-protective immune responses against *S*. Typhimurium. Further temporary exclusion criteria will be reviewed prior to oral challenge to exclude intercurrent infection or antimicrobial use which may bias results (supplementary material Table 2).

4.5 Study interventions

Consenting participants who fulfil eligibility criteria will enter the study at Day -7 (7 days prior to oral bacterial challenge). Days 0-7 will take place under clinical observation in a quarantine research facility at Imperial College Healthcare NHS Trust, with subsequent daily out-patient reviews on Days 8-14, prior to long-term follow-up visits at Days 28, 90, 180, and 365. The overall participant journey is illustrated in Figure 1.

4.5.1 Challenge Strain Selection and Manufacture

Challenge strains have been selected to maximise future utility of the model in supporting iNTS vaccine development. Wild-type ST313 (D23580) and ST19 (4/74) are phylogenetically representative of contemporary disease-causing isolates, have a traceable history from isolation, and have undergone detailed genotypic, phenotypic, and transcriptomic characterisation [19,20]. Bacterial stocks have been manufactured to GMP standard by the Walter Reed Army Institute of Research, MD, USA, in collaboration with PATH (Seattle, USA), with strains originally drawn from the UKHSA National Collection of Type Cultures.

ST313 lineage 2 reference strain D23580 was originally isolated from a blood culture obtained from a 24-month-old HIV-negative child at Queen Elizabeth Central Hospital, Blantyre, Malawi, and is considered an archetypal isolate representative of strains causing iNTS in SSA. ST19 (4/74), originally isolated from a calf in England in 1974, is considered an archetypal strain causative of dNTS and representative of current clinical infections. D23580 harbours a distinct prophage and plasmid repertoire compared with 4/74, including a virulence plasmid pSLT encoding resistance to ampicillin,

For peer review only - http://bmjopen.bmj.com/site/about/guidelines.xhtml

chloramphenicol, streptomycin, sulphonamides, and trimethoprim [20]. Both strains are susceptible to ciprofloxacin, azithromycin and third generation cephalosporins, ensuring a range of treatment options are available for challenged participants. The rationale for strains selected for use in this model, and their relative merits, have been discussed in detail elsewhere [21].

4.5.2 Randomisation and Blinding

Block randomisation (block size = 10) to strain allocation will be carried out on or after Day -7. An initial sentinel cohort (n = 10) of will be randomised 1:1 (block sizes = 2, 4, 4) to receive a starting dose 1-5 x 10^3 CFU of either strain 4/74 or D23580. This starting dose has been determined based on prior experience from typhoidal CHIMs and continual reassessment method (CRM) dose prediction simulations (section 4.8) [15,22–24]. Unblinded attack rates and safety data will be reviewed by the Data Safety and Monitoring Committee (DSMC) prior to further participant randomisation (section 4.6). Randomisation lists will be generated and stored on a secure server accessible only by unblinded study team members. All clinical staff and participants will remain blinded to challenge allocation for the duration of the study.

4.5.3 Dose Escalation/De-escalation

Dose escalation and de-escalation decisions will be undertaken by the continual reassessment method (CRM). Escalation for both strains will occur in parallel, starting at 1-5 x 10^3 CFU, increasing to a maximum of 1-5 x 10^6 CFU or decreasing down to 1-5 x 10^1 CFU. Participants will be randomised to receive one of the two challenge agents at the dose determined by the CRM until target dosing is established for one agent. From that point, the remaining participants will be challenged with the other agent. We aim to recruit between 40-80 participants in total (20-40 for each challenge agent). The final challenge dose will be defined as one that achieves a 60-75% attack rate. The DSMC will be provided with an updated report after each cohort of 5 completes challenge. The dose escalation/de-escalation decision algorithm is illustrated in Figure 2.

4.5.4 Challenge procedures

Participants will receive oral bacterial challenge with either D23580 or 4/74 on study Day 0, following admission to an isolation side-room within the quarantine facility. Assessments will be conducted to confirm the absence of any temporary exclusion criteria (see supplementary Table 2) prior to

For peer review only - http://bmjopen.bmj.com/site/about/guidelines.xhtml

challenge. Both *S.* Typhimurium challenge agents used in this study will be administered via the oral route with sodium bicarbonate buffer to achieve gastric acid neutralisation. Participants will fast for 90 minutes before and after challenge. The solution for ingestion containing either wild type S. Typhimurium 4/74 or D23580 will be prepared in a Class II biological safety cabinet that is solely used for the purpose of preparing the challenge solution.

4.5.5 Clinical Assessment During Quarantine

Participants will be reviewed daily during admission to the quarantine facility by a clinical member of the study team. Extensive assessments will be undertaken, including physical examination, recording vital signs, assessment of mood, and review of participant symptom diaries. Blood, stool, and saliva specimen collection will be undertaken according to timelines outlined in supplementary table 3. Supportive therapy will be provided as required for symptom control e.g., oral rehydration solution or intravenous fluids, analgesia, and anti-emetics.

4.5.6 Infection Prevention and Control

Participants will be managed using infection control protocols appropriate for enteric infections. Study investigators will adhere to strict hand-hygiene policies of the research facility and utilise enteric personal protective equipment (PPE) for all study procedures. Participants will receive clear instruction on hand hygiene procedures – including illustrated guidance – to be adhered to during and after quarantine, in addition to guidance on cleaning protocols for bathrooms in the home environment following discharge. Participants will be nursed within single-occupancy side-rooms with ensuite toilet facilities for the duration of quarantine. The near-patient environment, including door handles, taps, toilet flushes and bed rails will be decontaminated daily throughout the quarantine period in accordance with local policies. Assessment of *Salmonella* environmental contamination within the treatment facility will be undertaken as an exploratory study objective, utilising culture, and molecular methods to detect the presence of *S.* Typhimurium in the participant's immediate surroundings and in high traffic clinical areas.

Close contacts of participants, including household contacts, will be offered the opportunity to be screened for *Salmonella* infection, which will involve obtaining three stool samples 48-hours apart a minimum of seven days after the participant has begun antibiotic treatment. If either stool culture of

For peer review only - http://bmjopen.bmj.com/site/about/guidelines.xhtml

a household contact is positive, he/she will be referred to a Consultant in Infectious Diseases for appropriate antibiotic management and the local health protection unit will be informed.

For peer review only

For peer review only - http://bmjopen.bmj.com/site/about/guidelines.xhtml

4.5.7 *Antimicrobial therapy*

Treatment of *Salmonella* infection in healthy immunocompetent adults poses a number of specific challenges. Antimicrobial therapy is indicated for invasive disease but may prolong shedding in patients with pauci-symptomatic gastroenteritis. We have elected to offer rescue treatment in specific circumstances. The rationale for this decision is discussed elsewhere [21]. Antibiotic therapy will be initiated in all participants who fulfil criteria for *Salmonella* diagnosis (SD) through reaching either or both of the defined primary endpoints of persistent fever or bloodstream infection. Treatment will also be commenced in participants who do not achieve the primary outcome measures but reach any of the other pre-specified treatment criteria outlined in Table 2. Treatment for bloodstream infection will comprise oral ciprofloxacin 500mg twice daily for 10-14 days, or second line therapy of intravenous ceftriaxone 1g once daily for up to 14 days. All other treatment indications (systemic illness, gastroenteritis, asymptomatic/pauci-symptomatic shedding) will be treated with oral ciprofloxacin 500mg twice daily for 5 days, or with second-line oral azithromycin 500mg once daily for 5 days. Antibiotic treatment will not be routinely offered to participants who do not fulfil any pre-specified treatment criteria. The full antibiotic treatment decision algorithm is illustrated in supplementary figure 1.

4.5.8 *Discharge from Quarantine*

The duration of quarantine will be 7 days from the time of oral challenge (168 hours). Participants will be eligible for discharge if they fulfil the criteria outlined in Table 3. Some participants may be discharged earlier than day 7 if they have been started on antibiotic therapy and completed 96 hours of follow-up after starting treatment. If these criteria are not met on day 7, then participants will be asked to remain in quarantine until discharge criteria are met. The discharge decision making algorithm is illustrated in supplementary figure 2.

Discharge criteria have been designed to maximise participant clinical safety in addition to minimising risk of secondary onward transmission. Current UKHSA guidance does not mandate the collection of clearance stool cultures following an episode of gastroenteritis secondary to NTS infection. Instead, the absence of diarrhoea for at least 48 hours is considered sufficient for release from isolation [25]. In this study, clearance stool cultures will be obtained to meet a secondary study objective of measuring predicted differences in patterns of shedding between 4/74 and D23580 strains.

For peer review only - http://bmjopen.bmj.com/site/about/guidelines.xhtml

4.6 Governance

An independent Data and Safety Monitoring Committee (DSMC) has been appointed to provide real-time oversight of safety and trial conduct. The DSMC will review unblinded safety data throughout the study according to a pre-defined DSMC charter. The committee will have access to data and, if required, will monitor these data, and make recommendations to the study investigators on whether there are any ethical or safety reasons the trial should not continue. The DSMC will review the attack rate and advise on dose escalation/de-escalation decisions. Study monitoring will also be undertaken by a Contract Research Organisation (CRO), separate to and independent from the DSMC, to ensure safety and compliance with the approved study protocol. Strict data management plans will be adhered to with compliance to Good Clinical Practice guidelines.

4.7 Safety

As with all controlled human infection models, maintenance of participant safety during and after the study is paramount. In this model, general risks associated with participation may include study-fatigue, repeated phlebotomy procedures, and consequences of isolation in quarantine. Risks more directly attributable to challenge agents include symptomatic infection, complications of antibiotic treatment (in the case of bacterial challenge), and the small risk of long-term sequelae. Potential risks have been given extensive consideration, with risk mitigation strategies devised to minimise possible impact on participants. The potential complications of participation – and risk mitigation strategies – have been discussed elsewhere [21]. In addition to participant safety, careful consideration has been given to ensure safety of study investigators and close contacts. Clear infection control protocols have been developed (as outlined above), in addition to close liaison with UKHSA to monitor and address any potential secondary transmission events, including within households following discharge from the quarantine facility.

4.8 Statistical analysis

This dose-finding study will use the continual reassessment method (CRM) to determine the target dose that can achieve a 60-75% attack rate [26,27]. To our knowledge, this will be the first dose-finding CHIM to employ CRM methodology. The advantages over traditional rule-based dose-finding studies are that the CRM method borrows information across all dose levels, which increases likelihood of

For peer review only - http://bmjopen.bmj.com/site/about/guidelines.xhtml

finding the true dose for a given sample size compared with rule-based methods in the majority of scenarios, particularly when the maximum sample size is small. There is no formal sample size calculation for this study. The maximum sample size will be 40 participants for each challenge strain. The operating characteristics of the CRM and the rule-based approach are illustrated in supplementary Table 4.

5 ETHICS AND DISSEMINATION

This study protocol has been reviewed and approved by the NHS Health Research Authority (London – Fulham Research Ethics Committee 21/PR/0051; IRAS 301659) and is registered on clinicaltrials.gov (registration number NCT05870150). The sponsor is Imperial College London, and the study is indemnified under institutional and NHS insurance policies. Our findings will be of significant international interest, with several potential future applications for assessment of iNTS vaccines and novel therapeutics. We will publish all findings in peer-reviewed journals, and ensure presentation at national and international conferences, in addition to close liaison with key stakeholders, participants and funders.

6 DISCUSSION

The global morbidity and mortality burden from iNTS disease remains unacceptably high, with the greatest impact concentrated in children and immunocompromised adults in sub-Saharan Africa. Effective vaccines are urgently required as a public health tool to avert cases and prevent expansion of antibiotic resistant strains. Vaccine development for iNTS is hampered – in part – by an incomplete understanding of mechanisms of protective immunity against natural infection. In addition, large-scale phase III trials for iNTS vaccines will require substantial time and financial commitments.

CHIMs offer a platform to support vaccine development, providing a means of obtaining early vaccine safety data and efficacy read-outs. As has been observed for other enteric pathogens – including typhoidal *Salmonella* serovars – demonstration of efficacy in the context of a CHIM can support vaccine licensure and aid selection of the most appropriate candidates for field testing, de-risking phase III studies. The success of typhoidal CHIM studies in demonstrating efficacy of the Vi-TT (Typbar TCV, Bharat Biotech) vaccine laid the groundwork for field trials in Asia and Africa. The CHANTS study aims to develop a safe and reproducible model of iNTS disease, identify immune correlates of

For peer review only - http://bmjopen.bmj.com/site/about/guidelines.xhtml

For peer review only

protection, elucidate mechanisms of bacterial virulence, and provide a platform for future iNTS vaccine randomised trials.

As a first-in-human model the CHANTS study provides a unique opportunity to improve our understanding of the pathobiology of infection with *S.* Typhimurium strains 4/74 and D23580. The randomised, double-blinded study design allows objective analysis of differing clinical phenotypes induced in healthy volunteers by two strains which cause significantly different disease phenotypes in field settings. We hypothesise that ST313 (D23580) will be associated with an invasive phenotype compared with the enterocolitis anticipated as a result of challenge with ST19 (4/74).

Longitudinal descriptions of humoral, cellular, and mucosal immune responses to infection will provide detailed insights into mechanisms of protective immunity. Identification of these immune correlates of protection can support and inform future iNTS vaccine design. Furthermore, gaining a deeper understanding of bacterial virulence mechanisms through transcriptomic analyses can support future vaccination and therapeutic strategies.

The primary limitations of the CHANTS study – shared with most controlled human infection models – stem from undertaking the study in a healthy adult volunteer group which may not accurately reflect natural disease acquisition and pathogenesis. There are clear host factor differences between this group and individuals most susceptible to iNTS disease. However, as with prior CHIMs undertaken in healthy participants, we anticipate this infection model will generate key insights into *Salmonella* infection biology with future translational applications. Successful development of an *S.* Typhimurium CHIM, with an established safety profile and infectious dose, could be transferred to an endemic setting for future investigation in a population more closely resembling those at greatest risk of disease, as has been achieved previously in a range of enteric and non-enteric pathogen challenge models [28–32].

In addition to inherent differences between our study population and those at greatest risk of iNTS, there are limitations in achievable study endpoints. Our primary endpoint of persistent fever, with or without bloodstream infection, does not necessarily reflect the severe clinical phenotypes an iNTS vaccine would aim to protect against in field settings. However, in view of the heterogeneous clinical phenotypes associated with NTS infection, ranging from asymptomatic colonisation through to septic shock, this endpoint is felt to represent the optimal trade-off between clinical manifestations of disease and patient safety within the context of a CHIM. The relative merits of endpoint selection and their rationale have been discussed previously [21].

For peer review only - http://bmjopen.bmj.com/site/about/guidelines.xhtml

In summary, the CHANTS study presents a valuable opportunity to improve our understanding of iNTS disease and support vaccine development efforts. Whilst there are limitations, insights gained from this first-in-human model can inform future iNTS vaccine design and provide a platform for future randomised trials. Successful development of an *S.* Typhimurium CHIM could lead to future investigations in populations more closely resembling those at greatest risk of disease.

For peer review only

For peer review only - http://bmjopen.bmj.com/site/about/guidelines.xhtml

7 REFERENCES

[1] Roth GA, Abate D, Abate KH, Abay SM, Abbafati C, Abbasi N, et al. Global, regional, and national age-sex-specific mortality for 282 causes of death in 195 countries and territories, 1980–2017: a systematic analysis for the Global Burden of Disease Study 2017. The Lancet 2018;392:1736–88. https://doi.org/10.1016/S0140-6736(18)32203-7.

[2] Feasey NA, Dougan G, Kingsley RA, Heyderman RS, Gordon MA. Invasive non-typhoidal salmonella disease: an emerging and neglected tropical disease in Africa. Lancet 2012;379:2489–99. https://doi.org/10.1016/S0140-6736(11)61752-2.

[3] GBD 2017 Non-Typhoidal Salmonella Invasive Disease Collaborators. The global burden of non-typhoidal salmonella invasive disease: a systematic analysis for the Global Burden of Disease Study 2017. Lancet Infect Dis 2019;19:1312–24. https://doi.org/10.1016/S1473-3099(19)30418-9.

[4] Feasey NA, Everett D, Faragher EB, Roca-Feltrer A, Kang'ombe A, Denis B, et al. Modelling the Contributions of Malaria, HIV, Malnutrition and Rainfall to the Decline in Paediatric Invasive Non-typhoidal Salmonella Disease in Malawi. PLoS Negl Trop Dis 2015;9:e0003979. https://doi.org/10.1371/journal.pntd.0003979.

[5] Tacconelli E, Carrara E, Savoldi A, Harbarth S, Mendelson M, Monnet DL, et al. Discovery, research, and development of new antibiotics: the WHO priority list of antibiotic-resistant bacteria and tuberculosis. Lancet Infect Dis 2018;18:318–27. https://doi.org/10.1016/S1473-3099(17)30753-3.

[6] Uche IV, MacLennan CA, Saul A. A Systematic Review of the Incidence, Risk Factors and Case Fatality Rates of Invasive Nontyphoidal Salmonella (iNTS) Disease in Africa (1966 to 2014). PLoS Negl Trop Dis 2017;11:e0005118. https://doi.org/10.1371/journal.pntd.0005118.

[7] Ao TT, Feasey NA, Gordon MA, Keddy KH, Angulo FJ, Crump JA. Global burden of invasive nontyphoidal Salmonella disease, 2010(1). Emerg Infect Dis 2015;21. https://doi.org/10.3201/eid2106.140999.

[8] Marchello CS, Fiorino F, Pettini E, Crump JA, Vacc-iNTS Consortium Collaborators. Incidence of non-typhoidal Salmonella invasive disease: A systematic review and meta-analysis. J Infect 2021;83:523–32. https://doi.org/10.1016/j.jinf.2021.06.029.

[9] Pulford CV, Perez-Sepulveda BM, Canals R, Bevington JA, Bengtsson RJ, Wenner N, et al. Stepwise evolution of Salmonella Typhimurium ST313 causing bloodstream infection in Africa. Nat Microbiol 2021;6:327–38. https://doi.org/10.1038/s41564-020-00836-1.

[10] Read the report : Vaccines for AMR n.d. https://vaccinesforamr.org/read-the-report/ (accessed April 27, 2023).

[11] Baliban SM, Lu Y-J, Malley R. Overview of the Nontyphoidal and Paratyphoidal Salmonella Vaccine Pipeline: Current Status and Future Prospects. Clin Infect Dis 2020;71:S151–4. https://doi.org/10.1093/cid/ciaa514.

[12] Choy RKM, Bourgeois AL, Ockenhouse CF, Walker RI, Sheets RL, Flores J. Controlled Human Infection Models To Accelerate Vaccine Development. Clin Microbiol Rev 2022;35:e0000821. https://doi.org/10.1128/cmr.00008-21.

[13] Roestenberg M, Hoogerwerf M-A, Ferreira DM, Mordmüller B, Yazdanbakhsh M. Experimental infection of human volunteers. Lancet Infect Dis 2018;18:e312–22. https://doi.org/10.1016/S1473-3099(18)30177-4.

[14] Chen WH, Cohen MB, Kirkpatrick BD, Brady RC, Galloway D, Gurwith M, et al. Single-dose Live Oral Cholera Vaccine CVD 103-HgR Protects Against Human Experimental Infection With Vibrio cholerae O1 El Tor. Clin Infect Dis 2016;62:1329–35. https://doi.org/10.1093/cid/ciw145.

[15] Jin C, Gibani MM, Moore M, Juel HB, Jones E, Meiring J, et al. Efficacy and immunogenicity of a Vi-tetanus toxoid conjugate vaccine in the prevention of typhoid fever using a controlled human

For peer review only - http://bmjopen.bmj.com/site/about/guidelines.xhtml

infection model of Salmonella Typhi: a randomised controlled, phase 2b trial. The Lancet 2017;390:2472–80. https://doi.org/10.1016/S0140-6736(17)32149-9.
[16] Shakya M, Colin-Jones R, Theiss-Nyland K, Voysey M, Pant D, Smith N, et al. Phase 3 Efficacy Analysis of a Typhoid Conjugate Vaccine Trial in Nepal. N Engl J Med 2019;381:2209–18. https://doi.org/10.1056/NEJMoa1905047.
[17] Patel PD, Patel P, Liang Y, Meiring JE, Misiri T, Mwakiseghile F, et al. Safety and Efficacy of a Typhoid Conjugate Vaccine in Malawian Children. N Engl J Med 2021;385:1104–15. https://doi.org/10.1056/NEJMoa2035916.
[18] Qadri F, Khanam F, Liu X, Theiss-Nyland K, Biswas PK, Bhuiyan AI, et al. Protection by vaccination of children against typhoid fever with a Vi-tetanus toxoid conjugate vaccine in urban Bangladesh: a cluster-randomised trial. The Lancet 2021;398:675–84. https://doi.org/10.1016/S0140-6736(21)01124-7.
[19] Kröger C, Colgan A, Srikumar S, Händler K, Sivasankaran SK, Hammarlöf DL, et al. An infection-relevant transcriptomic compendium for Salmonella enterica Serovar Typhimurium. Cell Host Microbe 2013;14:683–95. https://doi.org/10.1016/j.chom.2013.11.010.
[20] Canals R, Hammarlöf DL, Kröger C, Owen SV, Fong WY, Lacharme-Lora L, et al. Adding function to the genome of African Salmonella Typhimurium ST313 strain D23580. PLoS Biol 2019;17:e3000059. https://doi.org/10.1371/journal.pbio.3000059.
[21] Smith C, Smith E, Chiu C, Hinton J, Perez Sepulveda B, Gordon M, et al. The Challenge Non-Typhoidal Salmonella (CHANTS) Consortium: Development of a non-typhoidal Salmonella controlled human infection model: Report from a consultation group workshop, 05 July 2022, London, UK. Wellcome Open Res 2023;8:111. https://doi.org/10.12688/wellcomeopenres.19012.1.
[22] Gibani MM, Jin C, Shrestha S, Moore M, Norman L, Voysey M, et al. Homologous and heterologous re-challenge with Salmonella Typhi and Salmonella Paratyphi A in a randomised controlled human infection model. PLoS Negl Trop Dis 2020;14:e0008783. https://doi.org/10.1371/journal.pntd.0008783.
[23] Gibani MM, Jones E, Barton A, Jin C, Meek J, Camara S, et al. Investigation of the role of typhoid toxin in acute typhoid fever in a human challenge model. Nat Med 2019;25:1082–8. https://doi.org/10.1038/s41591-019-0505-4.
[24] Dobinson HC, Gibani MM, Jones C, Thomaides-Brears HB, Voysey M, Darton TC, et al. Evaluation of the Clinical and Microbiological Response to Salmonella Paratyphi A Infection in the First Paratyphoid Human Challenge Model. Clinical Infectious Diseases 2017;64:1066–73. https://doi.org/10.1093/cid/cix042.
[25] Recommendations for the Public Health Management of Gastrointestinal Infections 2019 2020.
[26] O'Quigley J, Pepe M, Fisher L. Continual reassessment method: a practical design for phase 1 clinical trials in cancer. Biometrics 1990;46:33–48.
[27] Wheeler GM, Mander AP, Bedding A, Brock K, Cornelius V, Grieve AP, et al. How to design a dose-finding study using the continual reassessment method. BMC Med Res Methodol 2019;19:18. https://doi.org/10.1186/s12874-018-0638-z.
[28] Morton B, Jambo K, Chikaonda T, Rylance J, Henrion MYR, Banda NP, et al. The influence of pneumococcal conjugate vaccine-13 on nasal colonisation in a controlled human infection model of pneumococcal carriage in Malawi: a double-blinded randomised controlled trial protocol. Wellcome Open Res 2021;6:240. https://doi.org/10.12688/wellcomeopenres.17172.2.
[29] Kapulu MC, Kimani D, Njuguna P, Hamaluba M, Otieno E, Kimathi R, et al. Controlled human malaria infection (CHMI) outcomes in Kenyan adults is associated with prior history of malaria exposure and anti-schizont antibody response. BMC Infect Dis 2022;22:86. https://doi.org/10.1186/s12879-022-07044-8.
[30] Pitisuttithum P, Cohen MB, Phonrat B, Suthisarnsuntorn U, Bussaratid V, Desakorn V, et al. A human volunteer challenge model using frozen bacteria of the new epidemic serotype, V.

For peer review only - http://bmjopen.bmj.com/site/about/guidelines.xhtml

cholerae O139 in Thai volunteers. Vaccine 2001;20:920–5. https://doi.org/10.1016/s0264-410x(01)00381-4.
[31] Pitisuttithum P, Islam D, Chamnanchanunt S, Ruamsap N, Khantapura P, Kaewkungwal J, et al. Clinical Trial of an Oral Live Shigella sonnei Vaccine Candidate, WRSS1, in Thai Adults. Clin Vaccine Immunol 2016;23:564–75. https://doi.org/10.1128/CVI.00665-15.
[32] Bodhidatta L, Pitisuttithum P, Chamnanchanant S, Chang KT, Islam D, Bussaratid V, et al. Establishment of a Shigella sonnei human challenge model in Thailand. Vaccine 2012;30:7040–5. https://doi.org/10.1016/j.vaccine.2012.09.061.

Authors Contributions

Conceptualisation and study design: CS, ES, AR, RV, JCDH, MAG, RKMC, XL, AJP, CC, GSC, MMG

Data analysis plan: CS, XL, GSC, MMG

Drafting of protocol: CS, ES, AR, RV, GSC, MMG

Protocol amendments: CS, RKMC, AJP, XL, MMG

Funding

The CHANTS study is funded by The Wellcome Trust (grant number 224029/Z/21/Z: awarded to principal investigator MMG). CS, GSC, CC and MMG are supported, in part, by the National Institute of Health Research Imperial Biomedical Research Centre.

Disclaimer

The funder of the study has had no role in study design, and will have no role in data collection, data analysis, data interpretation, writing of the report or decision to submit.

Competing Interests

None declared.

Word Count

Main Text: 3667

For peer review only - http://bmjopen.bmj.com/site/about/guidelines.xhtml

8 TABLES

Table 1: Primary, secondary, and exploratory study objectives and outcomes

	Objective(s)	Outcome(s)/Endpoint(s)
**Primary**	To determine the minimum infectious dose (CFU) of *S.* Typhimurium strains 4/74 and D23580 required for 60-75% of volunteers to develop systemic Salmonellosis following oral challenge.	The proportion of participants who develop:<br>1. Fever ≥38°C on ≥2 occasions ≥12 hours apart **AND/OR**<br>2. *Salmonella* Typhimurium bacteraemia at any time point
**Secondary**	1. To describe *S.* Typhimurium colonisation rate following oral challenge with *Salmonella* Typhimurium 4/74 and D23580 strains at different doses.	The proportion from whom *Salmonella* Typhimurium is isolated from stool on ≥2 occasions ≥48 hours from challenge at different doses of each strain.
	2. To describe the *S.* Typhimurium gastroenteritis rate following oral challenge of *Salmonella* Typhimurium 4/74 and D23580 strains at different doses.	The proportion of participants at different doses at each strain developing:<br>1 Severe diarrhoea* **and/or**<br>2 Moderate diarrhoea** **plus**<br>2.1 Fever ≥38°C on ≥1 occasion **and/or**<br>2.2 ≥1 Grade 2 gastrointestinal symptoms (abdominal pain, nausea, vomiting, tenesmus)
	3. To determine the persistent fever rate following oral challenge with *S.* Typhimurium 4/74 and D23580 at different doses.	The proportion of participants who develop fever ≥38°C on ≥2 occasions ≥12 hours apart at different doses of each strain.
	4. To describe the *S.* Typhimurium bacteraemia rate following oral challenge with *Salmonella* Typhimurium 4/74 and D23580 at different doses.	The proportion of participants developing *Salmonella* Typhimurium bacteraemia at any timepoint following challenge at different doses of each strain.

For peer review only - http://bmjopen.bmj.com/site/about/guidelines.xhtml

	5. To describe the rate of systemic *Salmonellosis* according to **an alternative composite diagnostic criterio**n following oral challenge of *Salmonella* Typhimurium 4/74 and D23580 at different doses.	The proportion of participants meeting the criteria for a composite diagnosis of *Salmonellosis* at different doses of each strain defined as any of:<br>1 *S.* Typhimurium is isolated from stool on ≥2 occasions ≥48 hours from challenge **and/or**<br>2 Gastroenteritis **and/or**<br>3 Fever ≥38°C on ≥2 occasions ≥12 hours apart **and/or**<br>4 *S.* Typhimurium bacteraemia
	6. To describe the safety of oral challenge with *S*. Typhimurium 4/74 and D23580 strains.	The proportion of participants at different doses of each strain reporting: adverse events, adverse events of special interest, SAEs, SUSARs, concomitant medication usage.
	7. To compare **clinical** features following oral challenge with *S.* Typhimurium strains 4/74 or D23580.	A comparison of the clinical features of *Salmonella* infection after challenge with 4/74 or D23580 strains, with specific reference to the proportion of participants in each group developing:<br>1 Diarrhoea (volume, frequency, consistency, gastrointestinal symptom severity score, time to onset)<br>2 Any fever ≥38°C (including fever clearance time)<br>3 Solicited symptoms at any time: headache, malaise, anorexia, abdominal pain, nausea, vomiting, dysentery, myalgia, arthralgia, cough, rash (including measurement of severity, and duration from onset to resolution)
	4 To compare **microbiological** features of **gastrointestinal infection** following oral challenge with *S.* Typhimurium 4/74 or D23580.	Gastrointestinal *Salmonella* infection after challenge with 4/74 or D23580 strains, with specific reference to:<br>1 Time to onset in days in each group from challenge to first stool sample positive for *Salmonella* Typhimurium by culture and/or PCR<br>2 Duration of stool shedding in each group measured in days from first stool sample positive for *Salmonella.*<br>3 The duration of stool shedding in each group measured in days from first stool sample positive by culture and/or for *Salmonella* Typhimurium to first persistently negative stool sample (defined as three consecutive samples negative at least 48 hours apart)<br>4 The magnitude of stool shedding measured in CFU/ml in quantitative stool culture analysis
	5 To compare **microbiological** features of **bloodstream infection** following oral challenge with *S.* Typhimurium 4/74 or *S.* Typhimurium D23580 strains	*Salmonella* bloodstream infection after challenge with 4/74 or D23580 strains, with specific reference to:<br>1 The proportion of participants in each group developing any bacteraemia

For peer review only - http://bmjopen.bmj.com/site/about/guidelines.xhtml

		2 The severity of bacteraemia in each group as measured by: time to onset, duration, clearance time, magnitude quantified in CFU/ml.
	6 To compare biochemical and haematological laboratory parameters following oral challenge with *S.* Typhimurium 4/74 or D23580 strains.	Absolute values of laboratory parameters from time of challenge to Day 28 and/or Day 90, with specific reference to: Haemoglobin, white cell count and differential, urea and electrolytes, liver function tests.
	7 To describe and compare the serum antibody response following oral challenge with *S.* Typhimurium 4/74 or D23580 strains.	Absolute values of laboratory assays on serum samples measured at baseline and post challenge time points (e.g. Day 7, 14 , 28, 90 and 365), including but not limited to:<br>1 *S*. Typhimurium O-specific polysaccharide serum IgG and IgA concentration measured by ELISA<br>2 *S*. Typhimurium specific serum IgG and IgA concentration against other S. Typhimurium antigens measured by ELISA<br>3 Serum bactericidal antibody titres against *Salmonella* Typhimurium<br>4 *S*. Typhimurium specific antibody secreting cell and memory B-cell responses measured by ELISPOT<br>5 Other functional antibody activity measurements including systems serology platforms.
	8 To describe and compare the mucosal antibody response following oral challenge with *S.* Typhimurium 4/74 or D23580 strains.	Absolute values of laboratory assays on saliva and/or stool samples measured at baseline and post challenge time points (e.g. Day 7, 14, 28, 90 and 365) using laboratory assays including (but not limited to):<br>1 S. Typhimurium specific IgG and IgA concentration measured by ELISA<br>2 Bactericidal antibody titres<br>3 Other functional antibody activity measurements including systems serology platforms
	9 To describe and compare cell-mediated immune response following oral challenge with *S.* Typhimurium 4/74 D23580 strains.	Absolute values and inter-group comparison of laboratory assays performed on peripheral blood mononuclear cells measured at baseline and post challenge time points including (but not limited to):<br>1 Description of lymphocyte populations at baseline and following challenge as measured by flow cytometry and/or CyTOF<br>2 Frequency and magnitude of *S*. Typhimurium specific cell-mediated immune responses as measured by ELISPOT and flow cytometry and/or CyTOF

For peer review only

For peer review only - http://bmjopen.bmj.com/site/about/guidelines.xhtml

	10	To describe participant experience following oral challenge with *S.* Typhimurium 4/74 or D23580 strains	Descriptive statistics following administration of a structured questionnaire at baseline and post-challenge.
**Exploratory**	1	To investigate how the human microbiota interacts with oral challenge of *S.* Typhimurium 4/74 or D23580 strains	Metagenomic analysis of stool and/or saliva to measure constituent microbiological flora (and their metabolome/transcriptome) at baseline and post-challenge time points
	2	To investigate *Salmonella* Typhimurium virulence factors and gene expression	Exploratory analysis of stool samples at several time points following challenge to assess bacterial transcriptome by RNA-seq
	3	To describe and compare additional host immune responses following oral challenge of *S.* Typhimurium 4/74 or D23580 strains.	Analysis of B-cell repertoire, plasma cytokine profile and kinetics.
	4	To evaluate environmental contamination with *Salmonella enterica* in the near patient environment	The pattern and proportion of environmental samples testing positive for *Salmonella* Typhimurium by culture or molecular methods.
	5	To evaluate host-pathogen interactions following *Salmonella* infection including the presence and mechanisms of *Salmonella* persister infected macrophages.	Exploratory analysis of infected immune cells from blood including, but not limited to, single-cell RNA-seq.

*Severe gastroenteritis: ≥6 loose/liquid stools (Bristol Type 6-7) **and/or** >800 g of loose/liquid stools in a rolling 24-hour period **and/or** ≥2 stools with gross blood in 24 hours

**Moderate gastroenteritis: 4–5 loose/liquid stools (Bristol Type 6-7) and/or 400–800 g in a rolling 24-hour period

For peer review only - http://bmjopen.bmj.com/site/about/guidelines.xhtml

Table 2: Criteria for commencing antibiotic therapy following oral bacterial challenge

**Antibiotics are commenced if any of the following apply**
Any participant with *Salmonella* Typhimurium bacteraemia
Fever ≥38°C for ≥12hrs
Any participant with severe gastroenteritis*
Moderate gastroenteritis** plus:<br>• fever ≥38°C on one occasion and/or<br>• ≥1 Grade 2*** systemic symptoms
Any participant with 3 or more of the following symptoms on the same day at Grade 2 or higher.<br>• Headache<br>• Fatigue/Malaise<br>• Anorexia<br>• Abdominal pain<br>• Nausea<br>• Vomiting<br>• Myalgia<br>• Arthralgia<br>• Cough
Any participant from whom *Salmonella* has been detected from at least two stool culture/PCR and 24hrs apart who has not received antibiotics by day 14 post-challenge
Any participant in whom antibiotic use is felt to be clinically necessary (as decided by a medically qualified study doctor)
**Notes:**<br>*Severe gastroenteritis: ≥6 loose/liquid stools (Bristol Type 6-7) **and/or** >800 g of loose/liquid stools in a rolling 24-hour period **and/or** ≥2 stools with gross blood in 24 hours<br>**Moderate gastroenteritis: 4–5 loose/liquid stools (Bristol Type 6-7) and/or 400–800 g in a rolling 24-hour period<br>***Grade 0 = not present; Grade 1 = Present but no interference with activity; Grade 2 = Some interference with activity; Grade 3 = Significant; prevents daily activity; Grade 4: ED visit or hospitalisation

For peer review only

For peer review only - http://bmjopen.bmj.com/site/about/guidelines.xhtml

Table 3: Quarantine discharge criteria

**Participants will be discharged from the inpatient quarantine unit if the following criteria are met:**
• Medically fit for discharge in the opinion of the study physician **AND**
• Complete resolution of diarrhoea (Bristol stool type 6-7) for 48 hours **AND**
• Seven days (168 hours) have elapsed since challenge
**For patients diagnosed with Salmonellosis from day 0 to day 7, the following criteria apply:**
• Antibiotic treatment has been initiated and patient has completed 96hrs follow up
• Resolution of *Salmonella* Typhimurium bacteraemia (if applicable)

For peer review only

For peer review only - http://bmjopen.bmj.com/site/about/guidelines.xhtml

Figure 1: Overview of the participant journey from screening to study completion at 1 year of follow-up.

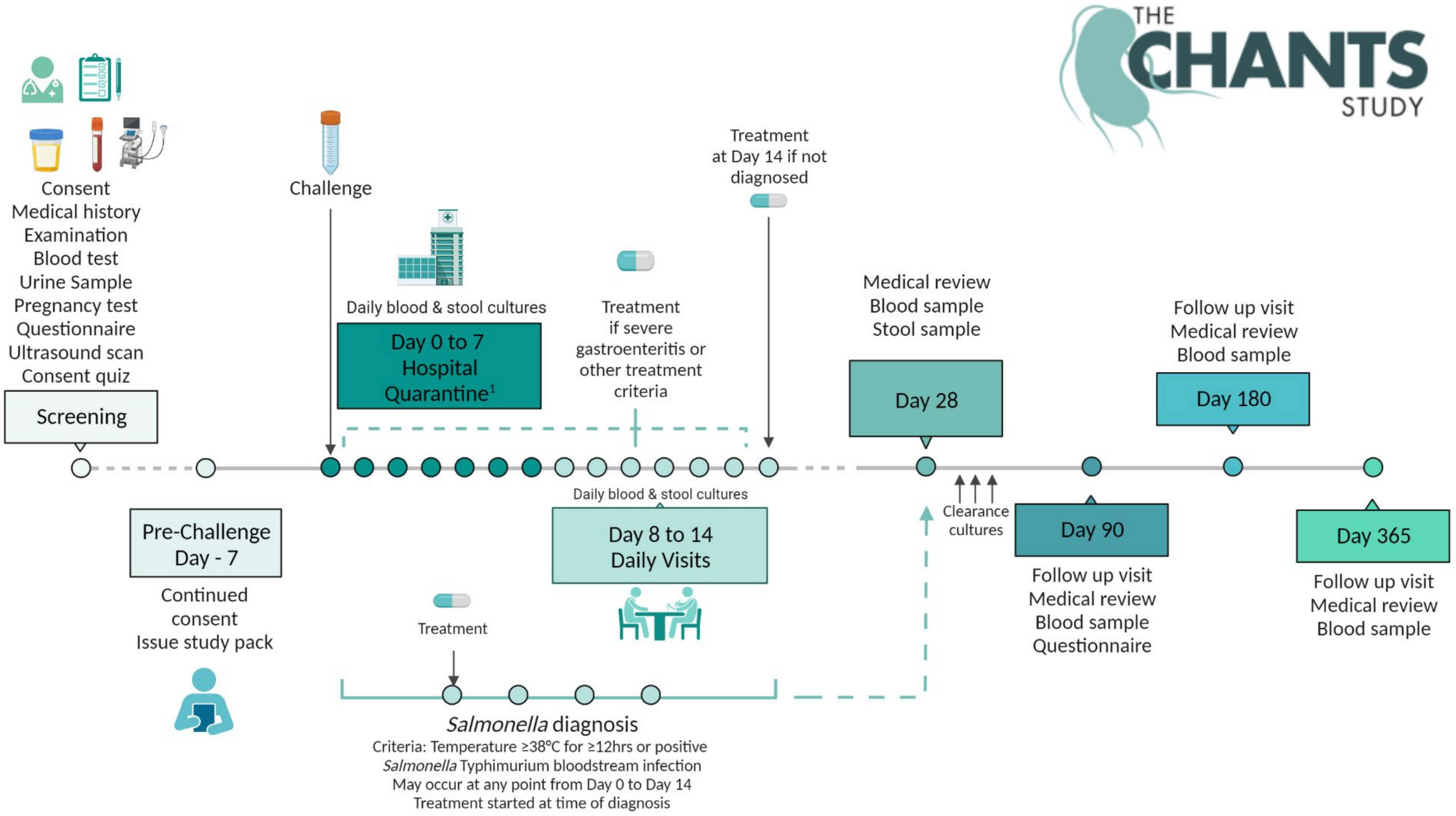

Figure 2: Decision making algorithm for dose escalation/de-escalation. Starting at 1-5 x10^3 to reach the primary endpoint. *The target dose for the first cohort will be 1-5 x 10^3 CFU, and the first cohort of 5 participants will be divided into 3 groups (1, 2, and 2 participants) and challenge with an interval of at least 7 days. For the rest of the cohorts, they will be challenged on the same day, if possible.

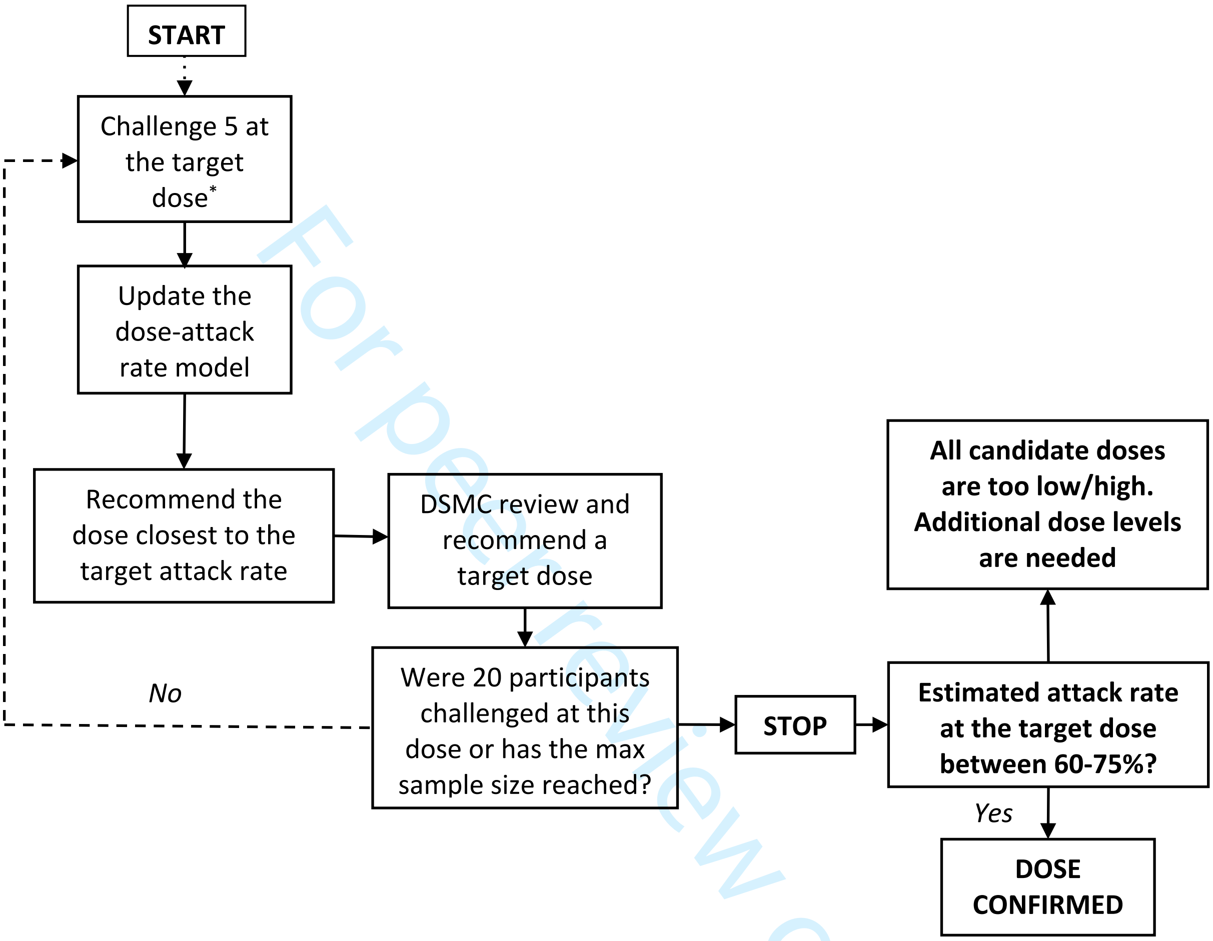

For peer review only

Supplementary Materials

Table 1: Eligibility criteria for participation

**Inclusion Criteria**
Agree to give informed consent for participation in the study.
Aged between 18 and 50 years inclusive at time of challenge.
In good health as determined by medical history, physical examination, and clinical judgment of the study team.
Agree (in the study team's opinion) to comply with all study requirements, including capacity to adhere to good personal hygiene and infection control precautions.
Agree to allow his or her General Practitioner (and/or Consultant if appropriate), to be notified of participation in the study.
Agree to allow study staff to contact his or her GP to access the participant's medical history and vaccination records.
Agree to allow UKHSA and the local health protection unit to be informed of their participation in the study.
Agree to give his or her close contacts written information informing them of the participant's involvement in the study and offer them voluntary screening for Salmonella carriage.
Agree to a period of inpatient quarantine whilst symptomatic (In exceptional circumstances, the duration of inpatient quarantine may be extended.)
Agree to have 24-hour contact with study staff during the four weeks post challenge and can ensure that they are contactable by mobile phone for the duration of the study period until antibiotic completion.
Agree to allow the study team to hold the name and 24-hour contact number of a close friend, relative or housemate who will be kept informed of the study participant's whereabouts for the duration of the challenge period after quarantine (from the time of challenge until completion of antibiotic course). This person will be contacted if study staff are unable to contact the participant after discharge from inpatient quarantine.
Have internet access to allow completion of the e-diary and real-time safety monitoring.
Agree to avoid antipyretic/anti-inflammatory treatment from the time of challenge (Day 0) until advised by a study doctor or until 14 days after challenge.
Agree to refrain from donating blood for the duration of the study.
Agree to provide their National Insurance/Passport number for the purposes of Trial over-volunteering prevention system (TOPS) registration and for payment of reimbursement expenses.
Participants must have received at least two doses of a SARS-CoV-2 vaccines that has been approved for use by the MHRA (or other national regulatory authority) four weeks prior to enrolment.
Proficient in English at a level sufficient to understand, retain, weigh up and communicate the study details as outlined in the participant information sheet, in the opinion of the study investigator.
**Exclusion Criteria**
History of microbiologically confirmed *Salmonella* infection[1].
History of receipt of oral Ty21a typhoid vaccination
History of **significant[2]** organ-specific and/or systemic disease that could interfere with trial conduct or completion. Including, for example, but not restricted to:

For peer review only

- Cardiovascular disease (ischaemic heart disease, valvular heart disease, vascular disease including aneurysmal disease or endovascular prostheses, prolonged corrected QT interval (>450 milliseconds) on ECG screening).<br>- Respiratory disease[3]<br>- Haematological disease including sickle cell disease and sickle cell trait.<br>- Endocrine disorders, including specifically diabetes mellitus.<br>- Renal or bladder disease, including history of renal calculi.<br>- Biliary tract disease, including specifically colic, asymptomatic gallstones, cholecystectomy.<br>- Gastro-intestinal disease including specifically: a current requirement for antacids, H_2-receptor antagonists, proton pump inhibitors or laxatives, Inflammatory bowel disease, confirmed diagnosis of irritable bowel syndrome as defined by the Rome IV criteria,<br>- Neurological disease<br>- Metabolic disease<br>- Autoimmune disease<br>- Psychiatric illness requiring hospitalisation.<br>- Known or suspected drug and/or alcohol misuse disorder<br>- Chronic/Active Infectious disease including active tuberculosis.<br>- Severe infection requiring hospitalisation for intravenous antibiotics within the last 10 years. Exceptions to this would include a short course of intravenous antibiotics for appendicitis, biliary sepsis, diverticulitis, and cellulitis.<br>- History of malaria infection<br>- History of joint replacement<br>- History of any orthopaedic/osseous implanted prosthesis<br>- Presence of any other internal implanted device e.g., permanent pacemaker.
Have any known or suspected impairment of immune function (as defined in the green book[140]), alteration of immune function, or prior immune exposure that may alter immune function to *Salmonella* infection resulting from, for example:<br>- Congenital or acquired immunodeficiency, including IgA deficiency.<br>- Human Immunodeficiency Virus infection or symptoms/signs suggestive of an HIV-associated condition<br>- Evidence of severe primary immunodeficiency, for example, severe combined immunodeficiency, Wiskott-Aldrich syndrome, and other combined immunodeficiency syndromes.<br>- Currently being treated for malignant disease with immunosuppressive chemotherapy or radiotherapy, or who have received such treatment within at least the last six months.<br>- Individuals who have received a solid organ transplant and are currently on immunosuppressive treatment.<br>- Individuals who have received a bone marrow transplant, until within 12 months of finishing all immunosuppressive treatment,<br>- Individuals receiving systemic high dose steroids[4] until at least three months after treatment has stopped.<br>- Individuals receiving other types of immunosuppressive[5] drugs (alone or in combination with lower doses of steroids) until at least six months after terminating such treatment.<br>- Receipt of immunoglobulin or any blood product transfusion within 3 months of study start.<br>- History of cancer (except squamous cell or basal cell carcinoma of the skin and cervical carcinoma in situ).
Moderate or severe depression or anxiety as classified by the Hospital Anxiety and Depression Score at screening or challenge that is deemed clinically significant by the study doctors[6].
Weight less than 50kg[7]

For peer review only

Anyone taking long-term medication (e.g., analgesia, anti-inflammatories, or antibiotics) that may affect symptom reporting or interpretation of the study results.
Contraindication to cephalosporin, fluroquinolone, or macrolide antibiotics.
Female participants who are pregnant, lactating or who are unwilling to ensure that they or their partner use effective contraception 30 days prior to challenge and continue to do so until two negative stool samples, a minimum of 3 weeks after completion of antibiotic treatment, have been obtained.
Full-time, part-time, or voluntary occupations involving:<br>- Clinical healthcare work[8]<br>- Social work with direct contact with young children (defined as those attending pre-school groups or nursery or aged under 2 years), or other clinically vulnerable children, adolescents.<br>- Clinical or social work with direct contact with highly susceptible patients or persons in whom *Salmonella* infection would have particularly serious consequences (unless willing to avoid work until demonstrated not to be infected with *Salmonella* in accordance with guidance from Public Health England and willing to allow study staff to inform their employer).
Full time, part time or voluntary occupations involving:<br>- Commercial food handling (involving preparing or serving unwrapped foods not subjected to further heating)
Close household contact with:<br>- Young children (defined as those attending pre-school groups, nursery or those aged less than 2 years)<br>- Any individual who is immunocompromised.<br>- Pregnant women<br>- Household contacts aged over 70 years
Scheduled elective surgery or other procedures requiring general anaesthesia during the study period.
Participants who have participated in another research study involving an investigational product that might affect risk of *Salmonella* infection or compromise the integrity of the study within the 30 days prior to enrolment (e.g. significant volumes of blood already taken in previous study)[9].
Detection of any abnormal results from screening investigations, unless deemed not clinically significant.
Any other social, psychological or health issues which, in the opinion of the study staff, may:<br>- Put the participant or their contacts at risk because of participation in the study.<br>- Adversely affect the interpretation of the primary endpoint data.<br>- Impair the participant's ability to participate in the study.
Having previously received any experimental *Salmonella* vaccine as part of a clinical trial
Have participated in previous *Salmonella* Typhi or Paratyphi challenge studies (with ingestion of challenge agent).
Any employee of the sponsor or research site personnel directly affiliated with this study or their immediate family members.
Inability to comply with any of the study requirements (at the discretion of the study staff).

Including enteric fever (*Salmonella* Typhi and/or *Salmonella* Paratyphi infection)

[2] Individuals with specific medical co-morbidities (e.g., childhood asthma) will be considered for enrolment after review with the principal investigator and clinical oversight group, following discussion with their GP and/or consultant as appropriate. The rationale for enrolment in these cases will be recorded in the CRF.

[3] Participants with well controlled asthma may be considered for enrolment at the discretion of the study investigators and following consultation with their general practitioner.

For peer review only - http://bmjopen.bmj.com/site/about/guidelines.xhtml

[4] Defined as those receiving at least 40mg of prednisolone per day for more than one week or equivalent.

[5] Azathioprine, cyclosporin, methotrexate, cyclophosphamide, leflunomide and other cytokine inhibitors.

[6] If elevated scores are due to temporary significant life events, the questionnaire may be repeated after resolution of the event with a view to inclusion if normal.

[7] Or a Body Mass Index (BMI) that, in the opinion of the study doctors, may adversely impair the interpretation of the study results or affect the safe performance of any study procedures.

[8] This includes medical, dental, nursing or midwifery students (or other allied-healthcare professionals) with direct patient contact. Such students who are not undertaking patient-facing rotations until demonstrated not to be infected with *Salmonella* species may be considered for enrolment.

[9] As assessed by both participant questioning and registration with The Over Volunteering Prevention System (TOPS) database.

Table 2: Temporary Exclusion Criteria at time of bacterial challenge

**Temporary Exclusion Criteria at Challenge**
Positive lateral flow test for SARS-CoV-2
Significant acute or acute-on-chronic infection within the previous 7 days or have experienced fever (>37.5°C) or subjective febrile symptoms within the previous 3 days
History of any antibiotic therapy during the previous 5 days
Any systemic corticosteroid (or equivalent) treatment in the previous 14 days, or for more than seven consecutive days within the past 3 months
Therapy with antacids, proton pump inhibitors or H2-receptor antagonists within 24 hours prior to challenge
Detection of gastrointestinal pathogens (other than *Salmonella*) in stool culture/PCR collected at the pre-challenge assessment (Day-7) including *Shigella* spp., *Campylobacter* spp., *E. Coli* O157, *Giardia* spp and *Cryptosporidium* spp, until two subsequent samples are negative. Detection of *Salmonella* spp. in stool prior to challenge (Day – 7) will result in exclusion from the study
Detection of extended spectrum beta lactamase producing organisms (ESBLs) or Carbapenem resistant organisms (CRO) until two subsequent samples are negative.

For peer review only

Table 3: Clinical sample collection plan for patients undergoing primary challenge. Values correspond to volume of blood collected in millilitres.

	Pre-challenge		Challenge period																				Follow up			
			Inpatient quarantine								Outpatient assessments							Post diagnosis/treatment								
	Screening	Day -7	Day 0	Day 1	Day 2	Day 3	Day 4	Day 5	Day 6	Day 7	Day 8	Day 9	Day 10	Day 11	Day 12	Day 13	Day 14	SD	SD +24h	SD +48h	SD +72h	SD +96h	Day 28	Day 90	Day 180	Day 365
Stool sample		x	x	x	x	x	x	x	x	x	x	x	x	x	x	x	x	x	x	x	x	x	x	x	x	x
Blood culture BACTEC			10	10	10	10	10	10	10	10	10	10	10	10	10	10	10	10	10	10	10	10				
FBC	1	1	1		1		1		1		1		1		1		1	1	1	1	1	1	1	1	1	
U&E/CRP/LFT	2	2	2		2		2		2		2		2		2		2	2	2	2	2	2	2	2	2	
Additional screening investigations*	10																									
Bacterial quantification																		10								
Serum sample			10							5							5	10					10	10	10	
Plasma Sample			3							3							3	3					3			
PBMC			25				10			25							25	25					25	25	25	25
Functional genomics			3	3	3	3				3								3					3	3		
DNA sample (including epigenetics)			x																				x		x	
Saliva sample			x														x						x		x	

*HIV/HBV/HV serology, HLA-B27, Haemoglobinopathy screen, Coeliac serology, HbA1c

Table 4: Simulation results comparing CRM with rule-based model.

Operating characteristics for CRM-based approach under 5 following scenarios:

Scenario 1 target dose at dose 1 with attack rate: 0.675, 0.8, 0.9, 0.95.
Scenario 2 target dose at dose 2 with attack rate: 0.5, 0.675, 0.8, 0.9.
Scenario 3 target dose at dose 4 with attack rate: 0.3, 0.4, 0.5, 0.675.
Scenario 4 target dose beyond dose 4 with attack rate: 0.2, 0.3, 0.4, 0.5.
Scenario 5 target dose under dose 1 with attack rate: 0.8, 0.9, 0.95, 0.975.

	**CRM-based***	**Rule-based**
**Scenario 1**		
Median sample size (IQR)	20 (20-25)	20 (20-25)
Probability of choosing the dose		
First dose too high	17.2%	16.4%
1 (67.5%)	69.1%	58.4%
2 (80%)	13.7%	8.6%
3 (90%)		
4 (95%)		
Fail to find a dose		16.6%
**Scenario 2**		
Median sample size (IQR)	30 (25-35)	30 (25-35)
Probability of choosing the dose		
First dose too high	0.7%	0.8%
1 (50%)	20.6%	17.3%
2 (67.5%)	64.9%	49.4%
3 (80%)	13.7%	3.9%
4 (90%)	0.1%	
Failed to find a dose	N/A	28.6%
**Scenario 3**		
Median sample size	35 (35-40)	30 (20-40)
Probability of choosing the dose		
1 (30%)	0.02%	0.6%
2 (40%)	2.2%	4.4%
3 (50%)	34.1%	14.1%
4 (67.5%)	59.1%	11.0%
Last dose too low	4.4%	0%
Fail to find a dose	N/A	69.9%
**Scenario 4**		
Median sample size	35 (35-40)	30 (25-35)
Probability of choosing the dose		
1 (20%)	0%	0%
2 (30%)	0.1%	0.8%
3 (40%)	5.3%	3.1%
4 (50%)	46.0%	10.7%
Last dose too low	48.6%	

For peer review only

Fail to find a dose	N/A	85.4%
**Scenario 5**		
Median sample size	20 (20-20)	20 (20-20)
Probability of choosing the dose		
First dose too high	64.7%	63.2%
1 (80%)	35.0%	32.3%
2 (90%)	0.3%	0.1%
3 (95%)	0%	0%
4 (97.5%)	0%	0%
Fail to find a dose	N/A	4.4%

*Parameters for the CRM-based design:

- Prior attack rate at the four doses: 0.6, 0.75, 0.85, 0.9;
- The attack rate is 67.5%;
- Starting at dose 1

For peer review only

Figure 1: Antibiotic Treatment Algorithm

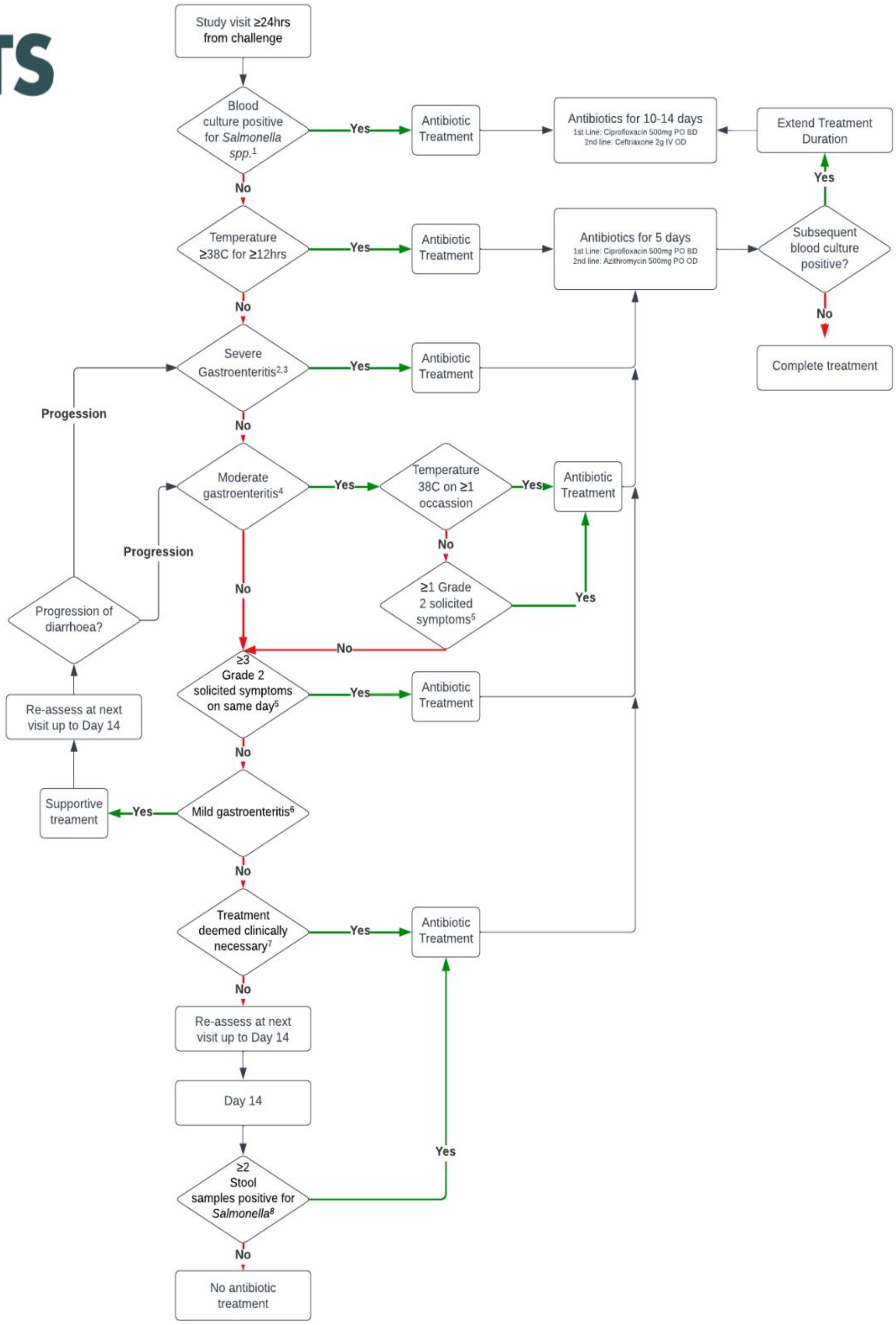

Notes

1 - Treatment initiated upon identification of Gram negative rods in blood culture, prior to formal speciation.

2- Loose/liquid stools defined as Type 6 or 7 stools on the Bristol stool chart.

3 - Severe gastroenteritis defined over a rolling 24-hour period as:
≥6 loose/liquid stools **and/or**
>800 g of loose/liquid stools **and/or**
≥2 stools with gross blood

4 - Moderate Gastroenteritis defined over a rolling 24-hour period as:
4-5 loose/liquid stools **and/or**
400-800g loose/liquid stools

5 - Solicited symptoms defined as:
Headache
Fatigue/Malaise
Anorexia
Abdominal pain
Nausea
Vomiting
Myalgia
Arthralgia
Cough

6 - Mild Gastroenteritis defined over a rolling 24-hour period as:
3 loose/liquid stools **and/or**
200-399g loose/liquid stools

7 - At the discretion of the principal investigator in consultation with the trial steering committee

8 - As detected by culture and/or PCR

Figure 2: Quarantine Discharge Algorithm

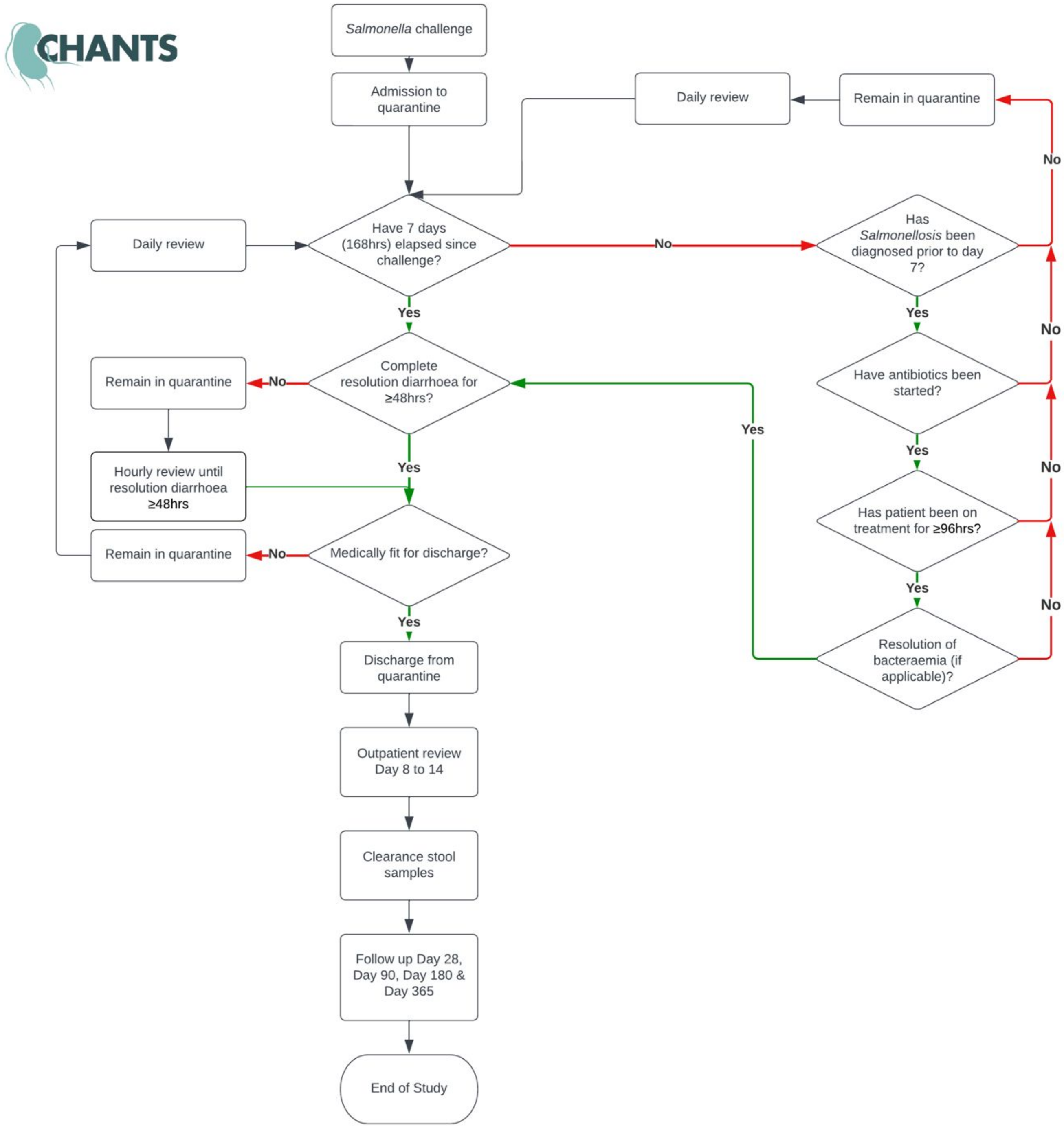

STANDARD PROTOCOL ITEMS: RECOMMENDATIONS FOR INTERVENTIONAL TRIALS

SPIRIT 2013 Checklist: Recommended items to address in a clinical trial protocol and related documents*

Section/item	Item No.	Description	Addressed on Page
**Administrative information**			
Title	1	Descriptive title identifying the study design, population, interventions, and, if applicable, trial acronym	1
Trial registration	2a	Trial identifier and registry name. If not yet registered, name of intended registry	2
	2b	All items from the World Health Organization Trial Registration Data Set	2
Protocol version	3	Date and version identifier	1
Funding	4	Sources and types of financial, material, and other support	17
Roles and responsibilities	5a	Names, affiliations, and roles of protocol contributors	1
	5b	Name and contact information for the trial sponsor	1
	5c	Role of study sponsor and funders, if any, in study design; collection, management, analysis, and interpretation of data; writing of the report; and the decision to submit the report for publication, including whether they will have ultimate authority over any of these activities	17

For peer review only

	5d	Composition, roles, and responsibilities of the coordinating centre, steering committee, endpoint adjudication committee, data management team, and other individuals or groups overseeing the trial, if applicable (see Item 21a for data monitoring committee)	8, 11
**Introduction**			
Background and rationale	6a	Description of research question and justification for undertaking the trial, including summary of relevant studies (published and unpublished) examining benefits and harms for each intervention	4-5
	6b	Explanation for choice of comparators	5, 7
Objectives	7	Specific objectives or hypotheses	6, 13
Trial design	8	Description of trial design including type of trial (eg, parallel group, crossover, factorial, single group), allocation ratio, and framework (eg, superiority, equivalence, noninferiority, exploratory)	5-9
**Methods: Participants, interventions, and outcomes**			
Study setting	9	Description of study settings (eg, community clinic, academic hospital) and list of countries where data will be collected. Reference to where list of study sites can be obtained	5

For peer review only

Eligibility criteria	10	Inclusion and exclusion criteria for participants. If applicable, eligibility criteria for study centres and individuals who will perform the interventions (eg, surgeons, psychotherapists)	6-7 (and supplementary table 1)
Interventions	11a	Interventions for each group with sufficient detail to allow replication, including how and when they will be administered	8-9
	11b	Criteria for discontinuing or modifying allocated interventions for a given trial participant (eg, drug dose change in response to harms, participant request, or improving/worsening disease)	N/A
	11c	Strategies to improve adherence to intervention protocols, and any procedures for monitoring adherence (eg, drug tablet return, laboratory tests)	N/A
	11d	Relevant concomitant care and interventions that are permitted or prohibited during the trial	9-10
Outcomes	12	Primary, secondary, and other outcomes, including the specific measurement variable (eg, systolic blood pressure), analysis metric (eg, change from baseline, final value, time to event), method of aggregation (eg, median, proportion), and time point for each outcome. Explanation of the clinical relevance of chosen efficacy and harm outcomes is strongly recommended	6 (and table 1)
Participant timeline	13	Time schedule of enrolment, interventions (including any run-ins and washouts), assessments, and visits for participants. A schematic diagram is highly recommended (see Figure)	Figure 1
Sample size	14	Estimated number of participants needed to achieve study objectives and how it was determined, including clinical and statistical assumptions supporting any sample size calculations	11-12
Recruitment	15	Strategies for achieving adequate participant enrolment to reach target sample size	6-7

For peer review only - http://bmjopen.bmj.com/site/about/guidelines.xhtml

Methods: Assignment of interventions (for controlled trials)

Allocation:

Sequence generation	16a	Method of generating the allocation sequence (eg, computer-generated random numbers), and list of any factors for stratification. To reduce predictability of a random sequence, details of any planned restriction (eg, blocking) should be provided in a separate document that is unavailable to those who enrol participants or assign interventions	8
Allocation concealment mechanism	16b	Mechanism of implementing the allocation sequence (eg, central telephone; sequentially numbered, opaque, sealed envelopes), describing any steps to conceal the sequence until interventions are assigned	8
Implementation	16c	Who will generate the allocation sequence, who will enrol participants, and who will assign participants to interventions	8
Blinding (masking)	17a	Who will be blinded after assignment to interventions (eg, trial participants, care providers, outcome assessors, data analysts), and how	8
	17b	If blinded, circumstances under which unblinding is permissible, and procedure for revealing a participant's allocated intervention during the trial	8

Methods: Data collection, management, and analysis

Data collection methods	18a	Plans for assessment and collection of outcome, baseline, and other trial data, including any related processes to promote data quality (eg, duplicate measurements, training of assessors) and a description of study instruments (eg, questionnaires, laboratory tests) along with their reliability and validity, if known. Reference to where data collection forms can be found, if not in the protocol	Table 1, Figure 1

For peer review only - http://bmjopen.bmj.com/site/about/guidelines.xhtml

	18b	Plans to promote participant retention and complete follow-up, including list of any outcome data to be collected for participants who discontinue or deviate from intervention protocols	Figure 1
Data management	19	Plans for data entry, coding, security, and storage, including any related processes to promote data quality (eg, double data entry; range checks for data values). Reference to where details of data management procedures can be found, if not in the protocol	11
Statistical methods	20a	Statistical methods for analysing primary and secondary outcomes. Reference to where other details of the statistical analysis plan can be found, if not in the protocol	11-12, Figure 2
	20b	Methods for any additional analyses (eg, subgroup and adjusted analyses)	Table 1
	20c	Definition of analysis population relating to protocol non-adherence (eg, as randomised analysis), and any statistical methods to handle missing data (eg, multiple imputation)	N/A
**Methods: Monitoring**			
Data monitoring	21a	Composition of data monitoring committee (DMC); summary of its role and reporting structure; statement of whether it is independent from the sponsor and competing interests; and reference to where further details about its charter can be found, if not in the protocol. Alternatively, an explanation of why a DMC is not needed	11
	21b	Description of any interim analyses and stopping guidelines, including who will have access to these interim results and make the final decision to terminate the trial	11

Harms	22	Plans for collecting, assessing, reporting, and managing solicited and spontaneously reported adverse events and other unintended effects of trial interventions or trial conduct	11
Auditing	23	Frequency and procedures for auditing trial conduct, if any, and whether the process will be independent from investigators and the sponsor	8, 11
**Ethics and dissemination**			
Research ethics approval	24	Plans for seeking research ethics committee/institutional review board (REC/IRB) approval	12
Protocol amendments	25	Plans for communicating important protocol modifications (eg, changes to eligibility criteria, outcomes, analyses) to relevant parties (eg, investigators, REC/IRBs, trial participants, trial registries, journals, regulators)	12
Consent or assent	26a	Who will obtain informed consent or assent from potential trial participants or authorised surrogates, and how (see Item 32)	6-7
	26b	Additional consent provisions for collection and use of participant data and biological specimens in ancillary studies, if applicable	N/A
Confidentiality	27	How personal information about potential and enrolled participants will be collected, shared, and maintained in order to protect confidentiality before, during, and after the trial	11
Declaration of interests	28	Financial and other competing interests for principal investigators for the overall trial and each study site	17
Access to data	29	Statement of who will have access to the final trial dataset, and disclosure of contractual agreements that limit such access for investigators	N/A

For peer review only

For peer review only - http://bmjopen.bmj.com/site/about/guidelines.xhtml

Ancillary and post-trial care	30	Provisions, if any, for ancillary and post-trial care, and for compensation to those who suffer harm from trial participation	12
Dissemination policy	31a	Plans for investigators and sponsor to communicate trial results to participants, healthcare professionals, the public, and other relevant groups (eg, via publication, reporting in results databases, or other data sharing arrangements), including any publication restrictions	12
	31b	Authorship eligibility guidelines and any intended use of professional writers	17
	31c	Plans, if any, for granting public access to the full protocol, participant-level dataset, and statistical code	N/A
**Appendices**			
Informed consent materials	32	Model consent form and other related documentation given to participants and authorised surrogates	Supplementary
Biological specimens	33	Plans for collection, laboratory evaluation, and storage of biological specimens for genetic or molecular analysis in the current trial and for future use in ancillary studies, if applicable	Figure 1, Table 1

*It is strongly recommended that this checklist be read in conjunction with the SPIRIT 2013 Explanation & Elaboration for important clarification on the items. Amendments to the protocol should be tracked and dated. The SPIRIT checklist is copyrighted by the SPIRIT Group under the Creative Commons "Attribution-NonCommercial-NoDerivs 3.0 Unported" license.

For peer review only

BMJ Open

Protocol for the Challenge Nontyphoidal *Salmonella* (CHANTS) Study: a first-in-human, in-patient, double-blind, randomised, safety and dose-escalation controlled human infection model

Journal:	*BMJ Open*
Manuscript ID	bmjopen-2023-076477.R1
Article Type:	Protocol
Date Submitted by the Author:	21-Nov-2023
Complete List of Authors:	Smith, Christopher; Imperial College London, Department of Infectious Disease<br>Smith, Emma; Imperial College London, Department of Infectious Disease<br>Rydlova, Anna; Imperial College London, Department of Infectious Disease<br>Varro, Robert; Imperial College London, Department of Infectious Disease<br>Hinton, Jay; University of Liverpool, Institute of Infection, Veterinary and Ecological Sciences<br>Gordon, Melita; University of Liverpool, Institute of Infection, Veterinary and Ecological Sciences; Kamuzu University of Health Sciences, Malawi Liverpool Wellcome Trust Clinical Research Programme<br>Choy, Robert; PATH<br>Liu , Xinxue ; Oxford University, Oxford Vaccine Group, Department of Paediatrics ; NIHR Oxford Biomedical Research Centre<br>Pollard, Andrew; Oxford University, Oxford Vaccine Group, Department of Paediatrics; NIHR Oxford Biomedical Research Centre<br>Chiu, Christopher; Imperial College London, Department of Infectious Disease<br>Cooke, Graham; Imperial College London, Department of Infectious Disease<br>Gibani, Malick; Imperial College London, Department of Infectious Disease
**Primary Subject Heading**:	Infectious diseases
Secondary Subject Heading:	Immunology (including allergy), Global health
Keywords:	INFECTIOUS DISEASES, Tropical medicine < INFECTIOUS DISEASES, Neglected Diseases, Microbiology < NATURAL SCIENCE DISCIPLINES, Immunology < NATURAL SCIENCE DISCIPLINES

Protocol for the Challenge Nontyphoidal *Salmonella* (CHANTS) Study: a first-in-human, in-patient, double-blind, randomised, safety and dose-escalation controlled human infection model.

Authors

Christopher Smith[1], Emma Smith[1], Anna Rydlova[1], Robert Varro[1], Jay C.D. Hinton[2], Melita A. Gordon[2,3], Robert K.M. Choy[4], Xinxue Liu[5,6], Andrew J. Pollard[5,6], Christopher Chiu[1], Graham S. Cooke[1], Malick M. Gibani[1]

[1]Department of Infectious Disease, Imperial College London, UK
[2]Institute of Infection, Veterinary and Ecological Sciences, University of Liverpool, UK
[3]Malawi-Liverpool-Wellcome Trust Clinical Research Programme, Malawi
[4]PATH, Seattle, USA
[5]Oxford Vaccine Group, Department of Paediatrics, University of Oxford, UK
[6]NIHR Oxford Biomedical Research Centre, Oxford, UK

Corresponding Author

Malick M. Gibani, Department of Infectious Disease, Imperial College London, St Mary's Campus, Norfolk Place, London, W2 1PG: m.gibani@imperial.ac.uk.

Keywords

Salmonella; *Salmonella* Typhimurium; Non-Typhoidal *Salmonella*; Human Challenge Model; Controlled Human Infection Model; Vaccines.

Protocol Version: 1.2

Study Sponsor

Imperial College London is the main research Sponsor for this study. For further information regarding the sponsorship conditions, please contact the Head of Regulatory Compliance at:

Research Governance and Integrity Team
Imperial College London and Imperial College Healthcare NHS Trust
Room 215, Level 2, Medical School Building
Norfolk Place
London, W2 1PG
Tel: 0207 594 1862

For peer review only

For peer review only - http://bmjopen.bmj.com/site/about/guidelines.xhtml

1 ABSTRACT

Introduction

Invasive nontyphoidal *Salmonella* (iNTS) serovars are a major cause of community-acquired bloodstream infections in sub-Saharan Africa (SSA). In this setting, *Salmonella enterica* serovar Typhimurium accounts for two-thirds of infections and is associated with an estimated case-fatality rate of 15-20%. Several iNTS vaccine candidates are in early-stage assessment which – if found effective – would provide a valuable public health tool to reduce iNTS disease burden. The CHANTS study aims to develop a first-in-human *Salmonella* Typhimurium controlled human infection model (CHIM) which can act as a platform for future vaccine evaluation, in addition to providing novel insights into iNTS disease pathogenesis.

Methods and Analysis

This double-blind, safety and dose-escalation study will randomise 40-80 healthy UK participants aged 18-50 to receive oral challenge with one of two strains of *S.* Typhimurium belonging to the ST19 (strain 4/74) or ST313 (strain D23580) lineages. 4/74 is a global strain often associated with diarrhoeal illness predominantly in high-income settings, whilst D23580 is an archetypal strain representing invasive disease-causing isolates found in SSA. The primary objective is to determine the minimum infectious dose (CFU) required for 60-75% of participants to develop clinical or microbiological features of systemic salmonellosis. Secondary endpoints are to describe and compare the clinical, microbiological, and immunological responses following challenge. Dose escalation or de-escalation will be undertaken by continual-reassessment methodology and limited within pre-specified safety thresholds. Exploratory objectives are to describe mechanisms of iNTS virulence, identify putative immune correlates of protection, and describe host-pathogen interactions in response to infection.

Ethics and Dissemination

Ethical approval has been obtained from the NHS Health Research Authority (London – Fulham Research Ethics Committee 21/PR/0051; IRAS Project ID 301659). The study findings will be disseminated in international peer-reviewed journals and presented at national/international stakeholder meetings. Study outcome summaries will be provided to both funders and participants.

Trial Registration

Clinicaltrials.gov (NCT05870150).

For peer review only - http://bmjopen.bmj.com/site/about/guidelines.xhtml

2 STRENGTHS AND LIMITATIONS

1. This first-in-human NTS CHIM utilises well characterised *S.* Typhimurium strains (ST19 and ST313) which are representative of currently circulating isolates accountable for both diarrhoeal and invasive disease phenotypes.
2. A double-blind, randomised study design permits objective evaluation of differential clinical phenotypes induced by bacterial challenge.
3. The continual reassessment method for dose-finding, compared with rule-based design, improves trial efficiency, and increases the probability of finding the correct challenge dose.
4. This study is limited by recruitment of healthy UK volunteers, whose responses to challenge will differ from children and immunocompromised adults most commonly affected by iNTS in sub-Saharan Africa.

For peer review only

For peer review only - http://bmjopen.bmj.com/site/about/guidelines.xhtml

3 INTRODUCTION

Nontyphoidal *Salmonella* (NTS) infections are most commonly associated with a self-limiting diarrhoeal illness (dNTS) [1]. In contrast, invasive nontyphoidal *Salmonella* disease (iNTS) typically presents as a non-specific febrile illness – often without diarrhoea – following invasion into sterile sites causing bloodstream infection (BSI), meningitis, or sepsis [2]. iNTS is particularly common in sub-Saharan Africa (SSA) where most recent estimates reported 535,000 cases, 77,500 deaths, and 4·26 million lost DALYs in 2017 [3]. Case fatality rates are estimated to range between 15-20%. The high case fatality reflects that invasive disease occurs almost exclusively in individuals with impaired immunity. Recognised host risk factors include malnutrition, malaria, and chronic anaemia in children, and HIV infection and malignancy in adults [2,4]. Effective treatment is also increasingly challenging; highlighted by the WHO's inclusion of *Salmonella enterica* as one of the priority pathogens posing the greatest risk to human health through expanding antimicrobial resistance (AMR) [5].

iNTS serovars isolated in community-acquired BSI exhibit geographic variation. In Africa, *Salmonella enterica* subspecies *enterica* serovars Typhimurium and Enteritidis (henceforth *S.* Typhimurium and *S.* Enteritidis) are responsible for >90% of all iNTS BSI, with *S.* Typhimurium accounting for two thirds of this burden [6–8]. Sequencing of isolates from across Africa has identified *S.* Typhimurium multilocus sequence type (ST) 313 as the major cause of invasive disease, which is distinct from sequence types 19 and 34 that are more frequently associated with diarrhoeal disease (dNTS) in high-income settings such as the UK. ST313 has been shown to have undergone sequential evolutionary changes that converge towards a genotype associated with invasive disease [9].

To date, there are no licensed iNTS vaccines available for use in humans. Development of effective vaccines may contribute to reduced case numbers, reduced antibiotic use, and prevention of antimicrobial resistance [10]. Several candidate vaccines are under evaluation, including in bivalent or multi-valent formulations which could target both typhoidal and nontyphoidal *Salmonella* serovars [11]. Vaccine development for iNTS is hampered – in part – by an incomplete understanding of mechanisms and determinants of immunity during natural infection. Investigation into these mechanisms could support iNTS vaccine development programmes through identification of immune correlates of protection.

Controlled human infection models (CHIM) have a strong track record in accelerating vaccine development, particularly for enteric pathogens [12,13]. For example, the live attenuated cholera

For peer review only - http://bmjopen.bmj.com/site/about/guidelines.xhtml

vaccine CVD 103-HgR was licensed as a travel vaccine on the basis of safety, immunogenicity and efficacy data arising from CHIM studies [14]. More recently, a programme of typhoidal *Salmonella* CHIM studies demonstrated efficacy of a Vi-tetanus toxoid (Vi-TT) conjugate vaccine against *S.* Typhi (Typbar-TCV, Bharat Biotech) [15]. These data contributed to recommendations from WHO SAGE to support programmatic use of Vi-TT in high burden settings and were supported by subsequent phase III trials demonstrating similar efficacy in Nepal, Bangladesh, and Malawi [16–18].

The CHANTS study has been funded by the Wellcome Trust to establish a first-in-human NTS CHIM, with the aim of addressing key knowledge gaps in dNTS and iNTS disease pathogenesis and immunity. We will conduct a double-blind, randomised, safety and dose-escalation study with the primary objective of determining the infectious dose required for 60-75% of participants to develop systemic salmonellosis. Successfully establishing a reproducible NTS CHIM could provide valuable insights into mechanisms of protective immunity and serve as a platform for future evaluation of candidate iNTS vaccines and therapeutics.

4 METHODS AND ANALYSIS

4.1 Study design

The CHANTS study is a double-blinded, randomised, quarantine-based, controlled human infection model which aims to establish the infectious dose required for healthy participants to develop salmonellosis following oral challenge with either *S.* Typhimurium ST313 (strain D23580) or ST19 (strain 4/74). A predicted total of 40-80 healthy adult volunteers who are eligible, and consenting, will be randomised 1:1 to receive oral challenge with either D23580 or 4/74. The study will be conducted at Imperial College London with clinical support from partner tertiary hospitals at Imperial College Healthcare NHS Trust. Participant screening and recruitment commenced in August 2023, and the study is anticipated to conclude in Summer 2025.

4.2 Patient and public involvement

Patient and public engagement activities have been conducted throughout the protocol design stage, gaining valuable insights from the involvement of key stakeholders. Focus groups have been conducted with support of the NIHR Imperial Biomedical Research Centre patient experience research

For peer review only - http://bmjopen.bmj.com/site/about/guidelines.xhtml

group. Discussions with members of the public in this forum – including past participants of previous CHIMs – have identified key considerations for the safe and acceptable conduct of this trial and supported the development of clear and thorough participant-facing information to underpin informed consent. Future similar activities are planned in regions with high iNTS burden to establish acceptability of potential future transfer of an iNTS CHIM to settings most affected by the disease under investigation.

4.3 Study objectives and outcomes

The primary objective is to determine the minimum infectious dose in colony forming units (CFU) required for 60-75% of healthy participants to develop clinical or microbiological features of systemic salmonellosis following oral challenge with either *S.* Typhimurium D23580 or 4/74. Systemic salmonellosis is defined as the development of either: (1) fever ≥38°C on ≥2 occasions ≥12 hours apart, or (2) *S.* Typhimurium bacteraemia at any time point. As outlined above, exposure to *S.* Typhimurium can result in heterogeneous disease phenotypes, ranging from asymptomatic gastrointestinal colonisation to bloodstream infection. Secondary endpoints include determining the proportion of participants who develop outcomes along this disease spectrum, including *Salmonella* colonisation, diarrhoeal disease (dNTS), systemic symptoms, or severe salmonellosis. Secondary study outcomes also aim to describe the immune response to *S.* Typhimurium infection (Table 1: Primary and secondary study objectives and outcomes).

In this first-in-human study, we have a unique opportunity to elucidate mechanisms of bacterial virulence in dNTS and iNTS, identify immune correlates of protection, describe host-pathogen interactions, changes in host microbiota in response to infection, and develop novel diagnostic platforms (Supplementary Table 1).

4.4 Recruitment and eligibility

Several strategies will be employed to recruit study participants, including advertising throughout local hospitals, GP surgeries, and higher education institutions; media and website advertising; direct email to members of the NIHR Imperial Clinical Research Facility healthy volunteer database; direct mail-out and email to potentially eligible participants identified via National Health Applications and Infrastructure Services (NHAIS). Consenting healthy adult males and non-pregnant females aged 18-50 without pre-existing risk factors for severe iNTS or chronic infection will be recruited. Strict

For peer review only - http://bmjopen.bmj.com/site/about/guidelines.xhtml

For peer review only

eligibility criteria have been designed to mitigate risks of severe disease, chronic carriage, or development of focal seeded infection. Detailed screening will include comprehensive medical history, physical examination, baseline blood counts, biochemical profiles, screening for immunosuppression, and abdominal ultrasonography to exclude subclinical biliary or abdominal aortic disease (see supplementary Table 2 for full eligibility criteria). Individuals who have had documented prior *Salmonella* infection, or received live-attenuated oral Ty21a typhoid vaccine, will be excluded due to potential cross-protective immune responses against *S*. Typhimurium. Further temporary exclusion criteria will be reviewed prior to oral challenge to exclude intercurrent infection or antimicrobial use which may bias results (Supplementary Table 3).

4.5 Study interventions

Consenting participants who fulfil eligibility criteria will enter the study at Day -7 (7 days prior to oral bacterial challenge). Days 0-7 will take place under clinical observation in a quarantine research facility at Imperial College Healthcare NHS Trust, with subsequent daily out-patient reviews on Days 8-14, prior to long-term follow-up visits at Days 28, 90, 180, and 365. The overall participant journey is illustrated in Figure 1.

4.5.1 Challenge Strain Selection and Manufacture

Challenge strains have been selected to maximise future utility of the model in supporting iNTS vaccine development. Wild-type ST313 (D23580) and ST19 (4/74) are phylogenetically representative of contemporary disease-causing isolates, have a traceable history from isolation, and have undergone detailed genotypic, phenotypic, and transcriptomic characterisation [19,20]. Bacterial stocks have been manufactured to GMP standard by the Walter Reed Army Institute of Research, MD, USA, in collaboration with PATH (Seattle, USA), with strains originally drawn from the UKHSA National Collection of Type Cultures.

ST313 lineage 2 reference strain D23580 was originally isolated from a blood culture obtained from a 24-month-old HIV-negative child at Queen Elizabeth Central Hospital, Blantyre, Malawi, and is considered an archetypal isolate representative of strains causing iNTS in SSA. ST19 (4/74), originally isolated from a calf in England in 1974, is considered an archetypal strain causative of dNTS and representative of current clinical infections. D23580 harbours a distinct prophage and plasmid

For peer review only - http://bmjopen.bmj.com/site/about/guidelines.xhtml

repertoire compared with 4/74, including a virulence plasmid pSLT encoding resistance to ampicillin, chloramphenicol, streptomycin, sulphonamides, and trimethoprim [20]. Both strains are susceptible to ciprofloxacin, azithromycin and third generation cephalosporins, ensuring a range of treatment options are available for challenged participants. The rationale for strains selected for use in this model, and their relative merits, have been discussed in detail elsewhere [21].

4.5.2 Randomisation and Blinding

Block randomisation (block size = 10) to strain allocation will be carried out on or after Day -7. An initial sentinel cohort (n = 10) of will be randomised 1:1 (block sizes = 2, 4, 4) to receive a starting dose 1-5 x 10^3 CFU of either strain 4/74 or D23580. This starting dose has been determined based on prior experience from typhoidal CHIMs and continual reassessment method (CRM) dose prediction simulations (section 4.8) [15,22–24]. Unblinded attack rates and safety data will be reviewed by the Data Safety and Monitoring Committee (DSMC) prior to further participant randomisation (section 4.6). Randomisation lists will be generated and stored on a secure server accessible only by unblinded study team members. All clinical staff and participants will remain blinded to challenge allocation for the duration of the study.

4.5.3 Dose Escalation/De-escalation

Dose escalation and de-escalation decisions will be undertaken by the continual reassessment method (CRM). Escalation for both strains will occur in parallel, starting at 1-5 x 10^3 CFU, increasing to a maximum of 1-5 x 10^6 CFU or decreasing down to 1-5 x 10^1 CFU. Participants will be randomised to receive one of the two challenge agents at the dose determined by the CRM until target dosing is established for one agent. From that point, the remaining participants will be challenged with the other agent. We aim to recruit between 40-80 participants in total (20-40 for each challenge agent). The final challenge dose will be defined as one that achieves a 60-75% attack rate. The DSMC will be provided with an updated report after each cohort of 5 completes challenge. The dose escalation/de-escalation decision algorithm is illustrated in Figure 2.

4.5.4 Challenge procedures

Participants will receive oral bacterial challenge with either D23580 or 4/74 on study Day 0, following admission to an isolation side-room within the quarantine facility. Assessments will be conducted to

For peer review only - http://bmjopen.bmj.com/site/about/guidelines.xhtml

confirm the absence of any temporary exclusion criteria (see supplementary Table 3) prior to challenge. Female participants will also undergo pregnancy testing both at screening and immediately prior to challenge. Both *S.* Typhimurium challenge agents used in this study will be administered via the oral route with sodium bicarbonate buffer to achieve gastric acid neutralisation. Participants will fast for 90 minutes before and after challenge. The solution for ingestion containing either wild type S. Typhimurium 4/74 or D23580 will be prepared in a Class II biological safety cabinet that is solely used for the purpose of preparing the challenge solution.

4.5.5 Clinical Assessment During Quarantine

Participants will be reviewed daily during admission to the quarantine facility by a clinical member of the study team. Extensive assessments will be undertaken, including physical examination, recording vital signs, assessment of mood, and review of participant symptom diaries. Blood, stool, and saliva specimen collection will be undertaken according to timelines outlined in supplementary table 4. Supportive therapy will be provided as required for symptom control e.g., oral rehydration solution or intravenous fluids, analgesia, and anti-emetics.

4.5.6 Infection Prevention and Control

Participants will be managed using infection control protocols appropriate for enteric infections. Study investigators will adhere to strict hand-hygiene policies of the research facility and utilise enteric personal protective equipment (PPE) for all study procedures. Participants will receive clear instruction on hand hygiene procedures – including illustrated guidance – to be adhered to during and after quarantine, in addition to guidance on cleaning protocols for bathrooms in the home environment following discharge. Participants will be nursed within single-occupancy side-rooms with ensuite toilet facilities for the duration of quarantine. The near-patient environment, including door handles, taps, toilet flushes and bed rails will be decontaminated daily throughout the quarantine period in accordance with local policies. Assessment of *Salmonella* environmental contamination within the treatment facility will be undertaken as an exploratory study objective, utilising culture, and molecular methods to detect the presence of *S.* Typhimurium in the participant's immediate surroundings and in high traffic clinical areas.

Close contacts of participants, including household contacts, will be offered the opportunity to be screened for *Salmonella* infection, which will involve obtaining three stool samples 48-hours apart a

For peer review only - http://bmjopen.bmj.com/site/about/guidelines.xhtml

minimum of seven days after the participant has begun antibiotic treatment. If either stool culture of a household contact is positive, he/she will be referred to a Consultant in Infectious Diseases for appropriate antibiotic management and the local health protection unit will be informed.

For peer review only

For peer review only - http://bmjopen.bmj.com/site/about/guidelines.xhtml

4.5.7 Antimicrobial therapy

Treatment of *Salmonella* infection in healthy immunocompetent adults poses a number of specific challenges. Antimicrobial therapy is indicated for invasive disease but may prolong shedding in patients with pauci-symptomatic gastroenteritis. We have elected to offer rescue treatment in specific circumstances. The rationale for this decision is discussed elsewhere [21]. Antibiotic therapy will be initiated in all participants who fulfil criteria for *Salmonella* diagnosis (SD) through reaching either or both of the defined primary endpoints of persistent fever or bloodstream infection. Treatment will also be commenced in participants who do not achieve the primary outcome measures but reach any of the other pre-specified treatment criteria outlined in Table 2. Treatment for bloodstream infection will comprise oral ciprofloxacin 500mg twice daily for 10-14 days, or second line therapy of intravenous ceftriaxone 1g once daily for up to 14 days. All other treatment indications (systemic illness, gastroenteritis, asymptomatic/pauci-symptomatic shedding) will be treated with oral ciprofloxacin 500mg twice daily for 5 days, or with second-line oral azithromycin 500mg once daily for 5 days. Antibiotic treatment will not be routinely offered to participants who do not fulfil any pre-specified treatment criteria. The full antibiotic treatment decision algorithm is illustrated in supplementary figure 1.

4.5.8 Discharge from Quarantine

The duration of quarantine will be 7 days from the time of oral challenge (168 hours). Participants will be eligible for discharge if they fulfil the criteria outlined in Table 3. Some participants may be discharged earlier than day 7 if they have been started on antibiotic therapy and completed 96 hours of follow-up after starting treatment. If these criteria are not met on day 7, then participants will be asked to remain in quarantine until discharge criteria are met. The discharge decision making algorithm is illustrated in supplementary figure 2.

Discharge criteria have been designed to maximise participant clinical safety in addition to minimising risk of secondary onward transmission. Current UKHSA guidance does not mandate the collection of clearance stool cultures following an episode of gastroenteritis secondary to NTS infection. Instead, the absence of diarrhoea for at least 48 hours is considered sufficient for release from isolation [25]. In this study, clearance stool cultures will be obtained to meet a secondary study objective of measuring predicted differences in patterns of shedding between 4/74 and D23580 strains.

For peer review only - http://bmjopen.bmj.com/site/about/guidelines.xhtml

4.6 Governance

An independent Data and Safety Monitoring Committee (DSMC) has been appointed to provide real-time oversight of safety and trial conduct. The DSMC will review unblinded safety data throughout the study according to a pre-defined DSMC charter. The committee will have access to data and, if required, will monitor these data, and make recommendations to the study investigators on whether there are any ethical or safety reasons the trial should not continue. The DSMC will review the attack rate and advise on dose escalation/de-escalation decisions. Study monitoring will also be undertaken by a Contract Research Organisation (CRO), separate to and independent from the DSMC, to ensure safety and compliance with the approved study protocol. Strict data management plans will be adhered to with compliance to Good Clinical Practice guidelines.

4.7 Safety

As with all controlled human infection models, maintenance of participant safety during and after the study is paramount. In this model, general risks associated with participation may include study-fatigue, repeated phlebotomy procedures, and consequences of isolation in quarantine. Risks more directly attributable to challenge agents include symptomatic infection, complications of antibiotic treatment (in the case of bacterial challenge), and the small risk of long-term sequelae. Potential risks have been given extensive consideration, with risk mitigation strategies devised to minimise possible impact on participants. The potential complications of participation – and risk mitigation strategies – have been discussed elsewhere [21]. In addition to participant safety, careful consideration has been given to ensure safety of study investigators and close contacts. Clear infection control protocols have been developed (as outlined above), in addition to close liaison with UKHSA to monitor and address any potential secondary transmission events, including within households following discharge from the quarantine facility.

4.8 Statistical analysis

This dose-finding study will use the continual reassessment method (CRM) to determine the target dose that can achieve a 60-75% attack rate [26,27]. To our knowledge, this will be the first dose-finding CHIM to employ CRM methodology. The advantages over traditional rule-based dose-finding studies are that the CRM method borrows information across all dose levels, which increases likelihood of

For peer review only

For peer review only - http://bmjopen.bmj.com/site/about/guidelines.xhtml

finding the true dose for a given sample size compared with rule-based methods in the majority of scenarios, particularly when the maximum sample size is small. There is no formal sample size calculation for this study. The maximum sample size will be 40 participants for each challenge strain. The operating characteristics of the CRM and the rule-based approach are illustrated in supplementary Table 5.

5 ETHICS AND DISSEMINATION

This study protocol has been reviewed and approved by the NHS Health Research Authority (London – Fulham Research Ethics Committee 21/PR/0051; IRAS 301659) and is registered on clinicaltrials.gov (registration number NCT05870150). The sponsor is Imperial College London, and the study is indemnified under institutional and NHS insurance policies. Our findings will be of significant international interest, with several potential future applications for assessment of iNTS vaccines and novel therapeutics. We will publish all findings in peer-reviewed journals, and ensure presentation at national and international conferences, in addition to close liaison with key stakeholders, participants and funders.

6 DISCUSSION

The global morbidity and mortality burden from iNTS disease remains unacceptably high, with the greatest impact concentrated in children and immunocompromised adults in sub-Saharan Africa. Effective vaccines are urgently required as a public health tool to avert cases and prevent expansion of antibiotic resistant strains. Vaccine development for iNTS is hampered – in part – by an incomplete understanding of mechanisms of protective immunity against natural infection. In addition, large-scale phase III trials for iNTS vaccines will require substantial time and financial commitments.

CHIMs offer a platform to support vaccine development, providing a means of obtaining early vaccine safety data and efficacy read-outs. As has been observed for other enteric pathogens – including typhoidal *Salmonella* serovars – demonstration of efficacy in the context of a CHIM can support vaccine licensure and aid selection of the most appropriate candidates for field testing, de-risking phase III studies. The success of typhoidal CHIM studies in demonstrating efficacy of the Vi-TT (Typbar TCV, Bharat Biotech) vaccine laid the groundwork for field trials in Asia and Africa. The CHANTS study aims to develop a safe and reproducible model of iNTS disease, identify immune correlates of

For peer review only - http://bmjopen.bmj.com/site/about/guidelines.xhtml

protection, elucidate mechanisms of bacterial virulence, and provide a platform for future iNTS vaccine randomised trials.

As a first-in-human model the CHANTS study provides a unique opportunity to improve our understanding of the pathobiology of infection with *S.* Typhimurium strains 4/74 and D23580. The randomised, double-blinded study design allows objective analysis of differing clinical phenotypes induced in healthy volunteers by two strains which cause significantly different disease phenotypes in field settings. We hypothesise that ST313 (D23580) will be associated with an invasive phenotype compared with the enterocolitis anticipated as a result of challenge with ST19 (4/74).

Longitudinal descriptions of humoral, cellular, and mucosal immune responses to infection will provide detailed insights into mechanisms of protective immunity. Identification of these immune correlates of protection can support and inform future iNTS vaccine design. Furthermore, gaining a deeper understanding of bacterial virulence mechanisms through transcriptomic analyses can support future vaccination and therapeutic strategies.

The primary limitations of the CHANTS study – shared with most controlled human infection models – stem from undertaking the study in a healthy adult volunteer group which may not accurately reflect natural disease acquisition and pathogenesis. There are clear host factor differences between this group and individuals most susceptible to iNTS disease. However, as with prior CHIMs undertaken in healthy participants, we anticipate this infection model will generate key insights into *Salmonella* infection biology with future translational applications. Successful development of an *S.* Typhimurium CHIM, with an established safety profile and infectious dose, could be transferred to an endemic setting for future investigation in a population more closely resembling those at greatest risk of disease, as has been achieved previously in a range of enteric and non-enteric pathogen challenge models [28–32].

In addition to inherent differences between our study population and those at greatest risk of iNTS, there are limitations in achievable study endpoints. Our primary endpoint of persistent fever, with or without bloodstream infection, does not necessarily reflect the severe clinical phenotypes an iNTS vaccine would aim to protect against in field settings. However, in view of the heterogeneous clinical phenotypes associated with NTS infection, ranging from asymptomatic colonisation through to septic shock, this endpoint is felt to represent the optimal trade-off between clinical manifestations of disease and patient safety within the context of a CHIM. The relative merits of endpoint selection and their rationale have been discussed previously [21].

For peer review only - http://bmjopen.bmj.com/site/about/guidelines.xhtml

In summary, the CHANTS study presents a valuable opportunity to improve our understanding of iNTS disease and support vaccine development efforts. Whilst there are limitations, insights gained from this first-in-human model can inform future iNTS vaccine design and provide a platform for future randomised trials. Successful development of an *S.* Typhimurium CHIM could lead to future investigations in populations more closely resembling those at greatest risk of disease.

For peer review only

For peer review only - http://bmjopen.bmj.com/site/about/guidelines.xhtml

7 REFERENCES

[1] Roth GA, Abate D, Abate KH, Abay SM, Abbafati C, Abbasi N, et al. Global, regional, and national age-sex-specific mortality for 282 causes of death in 195 countries and territories, 1980–2017: a systematic analysis for the Global Burden of Disease Study 2017. The Lancet 2018;392:1736–88. https://doi.org/10.1016/S0140-6736(18)32203-7.

[2] Feasey NA, Dougan G, Kingsley RA, Heyderman RS, Gordon MA. Invasive non-typhoidal salmonella disease: an emerging and neglected tropical disease in Africa. Lancet 2012;379:2489–99. https://doi.org/10.1016/S0140-6736(11)61752-2.

[3] GBD 2017 Non-Typhoidal Salmonella Invasive Disease Collaborators. The global burden of non-typhoidal salmonella invasive disease: a systematic analysis for the Global Burden of Disease Study 2017. Lancet Infect Dis 2019;19:1312–24. https://doi.org/10.1016/S1473-3099(19)30418-9.

[4] Feasey NA, Everett D, Faragher EB, Roca-Feltrer A, Kang'ombe A, Denis B, et al. Modelling the Contributions of Malaria, HIV, Malnutrition and Rainfall to the Decline in Paediatric Invasive Non-typhoidal Salmonella Disease in Malawi. PLoS Negl Trop Dis 2015;9:e0003979. https://doi.org/10.1371/journal.pntd.0003979.

[5] Tacconelli E, Carrara E, Savoldi A, Harbarth S, Mendelson M, Monnet DL, et al. Discovery, research, and development of new antibiotics: the WHO priority list of antibiotic-resistant bacteria and tuberculosis. Lancet Infect Dis 2018;18:318–27. https://doi.org/10.1016/S1473-3099(17)30753-3.

[6] Uche IV, MacLennan CA, Saul A. A Systematic Review of the Incidence, Risk Factors and Case Fatality Rates of Invasive Nontyphoidal Salmonella (iNTS) Disease in Africa (1966 to 2014). PLoS Negl Trop Dis 2017;11:e0005118. https://doi.org/10.1371/journal.pntd.0005118.

[7] Ao TT, Feasey NA, Gordon MA, Keddy KH, Angulo FJ, Crump JA. Global burden of invasive nontyphoidal Salmonella disease, 2010(1). Emerg Infect Dis 2015;21. https://doi.org/10.3201/eid2106.140999.

[8] Marchello CS, Fiorino F, Pettini E, Crump JA, Vacc-iNTS Consortium Collaborators. Incidence of non-typhoidal Salmonella invasive disease: A systematic review and meta-analysis. J Infect 2021;83:523–32. https://doi.org/10.1016/j.jinf.2021.06.029.

[9] Pulford CV, Perez-Sepulveda BM, Canals R, Bevington JA, Bengtsson RJ, Wenner N, et al. Stepwise evolution of Salmonella Typhimurium ST313 causing bloodstream infection in Africa. Nat Microbiol 2021;6:327–38. https://doi.org/10.1038/s41564-020-00836-1.

[10] Read the report : Vaccines for AMR n.d. https://vaccinesforamr.org/read-the-report/ (accessed April 27, 2023).

[11] Baliban SM, Lu Y-J, Malley R. Overview of the Nontyphoidal and Paratyphoidal Salmonella Vaccine Pipeline: Current Status and Future Prospects. Clin Infect Dis 2020;71:S151–4. https://doi.org/10.1093/cid/ciaa514.

[12] Choy RKM, Bourgeois AL, Ockenhouse CF, Walker RI, Sheets RL, Flores J. Controlled Human Infection Models To Accelerate Vaccine Development. Clin Microbiol Rev 2022;35:e0000821. https://doi.org/10.1128/cmr.00008-21.

[13] Roestenberg M, Hoogerwerf M-A, Ferreira DM, Mordmüller B, Yazdanbakhsh M. Experimental infection of human volunteers. Lancet Infect Dis 2018;18:e312–22. https://doi.org/10.1016/S1473-3099(18)30177-4.

[14] Chen WH, Cohen MB, Kirkpatrick BD, Brady RC, Galloway D, Gurwith M, et al. Single-dose Live Oral Cholera Vaccine CVD 103-HgR Protects Against Human Experimental Infection With Vibrio cholerae O1 El Tor. Clin Infect Dis 2016;62:1329–35. https://doi.org/10.1093/cid/ciw145.

[15] Jin C, Gibani MM, Moore M, Juel HB, Jones E, Meiring J, et al. Efficacy and immunogenicity of a Vi-tetanus toxoid conjugate vaccine in the prevention of typhoid fever using a controlled human

For peer review only

For peer review only - http://bmjopen.bmj.com/site/about/guidelines.xhtml

infection model of Salmonella Typhi: a randomised controlled, phase 2b trial. The Lancet 2017;390:2472–80. https://doi.org/10.1016/S0140-6736(17)32149-9.
[16] Shakya M, Colin-Jones R, Theiss-Nyland K, Voysey M, Pant D, Smith N, et al. Phase 3 Efficacy Analysis of a Typhoid Conjugate Vaccine Trial in Nepal. N Engl J Med 2019;381:2209–18. https://doi.org/10.1056/NEJMoa1905047.
[17] Patel PD, Patel P, Liang Y, Meiring JE, Misiri T, Mwakiseghile F, et al. Safety and Efficacy of a Typhoid Conjugate Vaccine in Malawian Children. N Engl J Med 2021;385:1104–15. https://doi.org/10.1056/NEJMoa2035916.
[18] Qadri F, Khanam F, Liu X, Theiss-Nyland K, Biswas PK, Bhuiyan AI, et al. Protection by vaccination of children against typhoid fever with a Vi-tetanus toxoid conjugate vaccine in urban Bangladesh: a cluster-randomised trial. The Lancet 2021;398:675–84. https://doi.org/10.1016/S0140-6736(21)01124-7.
[19] Kröger C, Colgan A, Srikumar S, Händler K, Sivasankaran SK, Hammarlöf DL, et al. An infection-relevant transcriptomic compendium for Salmonella enterica Serovar Typhimurium. Cell Host Microbe 2013;14:683–95. https://doi.org/10.1016/j.chom.2013.11.010.
[20] Canals R, Hammarlöf DL, Kröger C, Owen SV, Fong WY, Lacharme-Lora L, et al. Adding function to the genome of African Salmonella Typhimurium ST313 strain D23580. PLoS Biol 2019;17:e3000059. https://doi.org/10.1371/journal.pbio.3000059.
[21] Smith C, Smith E, Chiu C, Hinton J, Perez Sepulveda B, Gordon M, et al. The Challenge Non-Typhoidal Salmonella (CHANTS) Consortium: Development of a non-typhoidal Salmonella controlled human infection model: Report from a consultation group workshop, 05 July 2022, London, UK. Wellcome Open Res 2023;8:111. https://doi.org/10.12688/wellcomeopenres.19012.1.
[22] Gibani MM, Jin C, Shrestha S, Moore M, Norman L, Voysey M, et al. Homologous and heterologous re-challenge with Salmonella Typhi and Salmonella Paratyphi A in a randomised controlled human infection model. PLoS Negl Trop Dis 2020;14:e0008783. https://doi.org/10.1371/journal.pntd.0008783.
[23] Gibani MM, Jones E, Barton A, Jin C, Meek J, Camara S, et al. Investigation of the role of typhoid toxin in acute typhoid fever in a human challenge model. Nat Med 2019;25:1082–8. https://doi.org/10.1038/s41591-019-0505-4.
[24] Dobinson HC, Gibani MM, Jones C, Thomaides-Brears HB, Voysey M, Darton TC, et al. Evaluation of the Clinical and Microbiological Response to Salmonella Paratyphi A Infection in the First Paratyphoid Human Challenge Model. Clinical Infectious Diseases 2017;64:1066–73. https://doi.org/10.1093/cid/cix042.
[25] Recommendations for the Public Health Management of Gastrointestinal Infections 2019 2020.
[26] O'Quigley J, Pepe M, Fisher L. Continual reassessment method: a practical design for phase 1 clinical trials in cancer. Biometrics 1990;46:33–48.
[27] Wheeler GM, Mander AP, Bedding A, Brock K, Cornelius V, Grieve AP, et al. How to design a dose-finding study using the continual reassessment method. BMC Med Res Methodol 2019;19:18. https://doi.org/10.1186/s12874-018-0638-z.
[28] Morton B, Jambo K, Chikaonda T, Rylance J, Henrion MYR, Banda NP, et al. The influence of pneumococcal conjugate vaccine-13 on nasal colonisation in a controlled human infection model of pneumococcal carriage in Malawi: a double-blinded randomised controlled trial protocol. Wellcome Open Res 2021;6:240. https://doi.org/10.12688/wellcomeopenres.17172.2.
[29] Kapulu MC, Kimani D, Njuguna P, Hamaluba M, Otieno E, Kimathi R, et al. Controlled human malaria infection (CHMI) outcomes in Kenyan adults is associated with prior history of malaria exposure and anti-schizont antibody response. BMC Infect Dis 2022;22:86. https://doi.org/10.1186/s12879-022-07044-8.
[30] Pitisuttithum P, Cohen MB, Phonrat B, Suthisarnsuntorn U, Bussaratid V, Desakorn V, et al. A human volunteer challenge model using frozen bacteria of the new epidemic serotype, V.

For peer review only

For peer review only - http://bmjopen.bmj.com/site/about/guidelines.xhtml

cholerae O139 in Thai volunteers. Vaccine 2001;20:920–5. https://doi.org/10.1016/s0264-410x(01)00381-4.

[31] Pitisuttithum P, Islam D, Chamnanchanunt S, Ruamsap N, Khantapura P, Kaewkungwal J, et al. Clinical Trial of an Oral Live Shigella sonnei Vaccine Candidate, WRSS1, in Thai Adults. Clin Vaccine Immunol 2016;23:564–75. https://doi.org/10.1128/CVI.00665-15.

[32] Bodhidatta L, Pitisuttithum P, Chamnanchanant S, Chang KT, Islam D, Bussaratid V, et al. Establishment of a Shigella sonnei human challenge model in Thailand. Vaccine 2012;30:7040–5. https://doi.org/10.1016/j.vaccine.2012.09.061.

Authors Contributions

Conceptualisation and study design: CS, ES, AR, RV, JCDH, MAG, RKMC, XL, AJP, CC, GSC, MMG

Data analysis plan: CS, XL, GSC, MMG

Drafting of protocol: CS, ES, AR, RV, GSC, MMG

Protocol amendments: CS, RKMC, AJP, XL, MMG

Data Availability Statement

As this manuscript describes a study protocol, no datasets are available as yet. An ICMJE data availability statement is included in Supplementary Material Table 6 applicable after conclusion of the study.

Funding

The CHANTS study is funded by The Wellcome Trust (grant number 224029/Z/21/Z: awarded to principal investigator MMG). CS, GSC, CC and MMG are supported, in part, by the National Institute of Health Research Imperial Biomedical Research Centre.

Disclaimer

The funder of the study has had no role in study design, and will have no role in data collection, data analysis, data interpretation, writing of the report or decision to submit.

Competing Interests

None declared.

Word Count

Main Text: 3698

For peer review only - http://bmjopen.bmj.com/site/about/guidelines.xhtml

Table 1: Primary and secondary study objectives and outcomes

	**Objective(s)**	**Outcome(s)/Endpoint(s)**
**Primary**	Determine the minimum infectious dose (CFU) of *S.* Typhimurium required for 60-75% of volunteers to develop systemic Salmonellosis following oral challenge.	The proportion of participants who develop:<br>1. Fever ≥38°C on ≥2 occasions ≥12 hours apart **and/or**<br>2. *Salmonella* Typhimurium bacteraemia at any time point
**Secondary**	Describe colonisation rates following oral challenge at different doses.	The proportion from whom *Salmonella* Typhimurium is isolated from stool on ≥2 occasions ≥48 hours from challenge at different doses of each strain.
	Describe gastroenteritis rates following oral challenge at different doses.	The proportion of participants at different doses of each strain developing:<br>1.1 Severe diarrhoea* **and/or**<br>1.2 Moderate diarrhoea** **plus** Fever ≥38°C on ≥1 occasion **and/or** ≥1 Grade 2 gastrointestinal symptoms (abdominal pain, nausea, vomiting, tenesmus)
	Determine persistent fever rates following oral challenge at different doses.	The proportion of participants who develop fever ≥38°C on ≥2 occasions ≥12 hours apart at different doses of each strain.
	Describe bacteraemia rate following oral challenge at different doses.	The proportion of participants developing *Salmonella* Typhimurium bacteraemia at any timepoint following challenge at different doses of each strain.
	Describe the rates of systemic *Salmonellosis* according to **an alternative composite diagnostic criterion** following oral challenge at different doses.	The proportion of participants meeting the criteria for a composite diagnosis of *Salmonellosis* at different doses of each strain defined as any of:<br>1 *S.* Typhimurium isolated from stool on ≥2 occasions ≥48 hours from challenge **and/or**<br>2 Gastroenteritis **and/or**<br>3 Fever ≥38°C on ≥2 occasions ≥12 hours apart **and/or**<br>4 *S.* Typhimurium bacteraemia
	Describe the safety of oral challenge with *S.* Typhimurium 4/74 and D23580 strains.	The proportion of participants at different doses of each strain reporting: adverse events, adverse events of special interest, SAEs, SUSARs, concomitant medication usage.
	Compare **clinical** features following oral challenge with *S.* Typhimurium strains 4/74 or D23580.	A comparison of the clinical features of *Salmonella* infection after challenge, with specific reference to the proportion in each group developing:<br>1 Diarrhoea (volume, frequency, consistency, gastrointestinal symptom severity score, time to onset)<br>2 Any fever ≥38°C (including fever clearance time)<br>3 Solicited symptoms at any time: headache, malaise, anorexia, abdominal pain, nausea, vomiting, dysentery, myalgia, arthralgia, cough, rash (including measurement of severity, and duration from onset to resolution)

For peer review only - http://bmjopen.bmj.com/site/about/guidelines.xhtml

	Compare **microbiological** features of **gastrointestinal infection** following oral challenge with *S.* Typhimurium 4/74 or D23580.	Gastrointestinal *Salmonella* infection after challenge with 4/74 or D23580 strains, with specific reference to:<br>1 Time to onset in days in each group from challenge to first stool sample positive for *Salmonella* Typhimurium by culture and/or PCR<br>2 Duration of stool shedding.<br>3 The magnitude of stool shedding measured in CFU/ml in quantitative stool culture analysis
	Compare **microbiological** features of **bloodstream infection** following oral challenge with *S.* Typhimurium 4/74 or *S.* Typhimurium D23580 strains	*Salmonella* bloodstream infection after challenge, with specific reference to:<br>1 The proportion of participants in each group developing any bacteraemia<br>2 The severity of bacteraemia in each group as measured by time to onset, duration, clearance time, magnitude quantified in CFU/ml.
	Compare biochemical and haematological laboratory parameters following oral challenge.	Absolute values of laboratory parameters from time of challenge to Day 28 and/or Day 90, with specific reference to: Haemoglobin, white cell count and differential, urea and electrolytes, liver function tests.
	Describe and compare the serum antibody response following oral challenge with *S.* Typhimurium 4/74 or D23580 strains.	Serum samples measured at baseline and post challenge time points:<br>1 *S.* Typhimurium O-specific polysaccharide serum IgG and IgA concentration measured by ELISA<br>2 *S.* Typhimurium specific serum IgG and IgA concentration against other S. Typhimurium antigens measured by ELISA<br>3 Serum bactericidal antibody titres against *Salmonella* Typhimurium<br>4 *S.* Typhimurium specific antibody secreting cell and memory B-cell responses measured by ELISPOT<br>5 Other functional antibody activity measurements including systems serology platforms.
	Describe and compare the mucosal antibody response following oral challenge with *S.* Typhimurium 4/74 or D23580 strains.	Saliva and/or stool samples measured at baseline and post challenge time points:<br>1 S. Typhimurium specific IgG and IgA concentration measured by ELISA<br>2 Bactericidal antibody titres<br>3 Other functional antibody activity measurements including systems serology platforms
	Describe and compare cell-mediated immune response following oral challenge with *S.* Typhimurium 4/74 D23580 strains.	Absolute values and inter-group comparison of laboratory assays performed on peripheral blood mononuclear cells measured at baseline and post challenge time points:<br>1 Lymphocyte populations as measured by flow cytometry and/or CyTOF<br>2 *S.* Typhimurium specific cell-mediated immune responses as measured by ELISPOT and flow cytometry and/or CyTOF
	To describe participant experience following oral challenge with *S.* Typhimurium 4/74 or D23580 strains	Descriptive statistics following administration of a structured questionnaire at baseline and post-challenge.

*Severe gastroenteritis: ≥6 loose/liquid stools (Bristol Type 6-7) **and/or** >800 g of loose/liquid stools in a rolling 24-hour period **and/or** ≥2 stools with gross blood in 24 hours

**Moderate gastroenteritis: 4–5 loose/liquid stools (Bristol Type 6-7) and/or 400–800 g in a rolling 24-hour period

For peer review only - http://bmjopen.bmj.com/site/about/guidelines.xhtml

Table 2: Criteria for commencing antibiotic therapy following oral bacterial challenge

**Antibiotics are commenced if any of the following apply**
Any participant with *Salmonella* Typhimurium bacteraemia
Fever ≥38°C for ≥12hrs
Any participant with severe gastroenteritis*
Moderate gastroenteritis** plus:<br>• fever ≥38°C on one occasion and/or<br>• ≥1 Grade 2*** systemic symptoms
Any participant with 3 or more of the following symptoms on the same day at Grade 2 or higher.<br>• Headache<br>• Fatigue/Malaise<br>• Anorexia<br>• Abdominal pain<br>• Nausea<br>• Vomiting<br>• Myalgia<br>• Arthralgia<br>• Cough
Any participant from whom *Salmonella* has been detected from at least two stool culture/PCR and 24hrs apart who has not received antibiotics by day 14 post-challenge
Any participant in whom antibiotic use is felt to be clinically necessary (as decided by a medically qualified study doctor)
**Notes:**<br>*Severe gastroenteritis: ≥6 loose/liquid stools (Bristol Type 6-7) **and/or** >800 g of loose/liquid stools in a rolling 24-hour period **and/or** ≥2 stools with gross blood in 24 hours<br>**Moderate gastroenteritis: 4–5 loose/liquid stools (Bristol Type 6-7) and/or 400–800 g in a rolling 24-hour period<br>***Grade 0 = not present; Grade 1 = Present but no interference with activity; Grade 2 = Some interference with activity; Grade 3 = Significant; prevents daily activity; Grade 4: ED visit or hospitalisation

For peer review only

For peer review only - http://bmjopen.bmj.com/site/about/guidelines.xhtml

Table 3: Quarantine discharge criteria

**Participants will be discharged from the inpatient quarantine unit if the following criteria are met:**
• Medically fit for discharge in the opinion of the study physician **AND**
• Complete resolution of diarrhoea (Bristol stool type 6-7) for 48 hours **AND**
• Seven days (168 hours) have elapsed since challenge
**For patients diagnosed with Salmonellosis from day 0 to day 7, the following criteria apply:**
• Antibiotic treatment has been initiated and patient has completed 96hrs follow up
• Resolution of *Salmonella* Typhimurium bacteraemia (if applicable)

For peer review only - http://bmjopen.bmj.com/site/about/guidelines.xhtml

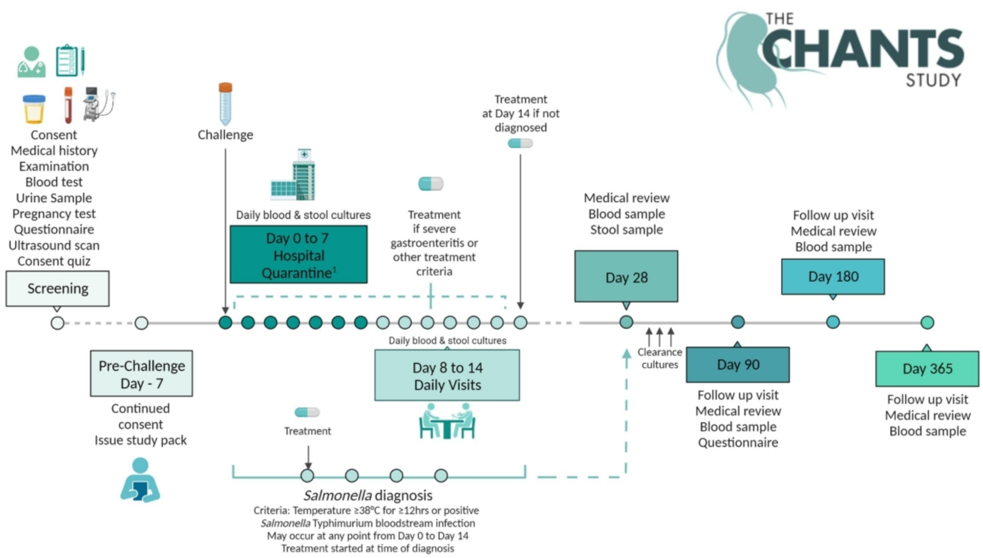

Overview of the participant journey from screening to study completion at 1 year of follow-up

269x159mm (220 x 220 DPI)

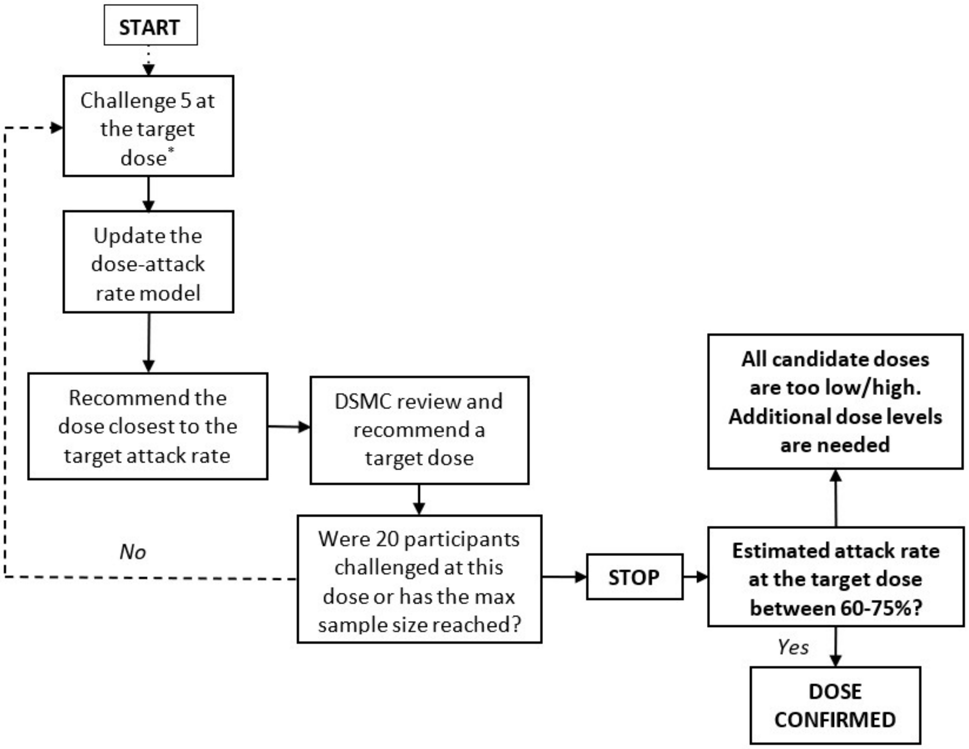

Decision making algorithm for dose escalation/de-escalation. Starting at 1-5 x10^3 CFU to reach the primary endpoint. *The target dose for the first cohort will be 1-5 x 10^3 CFU, and the first cohort of 5 participants will be divided into 3 groups (1, 2, and 2 participants) and challenged with an interval of at least 7 days. For the rest of the cohorts, they will be challenged on the same day, if possible.

149x117mm (144 x 144 DPI)

Supplementary Materials

Table 1: Exploratory Objectives

**Exploratory**	1	To investigate how the human microbiota interacts with oral challenge of *S.* Typhimurium 4/74 or D23580 strains	Metagenomic analysis of stool and/or saliva to measure constituent microbiological flora (and their metabolome/transcriptome) at baseline and post-challenge time points
	2	To investigate *Salmonella* Typhimurium virulence factors and gene expression	Exploratory analysis of stool samples at several time points following challenge to assess bacterial transcriptome by RNA-seq
	3	To describe and compare additional host immune responses following oral challenge of *S.* Typhimurium 4/74 or D23580 strains.	Analysis of B-cell repertoire, plasma cytokine profile and kinetics.
	4	To evaluate environmental contamination with *Salmonella enterica* in the near patient environment	The pattern and proportion of environmental samples testing positive for *Salmonella* Typhimurium by culture or molecular methods.
	5	To evaluate host-pathogen interactions following *Salmonella* infection including the presence and mechanisms of *Salmonella* persister infected macrophages.	Exploratory analysis of infected immune cells from blood including, but not limited to, single-cell RNA-seq.

For peer review only

Table 2: Eligibility criteria for participation

**Inclusion Criteria**
Agree to give informed consent for participation in the study.
Aged between 18 and 50 years inclusive at time of challenge.
In good health as determined by medical history, physical examination, and clinical judgment of the study team.
Agree (in the study team's opinion) to comply with all study requirements, including capacity to adhere to good personal hygiene and infection control precautions.
Agree to allow his or her General Practitioner (and/or Consultant if appropriate), to be notified of participation in the study.
Agree to allow study staff to contact his or her GP to access the participant's medical history and vaccination records.
Agree to allow UKHSA and the local health protection unit to be informed of their participation in the study.
Agree to give his or her close contacts written information informing them of the participant's involvement in the study and offer them voluntary screening for Salmonella carriage.
Agree to a period of inpatient quarantine whilst symptomatic (In exceptional circumstances, the duration of inpatient quarantine may be extended.)
Agree to have 24-hour contact with study staff during the four weeks post challenge and can ensure that they are contactable by mobile phone for the duration of the study period until antibiotic completion.
Agree to allow the study team to hold the name and 24-hour contact number of a close friend, relative or housemate who will be kept informed of the study participant's whereabouts for the duration of the challenge period after quarantine (from the time of challenge until completion of antibiotic course). This person will be contacted if study staff are unable to contact the participant after discharge from inpatient quarantine.
Have internet access to allow completion of the e-diary and real-time safety monitoring.
Agree to avoid antipyretic/anti-inflammatory treatment from the time of challenge (Day 0) until advised by a study doctor or until 14 days after challenge.
Agree to refrain from donating blood for the duration of the study.
Agree to provide their National Insurance/Passport number for the purposes of Trial over-volunteering prevention system (TOPS) registration and for payment of reimbursement expenses.
Participants must have received at least two doses of a SARS-CoV-2 vaccines that has been approved for use by the MHRA (or other national regulatory authority) four weeks prior to enrolment.
Proficient in English at a level sufficient to understand, retain, weigh up and communicate the study details as outlined in the participant information sheet, in the opinion of the study investigator.
**Exclusion Criteria**
History of microbiologically confirmed *Salmonella* infection[1].
History of receipt of oral Ty21a typhoid vaccination
History of **significant[2]** organ-specific and/or systemic disease that could interfere with trial conduct or completion. Including, for example, but not restricted to:<br>- Cardiovascular disease (ischaemic heart disease, valvular heart disease, vascular disease including aneurysmal disease or endovascular prostheses, prolonged corrected QT interval (>450 milliseconds) on ECG screening).

For peer review only

- Respiratory disease[3]<br>- Haematological disease including sickle cell disease and sickle cell trait.<br>- Endocrine disorders, including specifically diabetes mellitus.<br>- Renal or bladder disease, including history of renal calculi.<br>- Biliary tract disease, including specifically colic, asymptomatic gallstones, cholecystectomy.<br>- Gastro-intestinal disease including specifically: a current requirement for antacids, H_2-receptor antagonists, proton pump inhibitors or laxatives, Inflammatory bowel disease, confirmed diagnosis of irritable bowel syndrome as defined by the Rome IV criteria,<br>- Neurological disease<br>- Metabolic disease<br>- Autoimmune disease<br>- Psychiatric illness requiring hospitalisation.<br>- Known or suspected drug and/or alcohol misuse disorder<br>- Chronic/Active Infectious disease including active tuberculosis.<br>- Severe infection requiring hospitalisation for intravenous antibiotics within the last 10 years. Exceptions to this would include a short course of intravenous antibiotics for appendicitis, biliary sepsis, diverticulitis, and cellulitis.<br>- History of recent malaria infection in the past 12 months from screening<br>- History of joint replacement<br>- History of any orthopaedic/osseous implanted prosthesis<br>- Presence of any other internal implanted device e.g., permanent pacemaker.
Have any known or suspected impairment of immune function (as defined in the green book[140]), alteration of immune function, or prior immune exposure that may alter immune function to *Salmonella* infection resulting from, for example:<br>- Congenital or acquired immunodeficiency, including IgA deficiency.<br>- Human Immunodeficiency Virus infection or symptoms/signs suggestive of an HIV-associated condition<br>- Evidence of severe primary immunodeficiency, for example, severe combined immunodeficiency, Wiskott-Aldrich syndrome, and other combined immunodeficiency syndromes.<br>- Currently being treated for malignant disease with immunosuppressive chemotherapy or radiotherapy, or who have received such treatment within at least the last six months.<br>- Individuals who have received a solid organ transplant and are currently on immunosuppressive treatment.<br>- Individuals who have received a bone marrow transplant, until within 12 months of finishing all immunosuppressive treatment,<br>- Individuals receiving systemic high dose steroids[4] until at least three months after treatment has stopped.<br>- Individuals receiving other types of immunosuppressive[5] drugs (alone or in combination with lower doses of steroids) until at least six months after terminating such treatment.<br>- Receipt of immunoglobulin or any blood product transfusion within 3 months of study start.<br>- History of cancer (except squamous cell or basal cell carcinoma of the skin and cervical carcinoma in situ).
Moderate or severe depression or anxiety as classified by the Hospital Anxiety and Depression Score at screening or challenge that is deemed clinically significant by the study doctors[6].
Weight less than 50kg[7]
Anyone taking long-term medication (e.g., analgesia, anti-inflammatories, or antibiotics) that may affect symptom reporting or interpretation of the study results.
Contraindication to cephalosporin, fluroquinolone, or macrolide antibiotics.

For peer review only

Female participants who are pregnant, lactating or who are unwilling to ensure that they or their partner use effective contraception[8] 30 days prior to challenge and continue to do so until two negative stool samples, a minimum of 3 weeks after completion of antibiotic treatment, have been obtained.
Full-time, part-time, or voluntary occupations involving:<br>- Clinical healthcare work[9]<br>- Social work with direct contact with young children (defined as those attending pre-school groups or nursery or aged under 2 years), or other clinically vulnerable children, adolescents.<br>- Clinical or social work with direct contact with highly susceptible patients or persons in whom *Salmonella* infection would have particularly serious consequences (unless willing to avoid work until demonstrated not to be infected with *Salmonella* in accordance with guidance from Public Health England and willing to allow study staff to inform their employer).
Full time, part time or voluntary occupations involving:<br>- Commercial food handling (involving preparing or serving unwrapped foods not subjected to further heating)
Close household contact with:<br>- Children aged less than 2 years<br>- Any individual who is immunocompromised.<br>- Pregnant women<br>- Household contacts aged over 70 years
Scheduled elective surgery or other procedures requiring general anaesthesia during the study period.
Participants who have participated in another research study involving an investigational product that might affect risk of *Salmonella* infection or compromise the integrity of the study within the 30 days prior to enrolment (e.g. significant volumes of blood already taken in previous study)[10].
Detection of any abnormal results from screening investigations, unless deemed not clinically significant.
Screening blood test positive for HLA-B*27 Antigen
Any other social, psychological or health issues which, in the opinion of the study staff, may:<br>- Put the participant or their contacts at risk because of participation in the study.<br>- Adversely affect the interpretation of the primary endpoint data.<br>- Impair the participant's ability to participate in the study.
Having previously received any experimental *Salmonella* vaccine as part of a clinical trial
Have participated in previous *Salmonella* Typhi or Paratyphi challenge studies (with ingestion of challenge agent).
Any employee of the sponsor or research site personnel directly affiliated with this study or their immediate family members.
Inability to comply with any of the study requirements (at the discretion of the study staff).

[1] Including enteric fever (*Salmonella* Typhi and/or *Salmonella* Paratyphi infection)

[2] Individuals with specific medical co-morbidities (e.g., childhood asthma) will be considered for enrolment after review with the principal investigator and clinical oversight group, following discussion with their GP and/or consultant as appropriate. The rationale for enrolment in these cases will be recorded in the CRF.

[3] Participants with well controlled asthma may be considered for enrolment at the discretion of the study investigators and following consultation with their general practitioner.

[4] Defined as those receiving at least 40mg of prednisolone per day for more than one week or equivalent.

[5] Azathioprine, cyclosporin, methotrexate, cyclophosphamide, leflunomide and other cytokine inhibitors.

For peer review only

[6] If elevated scores are due to temporary significant life events, the questionnaire may be repeated after resolution of the event with a view to inclusion if normal.
[7] Or a Body Mass Index (BMI) that, in the opinion of the study doctors, may adversely impair the interpretation of the study results or affect the safe performance of any study procedures.
[8] All forms of contraception are considered effective with the exception of barrier methods and natural family planning. Accepted methods include hormonal contraceptive pills, intrauterine contraceptive devices/systems, long-acting injectable hormonal contraception, contraceptive implants.
[9] This includes medical, dental, nursing or midwifery students (or other allied-healthcare professionals) with direct patient contact. Such students who are not undertaking patient-facing rotations until demonstrated not to be infected with *Salmonella* species may be considered for enrolment.
[10] As assessed by both participant questioning and registration with The Over Volunteering Prevention System (TOPS) database.

Table 3: Temporary Exclusion Criteria at time of bacterial challenge

**Temporary Exclusion Criteria at Challenge**
Positive lateral flow test for SARS-CoV-2
Significant acute or acute-on-chronic infection within the previous 7 days or have experienced fever (>37.5°C) or subjective febrile symptoms within the previous 3 days
History of any antibiotic therapy during the previous 5 days
Any systemic corticosteroid (or equivalent) treatment in the previous 14 days, or for more than seven consecutive days within the past 3 months
Therapy with antacids, proton pump inhibitors or H2-receptor antagonists within 24 hours prior to challenge
Detection of gastrointestinal pathogens (other than *Salmonella*) in stool culture/PCR collected at the pre-challenge assessment (Day-7) including *Shigella* spp., *Campylobacter* spp., *E. Coli* O157, *Giardia* spp and *Cryptosporidium* spp, until two subsequent samples are negative. Detection of *Salmonella* spp. in stool prior to challenge (Day – 7) will result in exclusion from the study
Detection of extended spectrum beta lactamase producing organisms (ESBLs) or Carbapenem resistant organisms (CRO) until two subsequent samples are negative.

For peer review only

Table 4: Clinical sample collection plan for patients undergoing primary challenge. Values correspond to volume of blood collected in millilitres.

	Pre-challenge		Challenge period																				Follow up			
			Inpatient quarantine								Outpatient assessments							Post diagnosis/treatment								
	Screening	Day -7	Day 0	Day 1	Day 2	Day 3	Day 4	Day 5	Day 6	Day 7	Day 8	Day 9	Day 10	Day 11	Day 12	Day 13	Day 14	SD	SD +24h	SD +48h	SD +72h	SD +96h	Day 28	Day 90	Day 180	Day 365
Stool sample		x	x	x	x	x	x	x	x	x	x	x	x	x	x	x	x	x	x	x	x	x	x	x	x	x
Blood culture BACTEC			10	10	10	10	10	10	10	10	10	10	10	10	10	10	10	10	10	10	10	10				
FBC	1	1	1		1		1		1		1		1		1		1	1	1	1	1	1	1	1	1	
U&E/CRP/LFT	2	2	2		2		2		2		2		2		2		2	2	2	2	2	2	2	2	2	
Additional screening investigations*	10																									
Bacterial quantification																		10								
Serum sample			10							10							10	10					10	10	10	10
Plasma Sample			3							3							3	3					3			
PBMC			25				25			25							25	25					25	25	25	25
Functional genomics			3	3	3	3				3								3					3	3		
Whole blood PCR			10	10	10	10	10	10	10	10																
DNA sample (including epigenetics)			x																				x		x	
Saliva sample			x							x							x						x		x	

*HIV/HBV/HV serology, HLA-B27, Haemoglobinopathy screen, Coeliac serology, HbA1c

Table 5: Simulation results comparing CRM with rule-based model.

Operating characteristics for CRM-based approach under 5 following scenarios:

Scenario 1 target dose at dose 1 with attack rate: 0.675, 0.8, 0.9, 0.95.
Scenario 2 target dose at dose 2 with attack rate: 0.5, 0.675, 0.8, 0.9.
Scenario 3 target dose at dose 4 with attack rate: 0.3, 0.4, 0.5, 0.675.
Scenario 4 target dose beyond dose 4 with attack rate: 0.2, 0.3, 0.4, 0.5.
Scenario 5 target dose under dose 1 with attack rate: 0.8, 0.9, 0.95, 0.975.

	**CRM-based***	**Rule-based**
**Scenario 1**		
Median sample size (IQR)	20 (20-25)	20 (20-25)
Probability of choosing the dose		
First dose too high	17.2%	16.4%
1 (67.5%)	69.1%	58.4%
2 (80%)	13.7%	8.6%
3 (90%)		
4 (95%)		
Fail to find a dose		16.6%
**Scenario 2**		
Median sample size (IQR)	30 (25-35)	30 (25-35)
Probability of choosing the dose		
First dose too high	0.7%	0.8%
1 (50%)	20.6%	17.3%
2 (67.5%)	64.9%	49.4%
3 (80%)	13.7%	3.9%
4 (90%)	0.1%	
Failed to find a dose	N/A	28.6%
**Scenario 3**		
Median sample size	35 (35-40)	30 (20-40)
Probability of choosing the dose		
1 (30%)	0.02%	0.6%
2 (40%)	2.2%	4.4%
3 (50%)	34.1%	14.1%
4 (67.5%)	59.1%	11.0%
Last dose too low	4.4%	0%
Fail to find a dose	N/A	69.9%
**Scenario 4**		
Median sample size	35 (35-40)	30 (25-35)
Probability of choosing the dose		
1 (20%)	0%	0%
2 (30%)	0.1%	0.8%
3 (40%)	5.3%	3.1%
4 (50%)	46.0%	10.7%
Last dose too low	48.6%	

For peer review only

Fail to find a dose	N/A	85.4%
**Scenario 5**		
Median sample size	20 (20-20)	20 (20-20)
Probability of choosing the dose		
First dose too high	64.7%	63.2%
1 (80%)	35.0%	32.3%
2 (90%)	0.3%	0.1%
3 (95%)	0%	0%
4 (97.5%)	0%	0%
Fail to find a dose	N/A	4.4%

*Parameters for the CRM-based design:

- Prior attack rate at the four doses: 0.6, 0.75, 0.85, 0.9;
- The attack rate is 67.5%;
- Starting at dose 1

Table 6 – ICMJE Data Availability Statement

**Which data in particular will be shared?**	**Are individual deidentified participant data (including data dictionaries) available?**	**Are additional, related documents available (e.g., study protocol, statistical analysis plan, etc.)?**	**When will the data become available and for how long?**	**By what access criteria will the data be shared (including with whom, for what types of analyses, and by what mechanism)?**
Individual participant data that underlie the results reported in the trial, after deidentification	Yes, pertaining to established endpoints of the trial	Study protocol	Following publication of the trial, ending 36 months from publication date	Investigators whose proposed use of data has been approved by an independent ethics review committee, identified for this purpose

For peer review only

Figure 1: Antibiotic Treatment Algorithm

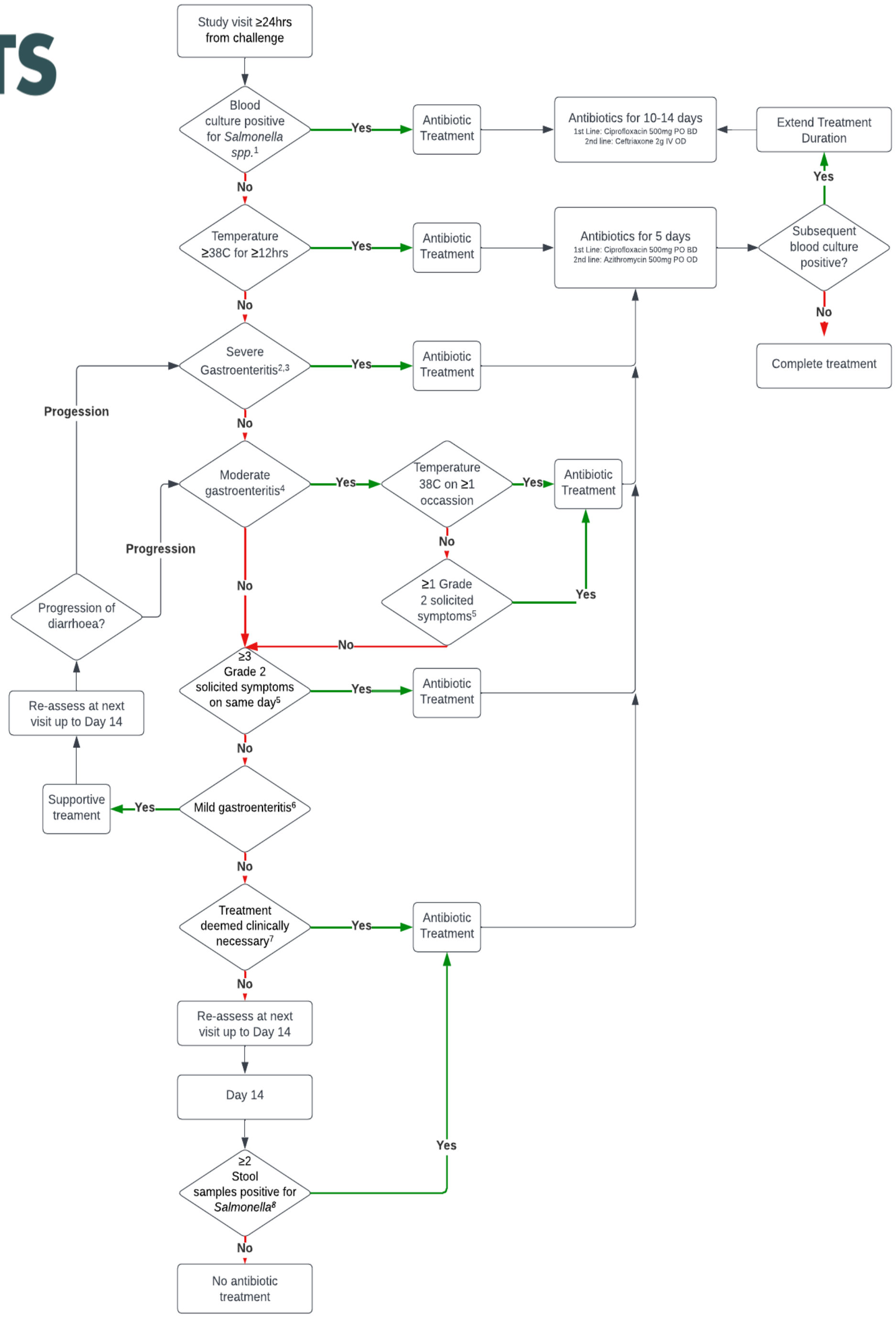

Notes

1 - Treatment initiated upon identification of Gram negative rods in blood culture, prior to formal speciation.

2- Loose/liquid stools defined as Type 6 or 7 stools on the Bristol stool chart.

3 - Severe gastroenteritis defined over a rolling 24-hour period as:
- ≥6 loose/liquid stools **and/or**
- >800 g of loose/liquid stools **and/or**
- ≥2 stools with gross blood

4 - Moderate Gastroenteritis defined over a rolling 24-hour period as:
- 4-5 loose/liquid stools **and/or**
- 400-800g loose/liquid stools

5 - Solicited symptoms defined as:
- Headache
- Fatigue/Malaise
- Anorexia
- Abdominal pain
- Nausea
- Vomiting
- Myalgia
- Arthralgia
- Cough

6 - Mild Gastroenteritis defined over a rolling 24-hour period as:
- 3 loose/liquid stools **and/or**
- 200-399g loose/liquid stools

7 - At the discretion of the principal investigator in consultation with the trial steering committee

8 - As detected by culture and/or PCR

Figure 2: Quarantine Discharge Algorithm

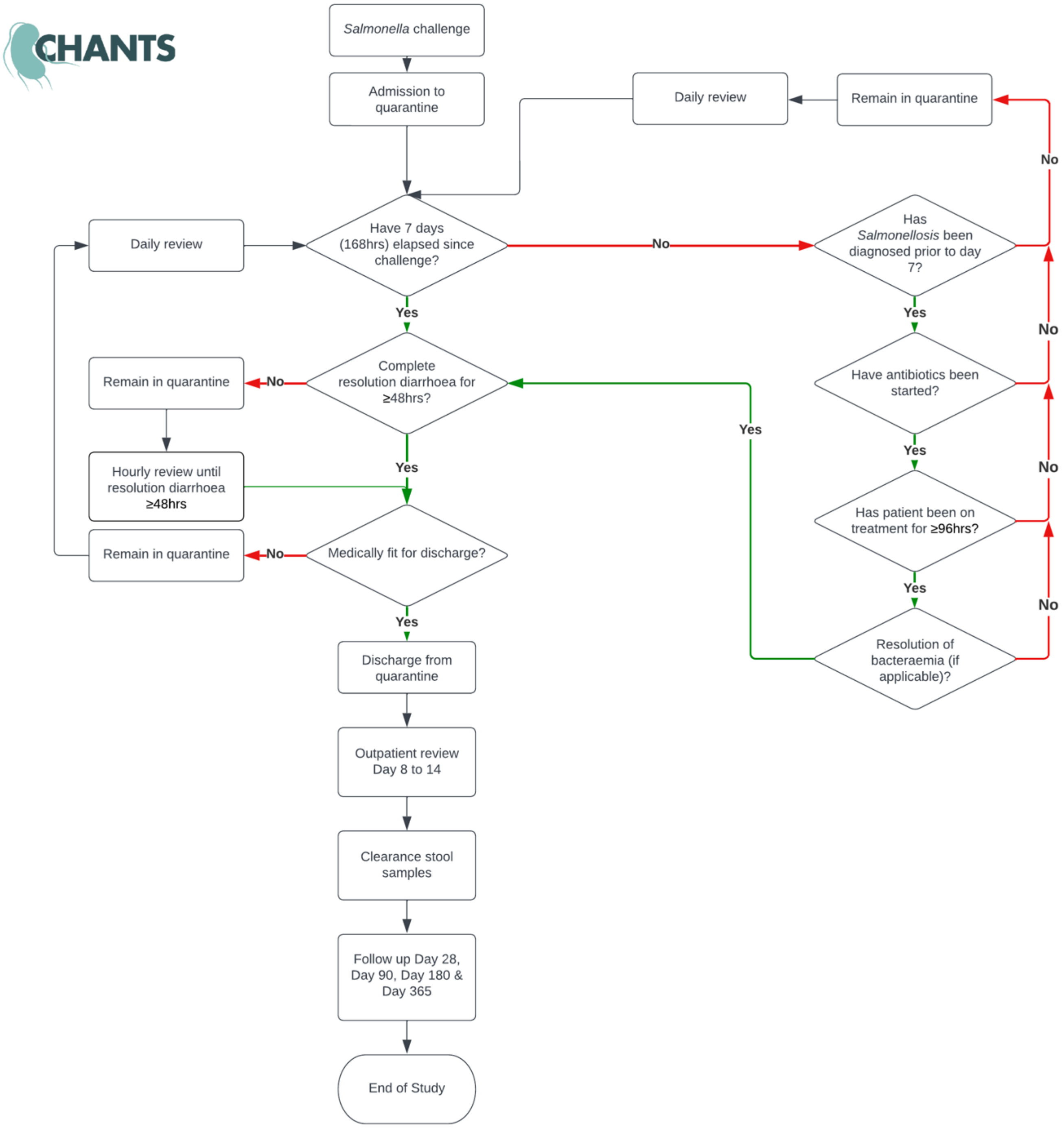

SPIRIT

STANDARD PROTOCOL ITEMS: RECOMMENDATIONS FOR INTERVENTIONAL TRIALS

SPIRIT 2013 Checklist: Recommended items to address in a clinical trial protocol and related documents*

Section/item	Item No.	Description	Addressed on Page
**Administrative information**			
Title	1	Descriptive title identifying the study design, population, interventions, and, if applicable, trial acronym	1
Trial registration	2a	Trial identifier and registry name. If not yet registered, name of intended registry	2
	2b	All items from the World Health Organization Trial Registration Data Set	2
Protocol version	3	Date and version identifier	1
Funding	4	Sources and types of financial, material, and other support	17
Roles and responsibilities	5a	Names, affiliations, and roles of protocol contributors	1
	5b	Name and contact information for the trial sponsor	1
	5c	Role of study sponsor and funders, if any, in study design; collection, management, analysis, and interpretation of data; writing of the report; and the decision to submit the report for publication, including whether they will have ultimate authority over any of these activities	17

For peer review only

	5d	Composition, roles, and responsibilities of the coordinating centre, steering committee, endpoint adjudication committee, data management team, and other individuals or groups overseeing the trial, if applicable (see Item 21a for data monitoring committee)	8, 11
**Introduction**			
Background and rationale	6a	Description of research question and justification for undertaking the trial, including summary of relevant studies (published and unpublished) examining benefits and harms for each intervention	4-5
	6b	Explanation for choice of comparators	5, 7
Objectives	7	Specific objectives or hypotheses	6, 13
Trial design	8	Description of trial design including type of trial (eg, parallel group, crossover, factorial, single group), allocation ratio, and framework (eg, superiority, equivalence, noninferiority, exploratory)	5-9
**Methods: Participants, interventions, and outcomes**			
Study setting	9	Description of study settings (eg, community clinic, academic hospital) and list of countries where data will be collected. Reference to where list of study sites can be obtained	5

For peer review only

Eligibility criteria	10	Inclusion and exclusion criteria for participants. If applicable, eligibility criteria for study centres and individuals who will perform the interventions (eg, surgeons, psychotherapists)	6-7 (and supplementary table 1)
Interventions	11a	Interventions for each group with sufficient detail to allow replication, including how and when they will be administered	8-9
	11b	Criteria for discontinuing or modifying allocated interventions for a given trial participant (eg, drug dose change in response to harms, participant request, or improving/worsening disease)	N/A
	11c	Strategies to improve adherence to intervention protocols, and any procedures for monitoring adherence (eg, drug tablet return, laboratory tests)	N/A
	11d	Relevant concomitant care and interventions that are permitted or prohibited during the trial	9-10
Outcomes	12	Primary, secondary, and other outcomes, including the specific measurement variable (eg, systolic blood pressure), analysis metric (eg, change from baseline, final value, time to event), method of aggregation (eg, median, proportion), and time point for each outcome. Explanation of the clinical relevance of chosen efficacy and harm outcomes is strongly recommended	6 (and table 1)
Participant timeline	13	Time schedule of enrolment, interventions (including any run-ins and washouts), assessments, and visits for participants. A schematic diagram is highly recommended (see Figure)	Figure 1
Sample size	14	Estimated number of participants needed to achieve study objectives and how it was determined, including clinical and statistical assumptions supporting any sample size calculations	11-12
Recruitment	15	Strategies for achieving adequate participant enrolment to reach target sample size	6-7

Methods: Assignment of interventions (for controlled trials)

Allocation:			
Sequence generation	16a	Method of generating the allocation sequence (eg, computer-generated random numbers), and list of any factors for stratification. To reduce predictability of a random sequence, details of any planned restriction (eg, blocking) should be provided in a separate document that is unavailable to those who enrol participants or assign interventions	8
Allocation concealment mechanism	16b	Mechanism of implementing the allocation sequence (eg, central telephone; sequentially numbered, opaque, sealed envelopes), describing any steps to conceal the sequence until interventions are assigned	8
Implementation	16c	Who will generate the allocation sequence, who will enrol participants, and who will assign participants to interventions	8
Blinding (masking)	17a	Who will be blinded after assignment to interventions (eg, trial participants, care providers, outcome assessors, data analysts), and how	8
	17b	If blinded, circumstances under which unblinding is permissible, and procedure for revealing a participant's allocated intervention during the trial	8

Methods: Data collection, management, and analysis

Data collection methods	18a	Plans for assessment and collection of outcome, baseline, and other trial data, including any related processes to promote data quality (eg, duplicate measurements, training of assessors) and a description of study instruments (eg, questionnaires, laboratory tests) along with their reliability and validity, if known. Reference to where data collection forms can be found, if not in the protocol	Table 1, Figure 1

For peer review only - http://bmjopen.bmj.com/site/about/guidelines.xhtml

	18b	Plans to promote participant retention and complete follow-up, including list of any outcome data to be collected for participants who discontinue or deviate from intervention protocols	Figure 1
Data management	19	Plans for data entry, coding, security, and storage, including any related processes to promote data quality (eg, double data entry; range checks for data values). Reference to where details of data management procedures can be found, if not in the protocol	11
Statistical methods	20a	Statistical methods for analysing primary and secondary outcomes. Reference to where other details of the statistical analysis plan can be found, if not in the protocol	11-12, Figure 2
	20b	Methods for any additional analyses (eg, subgroup and adjusted analyses)	Table 1
	20c	Definition of analysis population relating to protocol non-adherence (eg, as randomised analysis), and any statistical methods to handle missing data (eg, multiple imputation)	N/A
**Methods: Monitoring**			
Data monitoring	21a	Composition of data monitoring committee (DMC); summary of its role and reporting structure; statement of whether it is independent from the sponsor and competing interests; and reference to where further details about its charter can be found, if not in the protocol. Alternatively, an explanation of why a DMC is not needed	11
	21b	Description of any interim analyses and stopping guidelines, including who will have access to these interim results and make the final decision to terminate the trial	11

Harms	22	Plans for collecting, assessing, reporting, and managing solicited and spontaneously reported adverse events and other unintended effects of trial interventions or trial conduct	11
Auditing	23	Frequency and procedures for auditing trial conduct, if any, and whether the process will be independent from investigators and the sponsor	8, 11
**Ethics and dissemination**			
Research ethics approval	24	Plans for seeking research ethics committee/institutional review board (REC/IRB) approval	12
Protocol amendments	25	Plans for communicating important protocol modifications (eg, changes to eligibility criteria, outcomes, analyses) to relevant parties (eg, investigators, REC/IRBs, trial participants, trial registries, journals, regulators)	12
Consent or assent	26a	Who will obtain informed consent or assent from potential trial participants or authorised surrogates, and how (see Item 32)	6-7
	26b	Additional consent provisions for collection and use of participant data and biological specimens in ancillary studies, if applicable	N/A
Confidentiality	27	How personal information about potential and enrolled participants will be collected, shared, and maintained in order to protect confidentiality before, during, and after the trial	11
Declaration of interests	28	Financial and other competing interests for principal investigators for the overall trial and each study site	17
Access to data	29	Statement of who will have access to the final trial dataset, and disclosure of contractual agreements that limit such access for investigators	N/A

For peer review only

For peer review only - http://bmjopen.bmj.com/site/about/guidelines.xhtml

Ancillary and post-trial care	30	Provisions, if any, for ancillary and post-trial care, and for compensation to those who suffer harm from trial participation	12
Dissemination policy	31a	Plans for investigators and sponsor to communicate trial results to participants, healthcare professionals, the public, and other relevant groups (eg, via publication, reporting in results databases, or other data sharing arrangements), including any publication restrictions	12
	31b	Authorship eligibility guidelines and any intended use of professional writers	17
	31c	Plans, if any, for granting public access to the full protocol, participant-level dataset, and statistical code	N/A
**Appendices**			
Informed consent materials	32	Model consent form and other related documentation given to participants and authorised surrogates	Supplementary
Biological specimens	33	Plans for collection, laboratory evaluation, and storage of biological specimens for genetic or molecular analysis in the current trial and for future use in ancillary studies, if applicable	Figure 1, Table 1

*It is strongly recommended that this checklist be read in conjunction with the SPIRIT 2013 Explanation & Elaboration for important clarification on the items. Amendments to the protocol should be tracked and dated. The SPIRIT checklist is copyrighted by the SPIRIT Group under the Creative Commons “Attribution-NonCommercial-NoDerivs 3.0 Unported” license.

For peer review only

BMJ Open

Protocol for the Challenge Nontyphoidal *Salmonella* (CHANTS) Study: a first-in-human, in-patient, double-blind, randomised, safety and dose-escalation controlled human infection model in the United Kingdom

Journal:	*BMJ Open*
Manuscript ID	bmjopen-2023-076477.R2
Article Type:	Protocol
Date Submitted by the Author:	08-Dec-2023

Complete List of Authors:	Smith, Christopher; Imperial College London, Department of Infectious Disease<br>Smith, Emma; Imperial College London, Department of Infectious Disease<br>Rydlova, Anna; Imperial College London, Department of Infectious Disease<br>Varro, Robert; Imperial College London, Department of Infectious Disease<br>Hinton, Jay; University of Liverpool, Institute of Infection, Veterinary and Ecological Sciences<br>Gordon, Melita; University of Liverpool, Institute of Infection, Veterinary and Ecological Sciences; Kamuzu University of Health Sciences, Malawi Liverpool Wellcome Trust Clinical Research Programme<br>Choy, Robert; PATH<br>Liu , Xinxue ; Oxford University, Oxford Vaccine Group, Department of Paediatrics ; NIHR Oxford Biomedical Research Centre<br>Pollard, Andrew; Oxford University, Oxford Vaccine Group, Department of Paediatrics; NIHR Oxford Biomedical Research Centre<br>Chiu, Christopher; Imperial College London, Department of Infectious Disease<br>Cooke, Graham; Imperial College London, Department of Infectious Disease<br>Gibani, Malick; Imperial College London, Department of Infectious Disease

**Primary Subject Heading**:	Infectious diseases
Secondary Subject Heading:	Immunology (including allergy), Global health
Keywords:	INFECTIOUS DISEASES, Tropical medicine < INFECTIOUS DISEASES, Neglected Diseases, Microbiology < NATURAL SCIENCE DISCIPLINES, Immunology < NATURAL SCIENCE DISCIPLINES

SCHOLARONE™
Manuscripts

Protocol for the Challenge Nontyphoidal *Salmonella* (CHANTS) Study: a first-in-human, in-patient, double-blind, randomised, safety and dose-escalation controlled human infection model in the United Kingdom.

Authors

Christopher Smith[1], Emma Smith[1], Anna Rydlova[1], Robert Varro[1], Jay C.D. Hinton[2], Melita A. Gordon[2,3], Robert K.M. Choy[4], Xinxue Liu[5,6], Andrew J. Pollard[5,6], Christopher Chiu[1], Graham S. Cooke[1], Malick M. Gibani[1]

[1]Department of Infectious Disease, Imperial College London, UK
[2]Institute of Infection, Veterinary and Ecological Sciences, University of Liverpool, UK
[3]Malawi-Liverpool-Wellcome Trust Clinical Research Programme, Malawi
[4]PATH, Seattle, USA
[5]Oxford Vaccine Group, Department of Paediatrics, University of Oxford, UK
[6]NIHR Oxford Biomedical Research Centre, Oxford, UK

Corresponding Author

Malick M. Gibani, Department of Infectious Disease, Imperial College London, St Mary's Campus, Norfolk Place, London, W2 1PG: m.gibani@imperial.ac.uk.

Keywords

Salmonella; *Salmonella* Typhimurium; Non-Typhoidal *Salmonella*; Human Challenge Model; Controlled Human Infection Model; Vaccines.

Protocol Version: 2.0

Study Sponsor

Imperial College London is the main research Sponsor for this study. For further information regarding the sponsorship conditions, please contact the Head of Regulatory Compliance at:

Research Governance and Integrity Team
Imperial College London and Imperial College Healthcare NHS Trust
Room 215, Level 2, Medical School Building
Norfolk Place
London, W2 1PG
Tel: 0207 594 1862

For peer review only - http://bmjopen.bmj.com/site/about/guidelines.xhtml

1 ABSTRACT

Introduction

Invasive nontyphoidal *Salmonella* (iNTS) serovars are a major cause of community-acquired bloodstream infections in sub-Saharan Africa (SSA). In this setting, *Salmonella enterica* serovar Typhimurium accounts for two-thirds of infections and is associated with an estimated case-fatality rate of 15-20%. Several iNTS vaccine candidates are in early-stage assessment which – if found effective – would provide a valuable public health tool to reduce iNTS disease burden. The CHANTS study aims to develop a first-in-human *Salmonella* Typhimurium controlled human infection model (CHIM) which can act as a platform for future vaccine evaluation, in addition to providing novel insights into iNTS disease pathogenesis.

Methods and Analysis

This double-blind, safety and dose-escalation study will randomise 40-80 healthy UK participants aged 18-50 to receive oral challenge with one of two strains of *S.* Typhimurium belonging to the ST19 (strain 4/74) or ST313 (strain D23580) lineages. 4/74 is a global strain often associated with diarrhoeal illness predominantly in high-income settings, whilst D23580 is an archetypal strain representing invasive disease-causing isolates found in SSA. The primary objective is to determine the minimum infectious dose (CFU) required for 60-75% of participants to develop clinical or microbiological features of systemic salmonellosis. Secondary endpoints are to describe and compare the clinical, microbiological, and immunological responses following challenge. Dose escalation or de-escalation will be undertaken by continual-reassessment methodology and limited within pre-specified safety thresholds. Exploratory objectives are to describe mechanisms of iNTS virulence, identify putative immune correlates of protection, and describe host-pathogen interactions in response to infection.

Ethics and Dissemination

Ethical approval has been obtained from the NHS Health Research Authority (London – Fulham Research Ethics Committee 21/PR/0051; IRAS Project ID 301659). The study findings will be disseminated in international peer-reviewed journals and presented at national/international stakeholder meetings. Study outcome summaries will be provided to both funders and participants.

Trial Registration

Clinicaltrials.gov (NCT05870150).

For peer review only - http://bmjopen.bmj.com/site/about/guidelines.xhtml

2 STRENGTHS AND LIMITATIONS

1. This first-in-human NTS CHIM utilises well characterised *S.* Typhimurium strains (ST19 and ST313) which are representative of currently circulating isolates accountable for both diarrhoeal and invasive disease phenotypes.
2. A double-blind, randomised study design permits objective evaluation of differential clinical phenotypes induced by bacterial challenge.
3. The continual reassessment method for dose-finding, compared with rule-based design, improves trial efficiency, and increases the probability of finding the correct challenge dose.
4. This study is limited by recruitment of healthy UK volunteers, whose responses to challenge will differ from children and immunocompromised adults most commonly affected by iNTS in sub-Saharan Africa.

For peer review only

For peer review only - http://bmjopen.bmj.com/site/about/guidelines.xhtml

3 INTRODUCTION

Nontyphoidal *Salmonella* (NTS) infections are most commonly associated with a self-limiting diarrhoeal illness (dNTS) [1]. In contrast, invasive nontyphoidal *Salmonella* disease (iNTS) typically presents as a non-specific febrile illness – often without diarrhoea – following invasion into sterile sites causing bloodstream infection (BSI), meningitis, or sepsis [2]. iNTS is particularly common in sub-Saharan Africa (SSA) where most recent estimates reported 535,000 cases, 77,500 deaths, and 4·26 million lost DALYs in 2017 [3]. Case fatality rates are estimated to range between 15-20%. The high case fatality reflects that invasive disease occurs almost exclusively in individuals with impaired immunity. Recognised host risk factors include malnutrition, malaria, and chronic anaemia in children, and HIV infection and malignancy in adults [2,4]. Effective treatment is also increasingly challenging; highlighted by the WHO's inclusion of *Salmonella enterica* as one of the priority pathogens posing the greatest risk to human health through expanding antimicrobial resistance (AMR) [5].

iNTS serovars isolated in community-acquired BSI exhibit geographic variation. In Africa, *Salmonella enterica* subspecies *enterica* serovars Typhimurium and Enteritidis (henceforth *S.* Typhimurium and *S.* Enteritidis) are responsible for >90% of all iNTS BSI, with *S.* Typhimurium accounting for two thirds of this burden [6–8]. Sequencing of isolates from across Africa has identified *S.* Typhimurium multilocus sequence type (ST) 313 as the major cause of invasive disease, which is distinct from sequence types 19 and 34 that are more frequently associated with diarrhoeal disease (dNTS) in high-income settings such as the UK. ST313 has been shown to have undergone sequential evolutionary changes that converge towards a genotype associated with invasive disease [9].

To date, there are no licensed iNTS vaccines available for use in humans. Development of effective vaccines is urgently required and may contribute to reduced case numbers, reduced antibiotic use, and prevention of antimicrobial resistance [10]. Several candidate vaccines are under evaluation, including in bivalent or multi-valent formulations which could target both typhoidal and nontyphoidal *Salmonella* serovars [11]. Vaccine development for iNTS is hampered – in part – by an incomplete understanding of mechanisms and determinants of immunity during natural infection. Investigation into these mechanisms could support iNTS vaccine development programmes through identification of immune correlates of protection. In addition, vaccine development is hindered by the substantial time and financial commitments required to conduct large-scale phase III trials in field settings to obtain efficacy data.

For peer review only - http://bmjopen.bmj.com/site/about/guidelines.xhtml

Controlled human infection models (CHIM) have a strong track record in accelerating vaccine development, particularly for enteric pathogens [12,13]. For example, the live attenuated cholera vaccine CVD 103-HgR was licensed as a travel vaccine on the basis of safety, immunogenicity and efficacy data arising from CHIM studies [14]. More recently, a programme of typhoidal *Salmonella* CHIM studies demonstrated efficacy of a Vi-tetanus toxoid (Vi-TT) conjugate vaccine against *S.* Typhi (Typbar-TCV, Bharat Biotech) [15]. These data contributed to recommendations from WHO SAGE to support programmatic use of Vi-TT in high burden settings and were supported by subsequent phase III trials demonstrating similar efficacy in Nepal, Bangladesh, and Malawi [16–18].

The CHANTS study has been funded by the Wellcome Trust to establish a first-in-human NTS CHIM, with the aim of addressing key knowledge gaps in dNTS and iNTS disease pathogenesis and immunity. We will conduct a double-blind, randomised, safety and dose-escalation study with the primary objective of determining the infectious dose required for 60-75% of participants to develop systemic salmonellosis. Successfully establishing a reproducible NTS CHIM could provide valuable insights into mechanisms of protective immunity and serve as a platform for future evaluation of candidate iNTS vaccines and therapeutics.

4 METHODS AND ANALYSIS

4.1 Study design

The CHANTS study is a double-blinded, randomised, quarantine-based, controlled human infection model which aims to establish the infectious dose required for healthy participants to develop salmonellosis following oral challenge with either *S.* Typhimurium ST313 (strain D23580) or ST19 (strain 4/74). A predicted total of 40-80 healthy adult volunteers who are eligible, and consenting, will be randomised 1:1 to receive oral challenge with either D23580 or 4/74. The study will be conducted at Imperial College London with clinical support from partner tertiary hospitals at Imperial College Healthcare NHS Trust. Participant screening and recruitment commenced in August 2023, and the study is anticipated to conclude in Summer 2025.

4.2 Patient and public involvement

For peer review only - http://bmjopen.bmj.com/site/about/guidelines.xhtml

For peer review only

Patient and public engagement activities have been conducted throughout the protocol design stage, gaining valuable insights from the involvement of key stakeholders. Focus groups have been conducted with support of the NIHR Imperial Biomedical Research Centre patient experience research group. Discussions with members of the public in this forum – including past participants of previous CHIMs – have identified key considerations for the safe and acceptable conduct of this trial and supported the development of clear and thorough participant-facing information to underpin informed consent. Future similar activities are planned in regions with high iNTS burden to establish acceptability of potential future transfer of an iNTS CHIM to settings most affected by the disease under investigation.

4.3 Study objectives and outcomes

The primary objective is to determine the minimum infectious dose in colony forming units (CFU) required for 60-75% of healthy participants to develop clinical or microbiological features of systemic salmonellosis following oral challenge with either *S.* Typhimurium D23580 or 4/74. Systemic salmonellosis is defined as the development of either: (1) fever ≥38°C on ≥2 occasions ≥12 hours apart, or (2) *S.* Typhimurium bacteraemia at any time point. As outlined above, exposure to *S.* Typhimurium can result in heterogeneous disease phenotypes, ranging from asymptomatic gastrointestinal colonisation to bloodstream infection. Secondary endpoints include determining the proportion of participants who develop outcomes along this disease spectrum, including *Salmonella* colonisation, diarrhoeal disease (dNTS), systemic symptoms, or severe salmonellosis. Secondary study outcomes also aim to describe the immune response to *S.* Typhimurium infection (Table 1: Primary and secondary study objectives and outcomes).

In this first-in-human study, we have a unique opportunity to elucidate mechanisms of bacterial virulence in dNTS and iNTS, identify immune correlates of protection, describe host-pathogen interactions, changes in host microbiota in response to infection, and develop novel diagnostic platforms (Supplementary Table 1).

4.4 Recruitment and eligibility

Several strategies will be employed to recruit study participants, including advertising throughout local hospitals, GP surgeries, and higher education institutions; media and website advertising; direct email to members of the NIHR Imperial Clinical Research Facility healthy volunteer database; direct

For peer review only - http://bmjopen.bmj.com/site/about/guidelines.xhtml

mail-out and email to potentially eligible participants identified via National Health Applications and Infrastructure Services (NHAIS). Consenting healthy adult males and non-pregnant females aged 18-50 without pre-existing risk factors for severe iNTS or chronic infection will be recruited. Strict eligibility criteria have been designed to mitigate risks of severe disease, chronic carriage, or development of focal seeded infection. Detailed screening will include comprehensive medical history, physical examination, baseline blood counts, biochemical profiles, screening for immunosuppression, and abdominal ultrasonography to exclude subclinical biliary or abdominal aortic disease (see supplementary Table 2 for full eligibility criteria). Individuals who have had documented prior *Salmonella* infection, or received live-attenuated oral Ty21a typhoid vaccine, will be excluded due to potential cross-protective immune responses against *S*. Typhimurium. Further temporary exclusion criteria will be reviewed prior to oral challenge to exclude intercurrent infection or antimicrobial use which may bias results (Supplementary Table 3).

4.5 Study interventions

Consenting participants who fulfil eligibility criteria will enter the study at Day -7 (7 days prior to oral bacterial challenge). Days 0-7 will take place under clinical observation in a quarantine research facility at Imperial College Healthcare NHS Trust, with subsequent daily out-patient reviews on Days 8-14, prior to long-term follow-up visits at Days 28, 90, 180, and 365. The overall participant journey is illustrated in Figure 1.

4.5.1 Challenge Strain Selection and Manufacture

Challenge strains have been selected to maximise future utility of the model in supporting iNTS vaccine development. Wild-type ST313 (D23580) and ST19 (4/74) are phylogenetically representative of contemporary disease-causing isolates, have a traceable history from isolation, and have undergone detailed genotypic, phenotypic, and transcriptomic characterisation [19,20]. Bacterial stocks have been manufactured to GMP standard by the Walter Reed Army Institute of Research, MD, USA, in collaboration with PATH (Seattle, USA), with strains originally drawn from the UKHSA National Collection of Type Cultures.

ST313 lineage 2 reference strain D23580 was originally isolated from a blood culture obtained from a 24-month-old HIV-negative child at Queen Elizabeth Central Hospital, Blantyre, Malawi, and is

For peer review only - http://bmjopen.bmj.com/site/about/guidelines.xhtml

considered an archetypal isolate representative of strains causing iNTS in SSA. ST19 (4/74), originally isolated from a calf in England in 1974, is considered an archetypal strain causative of dNTS and representative of current clinical infections. D23580 harbours a distinct prophage and plasmid repertoire compared with 4/74, including a virulence plasmid pSLT encoding resistance to ampicillin, chloramphenicol, streptomycin, sulphonamides, and trimethoprim [20]. Both strains are susceptible to ciprofloxacin, azithromycin and third generation cephalosporins, ensuring a range of treatment options are available for challenged participants. The rationale for strains selected for use in this model, and their relative merits, have been discussed in detail elsewhere [21]. We hypothesise that ST313 (D23580) will be associated with an invasive phenotype compared with the enterocolitis anticipated as a result of challenge with ST19 (4/74).

4.5.2 Randomisation and Blinding

Block randomisation (block size = 10) to strain allocation will be carried out on or after Day -7. An initial sentinel cohort (n = 10) of will be randomised 1:1 (block sizes = 2, 4, 4) to receive a starting dose 1-5 x 10^3 CFU of either strain 4/74 or D23580. This starting dose has been determined based on prior experience from typhoidal CHIMs and continual reassessment method (CRM) dose prediction simulations (section 4.8) [15,22–24]. Unblinded attack rates and safety data will be reviewed by the Data Safety and Monitoring Committee (DSMC) prior to further participant randomisation (section 4.6). Randomisation lists will be generated and stored on a secure server accessible only by unblinded study team members. All clinical staff and participants will remain blinded to challenge allocation for the duration of the study.

4.5.3 Dose Escalation/De-escalation

Dose escalation and de-escalation decisions will be undertaken by the continual reassessment method (CRM). Escalation for both strains will occur in parallel, starting at 1-5 x 10^3 CFU, increasing to a maximum of 1-5 x 10^6 CFU or decreasing down to 1-5 x 10^1 CFU. Participants will be randomised to receive one of the two challenge agents at the dose determined by the CRM until target dosing is established for one agent. From that point, the remaining participants will be challenged with the other agent. We aim to recruit between 40-80 participants in total (20-40 for each challenge agent). The final challenge dose will be defined as one that achieves a 60-75% attack rate. The DSMC will be provided with an updated report after each cohort of 5 completes challenge. The dose escalation/de-escalation decision algorithm is illustrated in Figure 2.

For peer review only - http://bmjopen.bmj.com/site/about/guidelines.xhtml

4.5.4 Challenge procedures

Participants will receive oral bacterial challenge with either D23580 or 4/74 on study Day 0, following admission to an isolation side-room within the quarantine facility. Assessments will be conducted to confirm the absence of any temporary exclusion criteria (see supplementary Table 3) prior to challenge. Female participants will also undergo pregnancy testing both at screening and immediately prior to challenge. Both *S.* Typhimurium challenge agents used in this study will be administered via the oral route with sodium bicarbonate buffer to achieve gastric acid neutralisation. Participants will fast for 90 minutes before and after challenge. The solution for ingestion containing either wild type S. Typhimurium 4/74 or D23580 will be prepared in a Class II biological safety cabinet that is solely used for the purpose of preparing the challenge solution.

4.5.5 Clinical Assessment During Quarantine

Participants will be reviewed daily during admission to the quarantine facility by a clinical member of the study team. Extensive assessments will be undertaken, including physical examination, recording vital signs, assessment of mood, and review of participant symptom diaries. Blood, stool, and saliva specimen collection will be undertaken according to timelines outlined in supplementary table 4. Supportive therapy will be provided as required for symptom control e.g., oral rehydration solution or intravenous fluids, analgesia, and anti-emetics.

4.5.6 Infection Prevention and Control

Participants will be managed using infection control protocols appropriate for enteric infections. Study investigators will adhere to strict hand-hygiene policies of the research facility and utilise enteric personal protective equipment (PPE) for all study procedures. Participants will receive clear instruction on hand hygiene procedures – including illustrated guidance – to be adhered to during and after quarantine, in addition to guidance on cleaning protocols for bathrooms in the home environment following discharge. Participants will be nursed within single-occupancy side-rooms with ensuite toilet facilities for the duration of quarantine. The near-patient environment, including door handles, taps, toilet flushes and bed rails will be decontaminated daily throughout the quarantine period in accordance with local policies. Assessment of *Salmonella* environmental contamination

For peer review only - http://bmjopen.bmj.com/site/about/guidelines.xhtml

within the treatment facility will be undertaken as an exploratory study objective, utilising culture, and molecular methods to detect the presence of *S.* Typhimurium in the participant's immediate surroundings and in high traffic clinical areas.

Close contacts of participants, including household contacts, will be offered the opportunity to be screened for *Salmonella* infection, which will involve obtaining three stool samples 48-hours apart a minimum of seven days after the participant has begun antibiotic treatment. If either stool culture of a household contact is positive, he/she will be referred to a Consultant in Infectious Diseases for appropriate antibiotic management and the local health protection unit will be informed.

4.5.7 Antimicrobial therapy

Treatment of *Salmonella* infection in healthy immunocompetent adults poses a number of specific challenges. Antimicrobial therapy is indicated for invasive disease but may prolong shedding in patients with pauci-symptomatic gastroenteritis. We have elected to offer rescue treatment in specific circumstances. The rationale for this decision is discussed elsewhere [21]. Antibiotic therapy will be initiated in all participants who fulfil criteria for *Salmonella* diagnosis (SD) through reaching either or both of the defined primary endpoints of persistent fever or bloodstream infection. Treatment will also be commenced in participants who do not achieve the primary outcome measures but reach any of the other pre-specified treatment criteria outlined in Table 2. Treatment for bloodstream infection will comprise oral ciprofloxacin 500mg twice daily for 10-14 days, or second line therapy of intravenous ceftriaxone 1g once daily for up to 14 days. All other treatment indications (systemic illness, gastroenteritis, asymptomatic/pauci-symptomatic shedding) will be treated with oral ciprofloxacin 500mg twice daily for 5 days, or with second-line oral azithromycin 500mg once daily for 5 days. Antibiotic treatment will not be routinely offered to participants who do not fulfil any pre-specified treatment criteria. The full antibiotic treatment decision algorithm is illustrated in supplementary figure 1.

4.5.8 Discharge from Quarantine

The duration of quarantine will be 7 days from the time of oral challenge (168 hours). Participants will be eligible for discharge if they fulfil the criteria outlined in Table 3. Some participants may be discharged earlier than day 7 if they have been started on antibiotic therapy and completed 96 hours

For peer review only - http://bmjopen.bmj.com/site/about/guidelines.xhtml

of follow-up after starting treatment. If these criteria are not met on day 7, then participants will be asked to remain in quarantine until discharge criteria are met. The discharge decision making algorithm is illustrated in supplementary figure 2.

Discharge criteria have been designed to maximise participant clinical safety in addition to minimising risk of secondary onward transmission. Current UKHSA guidance does not mandate the collection of clearance stool cultures following an episode of gastroenteritis secondary to NTS infection. Instead, the absence of diarrhoea for at least 48 hours is considered sufficient for release from isolation [25]. In this study, clearance stool cultures will be obtained to meet a secondary study objective of measuring predicted differences in patterns of shedding between 4/74 and D23580 strains.

4.6 Governance

An independent Data and Safety Monitoring Committee (DSMC) has been appointed to provide real-time oversight of safety and trial conduct. The DSMC will review unblinded safety data throughout the study according to a pre-defined DSMC charter. The committee will have access to data and, if required, will monitor these data, and make recommendations to the study investigators on whether there are any ethical or safety reasons the trial should not continue. The DSMC will review the attack rate and advise on dose escalation/de-escalation decisions. Study monitoring will also be undertaken by a Contract Research Organisation (CRO), separate to and independent from the DSMC, to ensure safety and compliance with the approved study protocol. Strict data management plans will be adhered to with compliance to Good Clinical Practice guidelines.

4.7 Safety

As with all controlled human infection models, maintenance of participant safety during and after the study is paramount. In this model, general risks associated with participation may include study-fatigue, repeated phlebotomy procedures, and consequences of isolation in quarantine. Risks more directly attributable to challenge agents include symptomatic infection, complications of antibiotic treatment (in the case of bacterial challenge), and the small risk of long-term sequelae. Potential risks have been given extensive consideration, with risk mitigation strategies devised to minimise possible impact on participants. The potential complications of participation – and risk mitigation strategies – have been discussed elsewhere [21]. In addition to participant safety, careful consideration has been

For peer review only - http://bmjopen.bmj.com/site/about/guidelines.xhtml

given to ensure safety of study investigators and close contacts. Clear infection control protocols have been developed (as outlined above), in addition to close liaison with UKHSA to monitor and address any potential secondary transmission events, including within households following discharge from the quarantine facility.

4.8 Statistical analysis

This dose-finding study will use the continual reassessment method (CRM) to determine the target dose that can achieve a 60-75% attack rate [26,27]. To our knowledge, this will be the first dose-finding CHIM to employ CRM methodology. The advantages over traditional rule-based dose-finding studies are that the CRM method borrows information across all dose levels, which increases likelihood of finding the true dose for a given sample size compared with rule-based methods in the majority of scenarios, particularly when the maximum sample size is small. There is no formal sample size calculation for this study. The maximum sample size will be 40 participants for each challenge strain. The operating characteristics of the CRM and the rule-based approach are illustrated in supplementary Table 5. An ICMJE data availability statement is included in supplementary Table 6, applicable after conclusion of the study.

4.9 Limitations

The primary limitations of the CHANTS study – shared with most controlled human infection models – stem from undertaking the study in a healthy adult volunteer group which may not accurately reflect natural disease acquisition and pathogenesis. There are clear host factor differences between this group and individuals most susceptible to iNTS disease. However, as with prior CHIMs undertaken in healthy participants, we anticipate this infection model will generate key insights into *Salmonella* infection biology with future translational applications. Successful development of an *S.* Typhimurium CHIM, with an established safety profile and infectious dose, could be transferred to an endemic setting for future investigation in a population more closely resembling those at greatest risk of disease, as has been achieved previously in a range of enteric and non-enteric pathogen challenge models [28–32].

In addition to inherent differences between our study population and those at greatest risk of iNTS, there are limitations in achievable study endpoints. Our primary endpoint of persistent fever, with or without bloodstream infection, does not necessarily reflect the severe clinical phenotypes an iNTS

For peer review only - http://bmjopen.bmj.com/site/about/guidelines.xhtml

vaccine would aim to protect against in field settings. However, in view of the heterogeneous clinical phenotypes associated with NTS infection, ranging from asymptomatic colonisation through to septic shock, this endpoint is felt to represent the optimal trade-off between clinical manifestations of disease and patient safety within the context of a CHIM. The relative merits of endpoint selection and their rationale have been discussed previously [21].

5 ETHICS AND DISSEMINATION

This study protocol has been reviewed and approved by the NHS Health Research Authority (London – Fulham Research Ethics Committee 21/PR/0051; IRAS 301659) and is registered on clinicaltrials.gov (registration number NCT05870150). The sponsor is Imperial College London, and the study is indemnified under institutional and NHS insurance policies. Our findings will be of significant international interest, with several potential future applications for assessment of iNTS vaccines and novel therapeutics. We will publish all findings in peer-reviewed journals, and ensure presentation at national and international conferences, in addition to close liaison with key stakeholders, participants and funders.

For peer review only - http://bmjopen.bmj.com/site/about/guidelines.xhtml

6 REFERENCES

[1] Roth GA, Abate D, Abate KH, Abay SM, Abbafati C, Abbasi N, et al. Global, regional, and national age-sex-specific mortality for 282 causes of death in 195 countries and territories, 1980–2017: a systematic analysis for the Global Burden of Disease Study 2017. The Lancet 2018;392:1736–88. https://doi.org/10.1016/S0140-6736(18)32203-7.

[2] Feasey NA, Dougan G, Kingsley RA, Heyderman RS, Gordon MA. Invasive non-typhoidal salmonella disease: an emerging and neglected tropical disease in Africa. Lancet 2012;379:2489–99. https://doi.org/10.1016/S0140-6736(11)61752-2.

[3] GBD 2017 Non-Typhoidal Salmonella Invasive Disease Collaborators. The global burden of non-typhoidal salmonella invasive disease: a systematic analysis for the Global Burden of Disease Study 2017. Lancet Infect Dis 2019;19:1312–24. https://doi.org/10.1016/S1473-3099(19)30418-9.

[4] Feasey NA, Everett D, Faragher EB, Roca-Feltrer A, Kang'ombe A, Denis B, et al. Modelling the Contributions of Malaria, HIV, Malnutrition and Rainfall to the Decline in Paediatric Invasive Non-typhoidal Salmonella Disease in Malawi. PLoS Negl Trop Dis 2015;9:e0003979. https://doi.org/10.1371/journal.pntd.0003979.

[5] Tacconelli E, Carrara E, Savoldi A, Harbarth S, Mendelson M, Monnet DL, et al. Discovery, research, and development of new antibiotics: the WHO priority list of antibiotic-resistant bacteria and tuberculosis. Lancet Infect Dis 2018;18:318–27. https://doi.org/10.1016/S1473-3099(17)30753-3.

[6] Uche IV, MacLennan CA, Saul A. A Systematic Review of the Incidence, Risk Factors and Case Fatality Rates of Invasive Nontyphoidal Salmonella (iNTS) Disease in Africa (1966 to 2014). PLoS Negl Trop Dis 2017;11:e0005118. https://doi.org/10.1371/journal.pntd.0005118.

[7] Ao TT, Feasey NA, Gordon MA, Keddy KH, Angulo FJ, Crump JA. Global burden of invasive nontyphoidal Salmonella disease, 2010(1). Emerg Infect Dis 2015;21. https://doi.org/10.3201/eid2106.140999.

[8] Marchello CS, Fiorino F, Pettini E, Crump JA, Vacc-iNTS Consortium Collaborators. Incidence of non-typhoidal Salmonella invasive disease: A systematic review and meta-analysis. J Infect 2021;83:523–32. https://doi.org/10.1016/j.jinf.2021.06.029.

[9] Pulford CV, Perez-Sepulveda BM, Canals R, Bevington JA, Bengtsson RJ, Wenner N, et al. Stepwise evolution of Salmonella Typhimurium ST313 causing bloodstream infection in Africa. Nat Microbiol 2021;6:327–38. https://doi.org/10.1038/s41564-020-00836-1.

[10] Read the report : Vaccines for AMR n.d. https://vaccinesforamr.org/read-the-report/ (accessed April 27, 2023).

[11] Baliban SM, Lu Y-J, Malley R. Overview of the Nontyphoidal and Paratyphoidal Salmonella Vaccine Pipeline: Current Status and Future Prospects. Clin Infect Dis 2020;71:S151–4. https://doi.org/10.1093/cid/ciaa514.

[12] Choy RKM, Bourgeois AL, Ockenhouse CF, Walker RI, Sheets RL, Flores J. Controlled Human Infection Models To Accelerate Vaccine Development. Clin Microbiol Rev 2022;35:e0000821. https://doi.org/10.1128/cmr.00008-21.

[13] Roestenberg M, Hoogerwerf M-A, Ferreira DM, Mordmüller B, Yazdanbakhsh M. Experimental infection of human volunteers. Lancet Infect Dis 2018;18:e312–22. https://doi.org/10.1016/S1473-3099(18)30177-4.

[14] Chen WH, Cohen MB, Kirkpatrick BD, Brady RC, Galloway D, Gurwith M, et al. Single-dose Live Oral Cholera Vaccine CVD 103-HgR Protects Against Human Experimental Infection With Vibrio cholerae O1 El Tor. Clin Infect Dis 2016;62:1329–35. https://doi.org/10.1093/cid/ciw145.

[15] Jin C, Gibani MM, Moore M, Juel HB, Jones E, Meiring J, et al. Efficacy and immunogenicity of a Vi-tetanus toxoid conjugate vaccine in the prevention of typhoid fever using a controlled human

For peer review only - http://bmjopen.bmj.com/site/about/guidelines.xhtml

infection model of Salmonella Typhi: a randomised controlled, phase 2b trial. The Lancet 2017;390:2472–80. https://doi.org/10.1016/S0140-6736(17)32149-9.

[16] Shakya M, Colin-Jones R, Theiss-Nyland K, Voysey M, Pant D, Smith N, et al. Phase 3 Efficacy Analysis of a Typhoid Conjugate Vaccine Trial in Nepal. N Engl J Med 2019;381:2209–18. https://doi.org/10.1056/NEJMoa1905047.

[17] Patel PD, Patel P, Liang Y, Meiring JE, Misiri T, Mwakiseghile F, et al. Safety and Efficacy of a Typhoid Conjugate Vaccine in Malawian Children. N Engl J Med 2021;385:1104–15. https://doi.org/10.1056/NEJMoa2035916.

[18] Qadri F, Khanam F, Liu X, Theiss-Nyland K, Biswas PK, Bhuiyan AI, et al. Protection by vaccination of children against typhoid fever with a Vi-tetanus toxoid conjugate vaccine in urban Bangladesh: a cluster-randomised trial. The Lancet 2021;398:675–84. https://doi.org/10.1016/S0140-6736(21)01124-7.

[19] Kröger C, Colgan A, Srikumar S, Händler K, Sivasankaran SK, Hammarlöf DL, et al. An infection-relevant transcriptomic compendium for Salmonella enterica Serovar Typhimurium. Cell Host Microbe 2013;14:683–95. https://doi.org/10.1016/j.chom.2013.11.010.

[20] Canals R, Hammarlöf DL, Kröger C, Owen SV, Fong WY, Lacharme-Lora L, et al. Adding function to the genome of African Salmonella Typhimurium ST313 strain D23580. PLoS Biol 2019;17:e3000059. https://doi.org/10.1371/journal.pbio.3000059.

[21] Smith C, Smith E, Chiu C, Hinton J, Perez Sepulveda B, Gordon M, et al. The Challenge Non-Typhoidal Salmonella (CHANTS) Consortium: Development of a non-typhoidal Salmonella controlled human infection model: Report from a consultation group workshop, 05 July 2022, London, UK. Wellcome Open Res 2023;8:111. https://doi.org/10.12688/wellcomeopenres.19012.1.

[22] Gibani MM, Jin C, Shrestha S, Moore M, Norman L, Voysey M, et al. Homologous and heterologous re-challenge with Salmonella Typhi and Salmonella Paratyphi A in a randomised controlled human infection model. PLoS Negl Trop Dis 2020;14:e0008783. https://doi.org/10.1371/journal.pntd.0008783.

[23] Gibani MM, Jones E, Barton A, Jin C, Meek J, Camara S, et al. Investigation of the role of typhoid toxin in acute typhoid fever in a human challenge model. Nat Med 2019;25:1082–8. https://doi.org/10.1038/s41591-019-0505-4.

[24] Dobinson HC, Gibani MM, Jones C, Thomaides-Brears HB, Voysey M, Darton TC, et al. Evaluation of the Clinical and Microbiological Response to Salmonella Paratyphi A Infection in the First Paratyphoid Human Challenge Model. Clinical Infectious Diseases 2017;64:1066–73. https://doi.org/10.1093/cid/cix042.

[25] Recommendations for the Public Health Management of Gastrointestinal Infections 2019 2020.

[26] O'Quigley J, Pepe M, Fisher L. Continual reassessment method: a practical design for phase 1 clinical trials in cancer. Biometrics 1990;46:33–48.

[27] Wheeler GM, Mander AP, Bedding A, Brock K, Cornelius V, Grieve AP, et al. How to design a dose-finding study using the continual reassessment method. BMC Med Res Methodol 2019;19:18. https://doi.org/10.1186/s12874-018-0638-z.

[28] Morton B, Jambo K, Chikaonda T, Rylance J, Henrion MYR, Banda NP, et al. The influence of pneumococcal conjugate vaccine-13 on nasal colonisation in a controlled human infection model of pneumococcal carriage in Malawi: a double-blinded randomised controlled trial protocol. Wellcome Open Res 2021;6:240. https://doi.org/10.12688/wellcomeopenres.17172.2.

[29] Kapulu MC, Kimani D, Njuguna P, Hamaluba M, Otieno E, Kimathi R, et al. Controlled human malaria infection (CHMI) outcomes in Kenyan adults is associated with prior history of malaria exposure and anti-schizont antibody response. BMC Infect Dis 2022;22:86. https://doi.org/10.1186/s12879-022-07044-8.

[30] Pitisuttithum P, Cohen MB, Phonrat B, Suthisarnsuntorn U, Bussaratid V, Desakorn V, et al. A human volunteer challenge model using frozen bacteria of the new epidemic serotype, V.

For peer review only - http://bmjopen.bmj.com/site/about/guidelines.xhtml

cholerae O139 in Thai volunteers. Vaccine 2001;20:920–5. https://doi.org/10.1016/s0264-410x(01)00381-4.

[31] Pitisuttithum P, Islam D, Chamnanchanunt S, Ruamsap N, Khantapura P, Kaewkungwal J, et al. Clinical Trial of an Oral Live Shigella sonnei Vaccine Candidate, WRSS1, in Thai Adults. Clin Vaccine Immunol 2016;23:564–75. https://doi.org/10.1128/CVI.00665-15.

[32] Bodhidatta L, Pitisuttithum P, Chamnanchanant S, Chang KT, Islam D, Bussaratid V, et al. Establishment of a Shigella sonnei human challenge model in Thailand. Vaccine 2012;30:7040–5. https://doi.org/10.1016/j.vaccine.2012.09.061.

Authors Contributions

Conceptualisation and study design: CS, ES, AR, RV, JCDH, MAG, RKMC, XL, AJP, CC, GSC, MMG

Data analysis plan: CS, XL, GSC, MMG

Drafting of protocol: CS, ES, AR, RV, GSC, MMG

Protocol amendments: CS, RKMC, AJP, XL, MMG

Data Availability Statement

As this manuscript describes a study protocol, no datasets are available as yet.

Funding

The CHANTS study is funded by The Wellcome Trust (grant number 224029/Z/21/Z: awarded to principal investigator MMG). CS, GSC, CC and MMG are supported, in part, by the National Institute of Health Research Imperial Biomedical Research Centre.

Disclaimer

The funder of the study has had no role in study design, and will have no role in data collection, data analysis, data interpretation, writing of the report or decision to submit.

Competing Interests

None declared.

Word Count

Main Text: 3372

For peer review only - http://bmjopen.bmj.com/site/about/guidelines.xhtml

548 **Table 1**: Primary and secondary study objectives and outcomes

	Objective(s)	Outcome(s)/Endpoint(s)
**Primary**	Determine the minimum infectious dose (CFU) of *S.* Typhimurium required for 60-75% of volunteers to develop systemic Salmonellosis following oral challenge.	The proportion of participants who develop:<br>1. Fever ≥38°C on ≥2 occasions ≥12 hours apart **and/or**<br>2. *Salmonella* Typhimurium bacteraemia at any time point
**Secondary**	Describe colonisation rates following oral challenge at different doses.	The proportion from whom *Salmonella* Typhimurium is isolated from stool on ≥2 occasions ≥48 hours from challenge at different doses of each strain.
	Describe gastroenteritis rates following oral challenge at different doses.	The proportion of participants at different doses of each strain developing:<br>1.1 Severe diarrhoea* **and/or**<br>1.2 Moderate diarrhoea** **plus** Fever ≥38°C on ≥1 occasion **and/or** ≥1 Grade 2 gastrointestinal symptoms (abdominal pain, nausea, vomiting, tenesmus)
	Determine persistent fever rates following oral challenge at different doses.	The proportion of participants who develop fever ≥38°C on ≥2 occasions ≥12 hours apart at different doses of each strain.
	Describe bacteraemia rate following oral challenge at different doses.	The proportion of participants developing *Salmonella* Typhimurium bacteraemia at any timepoint following challenge at different doses of each strain.
	Describe the rates of systemic *Salmonellosis* according to **an alternative composite diagnostic criterion** following oral challenge at different doses.	The proportion of participants meeting the criteria for a composite diagnosis of *Salmonellosis* at different doses of each strain defined as any of:<br>1 *S.* Typhimurium isolated from stool on ≥2 occasions ≥48 hours from challenge **and/or**<br>2 Gastroenteritis **and/or**<br>3 Fever ≥38°C on ≥2 occasions ≥12 hours apart **and/or**<br>4 *S.* Typhimurium bacteraemia
	Describe the safety of oral challenge with *S.* Typhimurium 4/74 and D23580 strains.	The proportion of participants at different doses of each strain reporting: adverse events, adverse events of special interest, SAEs, SUSARs, concomitant medication usage.
	Compare **clinical** features following oral challenge with *S.* Typhimurium strains 4/74 or D23580.	A comparison of the clinical features of *Salmonella* infection after challenge, with specific reference to the proportion in each group developing:<br>1 Diarrhoea (volume, frequency, consistency, gastrointestinal symptom severity score, time to onset)<br>2 Any fever ≥38°C (including fever clearance time)<br>3 Solicited symptoms at any time: headache, malaise, anorexia, abdominal pain, nausea, vomiting, dysentery, myalgia, arthralgia, cough, rash (including measurement of severity, and duration from onset to resolution)

For peer review only - http://bmjopen.bmj.com/site/about/guidelines.xhtml

	Compare **microbiological** features of **gastrointestinal infection** following oral challenge with *S.* Typhimurium 4/74 or D23580.	Gastrointestinal *Salmonella* infection after challenge with 4/74 or D23580 strains, with specific reference to:<br>1 Time to onset in days in each group from challenge to first stool sample positive for *Salmonella* Typhimurium by culture and/or PCR<br>2 Duration of stool shedding.<br>3 The magnitude of stool shedding measured in CFU/ml in quantitative stool culture analysis
	Compare **microbiological** features of **bloodstream infection** following oral challenge with *S.* Typhimurium 4/74 or *S.* Typhimurium D23580 strains	*Salmonella* bloodstream infection after challenge, with specific reference to:<br>1 The proportion of participants in each group developing any bacteraemia<br>2 The severity of bacteraemia in each group as measured by time to onset, duration, clearance time, magnitude quantified in CFU/ml.
	Compare biochemical and haematological laboratory parameters following oral challenge.	Absolute values of laboratory parameters from time of challenge to Day 28 and/or Day 90, with specific reference to: Haemoglobin, white cell count and differential, urea and electrolytes, liver function tests.
	Describe and compare the serum antibody response following oral challenge with *S.* Typhimurium 4/74 or D23580 strains.	Serum samples measured at baseline and post challenge time points:<br>1 *S.* Typhimurium O-specific polysaccharide serum IgG and IgA concentration measured by ELISA<br>2 *S.* Typhimurium specific serum IgG and IgA concentration against other S. Typhimurium antigens measured by ELISA<br>3 Serum bactericidal antibody titres against *Salmonella* Typhimurium<br>4 *S.* Typhimurium specific antibody secreting cell and memory B-cell responses measured by ELISPOT<br>5 Other functional antibody activity measurements including systems serology platforms.
	Describe and compare the mucosal antibody response following oral challenge with *S.* Typhimurium 4/74 or D23580 strains.	Saliva and/or stool samples measured at baseline and post challenge time points:<br>1 S. Typhimurium specific IgG and IgA concentration measured by ELISA<br>2 Bactericidal antibody titres<br>3 Other functional antibody activity measurements including systems serology platforms
	Describe and compare cell-mediated immune response following oral challenge with *S.* Typhimurium 4/74 D23580 strains.	Absolute values and inter-group comparison of laboratory assays performed on peripheral blood mononuclear cells measured at baseline and post challenge time points:<br>1 Lymphocyte populations as measured by flow cytometry and/or CyTOF<br>2 *S.* Typhimurium specific cell-mediated immune responses as measured by ELISPOT and flow cytometry and/or CyTOF
	To describe participant experience following oral challenge with *S.* Typhimurium 4/74 or D23580 strains	Descriptive statistics following administration of a structured questionnaire at baseline and post-challenge.

*Severe gastroenteritis: ≥6 loose/liquid stools (Bristol Type 6-7) **and/or** >800 g of loose/liquid stools in a rolling 24-hour period **and/or** ≥2 stools with gross blood in 24 hours

**Moderate gastroenteritis: 4–5 loose/liquid stools (Bristol Type 6-7) and/or 400–800 g in a rolling 24-hour period

For peer review only - http://bmjopen.bmj.com/site/about/guidelines.xhtml

552 **Table 2: Criteria for commencing antibiotic therapy following oral bacterial challenge**

**Antibiotics are commenced if any of the following apply**
Any participant with *Salmonella* Typhimurium bacteraemia
Fever ≥38°C for ≥12hrs
Any participant with severe gastroenteritis*
Moderate gastroenteritis** plus:<br>• fever ≥38°C on one occasion and/or<br>• ≥1 Grade 2*** systemic symptoms
Any participant with 3 or more of the following symptoms on the same day at Grade 2 or higher.<br>• Headache<br>• Fatigue/Malaise<br>• Anorexia<br>• Abdominal pain<br>• Nausea<br>• Vomiting<br>• Myalgia<br>• Arthralgia<br>• Cough
Any participant from whom *Salmonella* has been detected from at least two stool culture/PCR and 24hrs apart who has not received antibiotics by day 14 post-challenge
Any participant in whom antibiotic use is felt to be clinically necessary (as decided by a medically qualified study doctor)
**Notes:**<br>*Severe gastroenteritis: ≥6 loose/liquid stools (Bristol Type 6-7) **and/or** >800 g of loose/liquid stools in a rolling 24-hour period **and/or** ≥2 stools with gross blood in 24 hours<br>**Moderate gastroenteritis: 4–5 loose/liquid stools (Bristol Type 6-7) and/or 400–800 g in a rolling 24-hour period<br>***Grade 0 = not present; Grade 1 = Present but no interference with activity; Grade 2 = Some interference with activity; Grade 3 = Significant; prevents daily activity; Grade 4: ED visit or hospitalisation

553

554

For peer review only - http://bmjopen.bmj.com/site/about/guidelines.xhtml

Table 3: Quarantine discharge criteria

**Participants will be discharged from the inpatient quarantine unit if the following criteria are met:**
• Medically fit for discharge in the opinion of the study physician **AND**
• Complete resolution of diarrhoea (Bristol stool type 6-7) for 48 hours **AND**
• Seven days (168 hours) have elapsed since challenge
**For patients diagnosed with Salmonellosis from day 0 to day 7, the following criteria apply:**
• Antibiotic treatment has been initiated and patient has completed 96hrs follow up
• Resolution of *Salmonella* Typhimurium bacteraemia (if applicable)

For peer review only

For peer review only - http://bmjopen.bmj.com/site/about/guidelines.xhtml

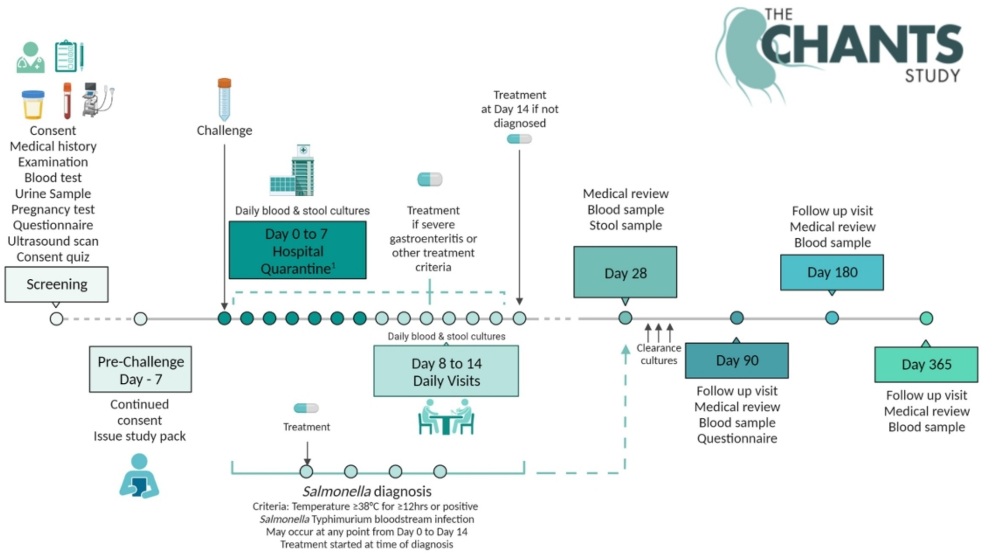

Overview of the participant journey from screening to study completion at 1 year of follow-up

269x159mm (220 x 220 DPI)

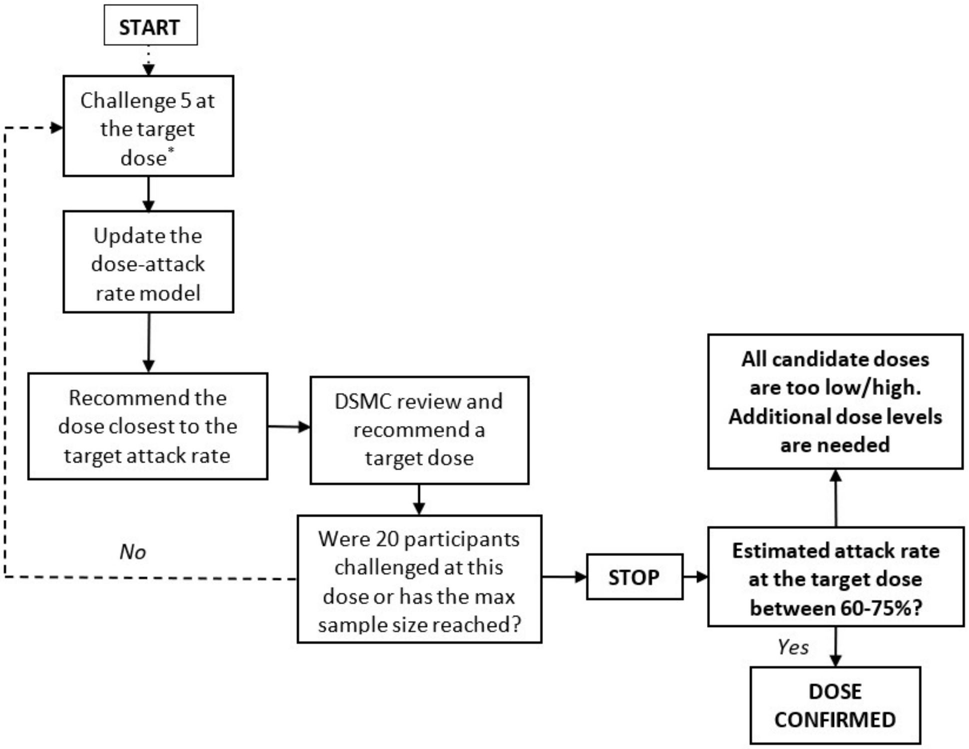

Decision making algorithm for dose escalation/de-escalation. Starting at 1-5 x10^3 CFU to reach the primary endpoint. *The target dose for the first cohort will be 1-5 x 10^3 CFU, and the first cohort of 5 participants will be divided into 3 groups (1, 2, and 2 participants) and challenged with an interval of at least 7 days. For the rest of the cohorts, they will be challenged on the same day, if possible.

149x117mm (144 x 144 DPI)

Supplementary Materials

Table 1: Exploratory Objectives

**Exploratory**	1	To investigate how the human microbiota interacts with oral challenge of *S.* Typhimurium 4/74 or D23580 strains	Metagenomic analysis of stool and/or saliva to measure constituent microbiological flora (and their metabolome/transcriptome) at baseline and post-challenge time points
	2	To investigate *Salmonella* Typhimurium virulence factors and gene expression	Exploratory analysis of stool samples at several time points following challenge to assess bacterial transcriptome by RNA-seq
	3	To describe and compare additional host immune responses following oral challenge of *S.* Typhimurium 4/74 or D23580 strains.	Analysis of B-cell repertoire, plasma cytokine profile and kinetics.
	4	To evaluate environmental contamination with *Salmonella enterica* in the near patient environment	The pattern and proportion of environmental samples testing positive for *Salmonella* Typhimurium by culture or molecular methods.
	5	To evaluate host-pathogen interactions following *Salmonella* infection including the presence and mechanisms of *Salmonella* persister infected macrophages.	Exploratory analysis of infected immune cells from blood including, but not limited to, single-cell RNA-seq.

For peer review only

Table 2: Eligibility criteria for participation

**Inclusion Criteria**
Agree to give informed consent for participation in the study.
Aged between 18 and 50 years inclusive at time of challenge.
In good health as determined by medical history, physical examination, and clinical judgment of the study team.
Agree (in the study team's opinion) to comply with all study requirements, including capacity to adhere to good personal hygiene and infection control precautions.
Agree to allow his or her General Practitioner (and/or Consultant if appropriate), to be notified of participation in the study.
Agree to allow study staff to contact his or her GP to access the participant's medical history and vaccination records.
Agree to allow UKHSA and the local health protection unit to be informed of their participation in the study.
Agree to give his or her close contacts written information informing them of the participant's involvement in the study and offer them voluntary screening for Salmonella carriage.
Agree to a period of inpatient quarantine whilst symptomatic (In exceptional circumstances, the duration of inpatient quarantine may be extended.)
Agree to have 24-hour contact with study staff during the four weeks post challenge and can ensure that they are contactable by mobile phone for the duration of the study period until antibiotic completion.
Agree to allow the study team to hold the name and 24-hour contact number of a close friend, relative or housemate who will be kept informed of the study participant's whereabouts for the duration of the challenge period after quarantine (from the time of challenge until completion of antibiotic course). This person will be contacted if study staff are unable to contact the participant after discharge from inpatient quarantine.
Have internet access to allow completion of the e-diary and real-time safety monitoring.
Agree to avoid antipyretic/anti-inflammatory treatment from the time of challenge (Day 0) until advised by a study doctor or until 14 days after challenge.
Agree to refrain from donating blood for the duration of the study.
Agree to provide their National Insurance/Passport number for the purposes of Trial over-volunteering prevention system (TOPS) registration and for payment of reimbursement expenses.
Participants must have received at least two doses of a SARS-CoV-2 vaccines that has been approved for use by the MHRA (or other national regulatory authority) four weeks prior to enrolment.
Proficient in English at a level sufficient to understand, retain, weigh up and communicate the study details as outlined in the participant information sheet, in the opinion of the study investigator.
**Exclusion Criteria**
History of microbiologically confirmed *Salmonella* infection[1].
History of receipt of oral Ty21a typhoid vaccination
History of **significant[2]** organ-specific and/or systemic disease that could interfere with trial conduct or completion. Including, for example, but not restricted to:<br>- Cardiovascular disease (ischaemic heart disease, valvular heart disease, vascular disease including aneurysmal disease or endovascular prostheses, prolonged corrected QT interval (>450 milliseconds) on ECG screening).

For peer review only

- Respiratory disease[3]<br>- Haematological disease including sickle cell disease and sickle cell trait.<br>- Endocrine disorders, including specifically diabetes mellitus.<br>- Renal or bladder disease, including history of renal calculi.<br>- Biliary tract disease, including specifically colic, asymptomatic gallstones, cholecystectomy.<br>- Gastro-intestinal disease including specifically: a current requirement for antacids, H_2-receptor antagonists, proton pump inhibitors or laxatives, Inflammatory bowel disease, confirmed diagnosis of irritable bowel syndrome as defined by the Rome IV criteria,<br>- Neurological disease<br>- Metabolic disease<br>- Autoimmune disease<br>- Psychiatric illness requiring hospitalisation.<br>- Known or suspected drug and/or alcohol misuse disorder<br>- Chronic/Active Infectious disease including active tuberculosis.<br>- Severe infection requiring hospitalisation for intravenous antibiotics within the last 10 years. Exceptions to this would include a short course of intravenous antibiotics for appendicitis, biliary sepsis, diverticulitis, and cellulitis.<br>- History of recent malaria infection in the past 12 months from screening<br>- History of joint replacement<br>- History of any orthopaedic/osseous implanted prosthesis<br>- Presence of any other internal implanted device e.g., permanent pacemaker.
Have any known or suspected impairment of immune function (as defined in the green book[140]), alteration of immune function, or prior immune exposure that may alter immune function to *Salmonella* infection resulting from, for example:<br>- Congenital or acquired immunodeficiency, including IgA deficiency.<br>- Human Immunodeficiency Virus infection or symptoms/signs suggestive of an HIV-associated condition<br>- Evidence of severe primary immunodeficiency, for example, severe combined immunodeficiency, Wiskott-Aldrich syndrome, and other combined immunodeficiency syndromes.<br>- Currently being treated for malignant disease with immunosuppressive chemotherapy or radiotherapy, or who have received such treatment within at least the last six months.<br>- Individuals who have received a solid organ transplant and are currently on immunosuppressive treatment.<br>- Individuals who have received a bone marrow transplant, until within 12 months of finishing all immunosuppressive treatment,<br>- Individuals receiving systemic high dose steroids[4] until at least three months after treatment has stopped.<br>- Individuals receiving other types of immunosuppressive[5] drugs (alone or in combination with lower doses of steroids) until at least six months after terminating such treatment.<br>- Receipt of immunoglobulin or any blood product transfusion within 3 months of study start.<br>- History of cancer (except squamous cell or basal cell carcinoma of the skin and cervical carcinoma in situ).
Moderate or severe depression or anxiety as classified by the Hospital Anxiety and Depression Score at screening or challenge that is deemed clinically significant by the study doctors[6].
Weight less than 50kg[7]
Anyone taking long-term medication (e.g., analgesia, anti-inflammatories, or antibiotics) that may affect symptom reporting or interpretation of the study results.
Contraindication to cephalosporin, fluroquinolone, or macrolide antibiotics.

For peer review only

Female participants who are pregnant, lactating or who are unwilling to ensure that they or their partner use effective contraception[8] 30 days prior to challenge and continue to do so until two negative stool samples, a minimum of 3 weeks after completion of antibiotic treatment, have been obtained.
Full-time, part-time, or voluntary occupations involving:<br>- Clinical healthcare work[9]<br>- Social work with direct contact with young children (defined as those attending pre-school groups or nursery or aged under 2 years), or other clinically vulnerable children, adolescents.<br>- Clinical or social work with direct contact with highly susceptible patients or persons in whom *Salmonella* infection would have particularly serious consequences (unless willing to avoid work until demonstrated not to be infected with *Salmonella* in accordance with guidance from Public Health England and willing to allow study staff to inform their employer).
Full time, part time or voluntary occupations involving:<br>- Commercial food handling (involving preparing or serving unwrapped foods not subjected to further heating)
Close household contact with:<br>- Children aged less than 2 years<br>- Any individual who is immunocompromised.<br>- Pregnant women<br>- Household contacts aged over 70 years
Scheduled elective surgery or other procedures requiring general anaesthesia during the study period.
Participants who have participated in another research study involving an investigational product that might affect risk of *Salmonella* infection or compromise the integrity of the study within the 30 days prior to enrolment (e.g. significant volumes of blood already taken in previous study)[10].
Detection of any abnormal results from screening investigations, unless deemed not clinically significant.
Screening blood test positive for HLA-B*27 Antigen
Any other social, psychological or health issues which, in the opinion of the study staff, may:<br>- Put the participant or their contacts at risk because of participation in the study.<br>- Adversely affect the interpretation of the primary endpoint data.<br>- Impair the participant's ability to participate in the study.
Having previously received any experimental *Salmonella* vaccine as part of a clinical trial
Have participated in previous *Salmonella* Typhi or Paratyphi challenge studies (with ingestion of challenge agent).
Any employee of the sponsor or research site personnel directly affiliated with this study or their immediate family members.
Inability to comply with any of the study requirements (at the discretion of the study staff).

[1] Including enteric fever (*Salmonella* Typhi and/or *Salmonella* Paratyphi infection)

[2] Individuals with specific medical co-morbidities (e.g., childhood asthma) will be considered for enrolment after review with the principal investigator and clinical oversight group, following discussion with their GP and/or consultant as appropriate. The rationale for enrolment in these cases will be recorded in the CRF.

[3] Participants with well controlled asthma may be considered for enrolment at the discretion of the study investigators and following consultation with their general practitioner.

[4] Defined as those receiving at least 40mg of prednisolone per day for more than one week or equivalent.

[5] Azathioprine, cyclosporin, methotrexate, cyclophosphamide, leflunomide and other cytokine inhibitors.

For peer review only

[6] If elevated scores are due to temporary significant life events, the questionnaire may be repeated after resolution of the event with a view to inclusion if normal.
[7] Or a Body Mass Index (BMI) that, in the opinion of the study doctors, may adversely impair the interpretation of the study results or affect the safe performance of any study procedures.
[8] All forms of contraception are considered effective with the exception of barrier methods and natural family planning. Accepted methods include hormonal contraceptive pills, intrauterine contraceptive devices/systems, long-acting injectable hormonal contraception, contraceptive implants.
[9] This includes medical, dental, nursing or midwifery students (or other allied-healthcare professionals) with direct patient contact. Such students who are not undertaking patient-facing rotations until demonstrated not to be infected with *Salmonella* species may be considered for enrolment.
[10] As assessed by both participant questioning and registration with The Over Volunteering Prevention System (TOPS) database.

Table 3: Temporary Exclusion Criteria at time of bacterial challenge

**Temporary Exclusion Criteria at Challenge**
Positive lateral flow test for SARS-CoV-2
Significant acute or acute-on-chronic infection within the previous 7 days or have experienced fever (>37.5°C) or subjective febrile symptoms within the previous 3 days
History of any antibiotic therapy during the previous 5 days
Any systemic corticosteroid (or equivalent) treatment in the previous 14 days, or for more than seven consecutive days within the past 3 months
Therapy with antacids, proton pump inhibitors or H2-receptor antagonists within 24 hours prior to challenge
Detection of gastrointestinal pathogens (other than *Salmonella*) in stool culture/PCR collected at the pre-challenge assessment (Day-7) including *Shigella* spp., *Campylobacter* spp., *E. Coli* O157, *Giardia* spp and *Cryptosporidium* spp, until two subsequent samples are negative. Detection of *Salmonella* spp. in stool prior to challenge (Day – 7) will result in exclusion from the study
Detection of extended spectrum beta lactamase producing organisms (ESBLs) or Carbapenem resistant organisms (CRO) until two subsequent samples are negative.

For peer review only

Table 4: Clinical sample collection plan for patients undergoing primary challenge. Values correspond to volume of blood collected in millilitres.

	Pre-challenge		Challenge period																				Follow up			
			Inpatient quarantine								Outpatient assessments							Post diagnosis/treatment								
	Screening	Day -7	Day 0	Day 1	Day 2	Day 3	Day 4	Day 5	Day 6	Day 7	Day 8	Day 9	Day 10	Day 11	Day 12	Day 13	Day 14	SD	SD +24h	SD +48h	SD +72h	SD +96h	Day 28	Day 90	Day 180	Day 365
Stool sample		x	x	x	x	x	x	x	x	x	x	x	x	x	x	x	x	x	x	x	x	x	x	x	x	x
Blood culture BACTEC			10	10	10	10	10	10	10	10	10	10	10	10	10	10	10	10	10	10	10	10				
FBC	1	1	1		1		1		1		1		1		1		1	1	1	1	1	1	1	1	1	
U&E/CRP/LFT	2	2	2		2		2		2		2		2		2		2	2	2	2	2	2	2	2	2	
Additional screening investigations*	10																									
Bacterial quantification																		10								
Serum sample			10							10							10	10					10	10	10	10
Plasma Sample			3							3							3	3					3			
PBMC			25				25			25							25	25					25	25	25	25
Functional genomics			3	3	3	3				3								3					3	3		
Whole blood PCR			10	10	10	10	10	10	10	10																
DNA sample (including epigenetics)			x																				x		x	
Saliva sample			x							x							x						x		x	

*HIV/HBV/HV serology, HLA-B27, Haemoglobinopathy screen, Coeliac serology, HbA1c

Table 5: Simulation results comparing CRM with rule-based model.

Operating characteristics for CRM-based approach under 5 following scenarios:

Scenario 1 target dose at dose 1 with attack rate: 0.675, 0.8, 0.9, 0.95.
Scenario 2 target dose at dose 2 with attack rate: 0.5, 0.675, 0.8, 0.9.
Scenario 3 target dose at dose 4 with attack rate: 0.3, 0.4, 0.5, 0.675.
Scenario 4 target dose beyond dose 4 with attack rate: 0.2, 0.3, 0.4, 0.5.
Scenario 5 target dose under dose 1 with attack rate: 0.8, 0.9, 0.95, 0.975.

	**CRM-based***	**Rule-based**
**Scenario 1**		
Median sample size (IQR)	20 (20-25)	20 (20-25)
Probability of choosing the dose		
First dose too high	17.2%	16.4%
1 (67.5%)	69.1%	58.4%
2 (80%)	13.7%	8.6%
3 (90%)		
4 (95%)		
Fail to find a dose		16.6%
**Scenario 2**		
Median sample size (IQR)	30 (25-35)	30 (25-35)
Probability of choosing the dose		
First dose too high	0.7%	0.8%
1 (50%)	20.6%	17.3%
2 (67.5%)	64.9%	49.4%
3 (80%)	13.7%	3.9%
4 (90%)	0.1%	
Failed to find a dose	N/A	28.6%
**Scenario 3**		
Median sample size	35 (35-40)	30 (20-40)
Probability of choosing the dose		
1 (30%)	0.02%	0.6%
2 (40%)	2.2%	4.4%
3 (50%)	34.1%	14.1%
4 (67.5%)	59.1%	11.0%
Last dose too low	4.4%	0%
Fail to find a dose	N/A	69.9%
**Scenario 4**		
Median sample size	35 (35-40)	30 (25-35)
Probability of choosing the dose		
1 (20%)	0%	0%
2 (30%)	0.1%	0.8%
3 (40%)	5.3%	3.1%
4 (50%)	46.0%	10.7%
Last dose too low	48.6%	

Fail to find a dose	N/A	85.4%
**Scenario 5**		
Median sample size	20 (20-20)	20 (20-20)
Probability of choosing the dose		
First dose too high	64.7%	63.2%
1 (80%)	35.0%	32.3%
2 (90%)	0.3%	0.1%
3 (95%)	0%	0%
4 (97.5%)	0%	0%
Fail to find a dose	N/A	4.4%

*Parameters for the CRM-based design:

- Prior attack rate at the four doses: 0.6, 0.75, 0.85, 0.9;
- The attack rate is 67.5%;
- Starting at dose 1

Table 6 – ICMJE Data Availability Statement

**Which data in particular will be shared?**	**Are individual deidentified participant data (including data dictionaries) available?**	**Are additional, related documents available (e.g., study protocol, statistical analysis plan, etc.)?**	**When will the data become available and for how long?**	**By what access criteria will the data be shared (including with whom, for what types of analyses, and by what mechanism)?**
Individual participant data that underlie the results reported in the trial, after deidentification	Yes, pertaining to established endpoints of the trial	Study protocol	Following publication of the trial, ending 36 months from publication date	Investigators whose proposed use of data has been approved by an independent ethics review committee, identified for this purpose

For peer review only

Figure 1: Antibiotic Treatment Algorithm

CHANTS

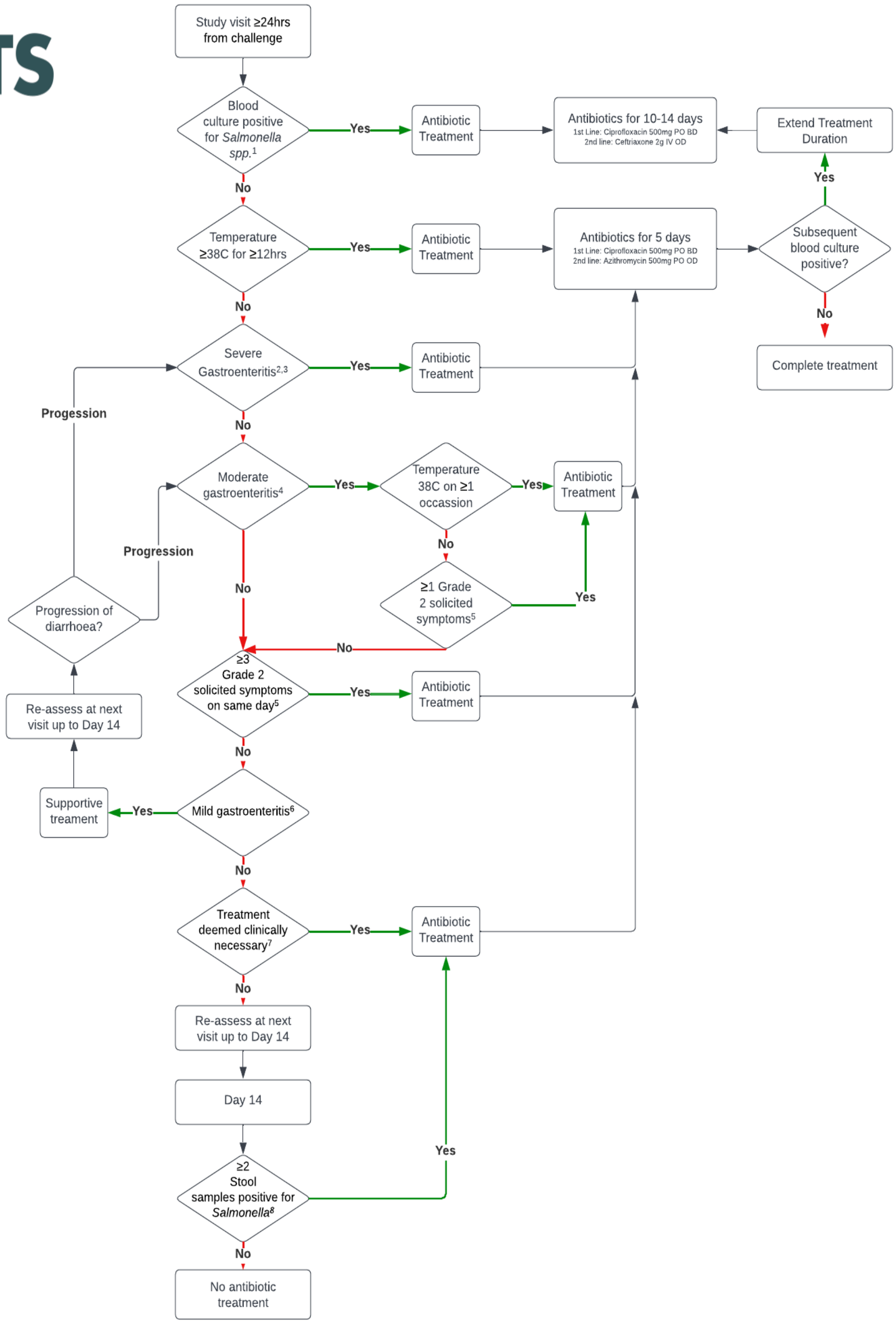

Notes

1 - Treatment initiated upon identification of Gram negative rods in blood culture, prior to formal speciation.

2- Loose/liquid stools defined as Type 6 or 7 stools on the Bristol stool chart.

3 - Severe gastroenteritis defined over a rolling 24-hour period as:
- ≥6 loose/liquid stools **and/or**
- >800 g of loose/liquid stools **and/or**
- ≥2 stools with gross blood

4 - Moderate Gastroenteritis defined over a rolling 24-hour period as:
- 4-5 loose/liquid stools **and/or**
- 400-800g loose/liquid stools

5 - Solicited symptoms defined as:
- Headache
- Fatigue/Malaise
- Anorexia
- Abdominal pain
- Nausea
- Vomiting
- Myalgia
- Arthralgia
- Cough

6 - Mild Gastroenteritis defined over a rolling 24-hour period as:
- 3 loose/liquid stools **and/or**
- 200-399g loose/liquid stools

7 - At the discretion of the principal investigator in consultation with the trial steering committee

8 - As detected by culture and/or PCR

Figure 2: Quarantine Discharge Algorithm

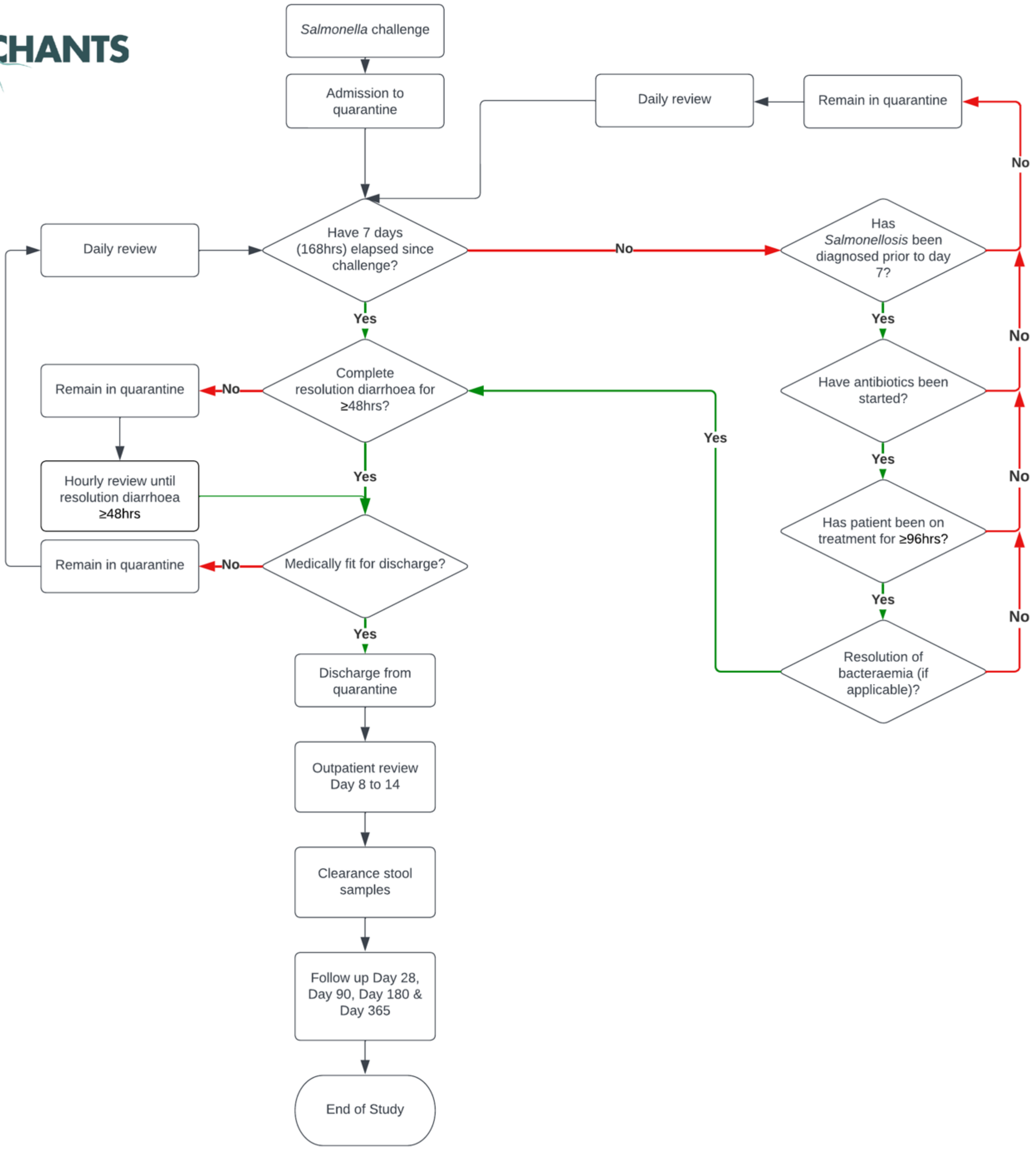

SPIRIT

STANDARD PROTOCOL ITEMS: RECOMMENDATIONS FOR INTERVENTIONAL TRIALS

SPIRIT 2013 Checklist: Recommended items to address in a clinical trial protocol and related documents*

Section/item	Item No.	Description	Addressed on Page
**Administrative information**			
Title	1	Descriptive title identifying the study design, population, interventions, and, if applicable, trial acronym	1
Trial registration	2a	Trial identifier and registry name. If not yet registered, name of intended registry	2
	2b	All items from the World Health Organization Trial Registration Data Set	2
Protocol version	3	Date and version identifier	1
Funding	4	Sources and types of financial, material, and other support	17
Roles and responsibilities	5a	Names, affiliations, and roles of protocol contributors	1
	5b	Name and contact information for the trial sponsor	1
	5c	Role of study sponsor and funders, if any, in study design; collection, management, analysis, and interpretation of data; writing of the report; and the decision to submit the report for publication, including whether they will have ultimate authority over any of these activities	17

For peer review only

	5d	Composition, roles, and responsibilities of the coordinating centre, steering committee, endpoint adjudication committee, data management team, and other individuals or groups overseeing the trial, if applicable (see Item 21a for data monitoring committee)	8, 11
**Introduction**			
Background and rationale	6a	Description of research question and justification for undertaking the trial, including summary of relevant studies (published and unpublished) examining benefits and harms for each intervention	4-5
	6b	Explanation for choice of comparators	5, 7
Objectives	7	Specific objectives or hypotheses	6, 13
Trial design	8	Description of trial design including type of trial (eg, parallel group, crossover, factorial, single group), allocation ratio, and framework (eg, superiority, equivalence, noninferiority, exploratory)	5-9
**Methods: Participants, interventions, and outcomes**			
Study setting	9	Description of study settings (eg, community clinic, academic hospital) and list of countries where data will be collected. Reference to where list of study sites can be obtained	5

For peer review only

Eligibility criteria	10	Inclusion and exclusion criteria for participants. If applicable, eligibility criteria for study centres and individuals who will perform the interventions (eg, surgeons, psychotherapists)	6-7 (and supplementary table 1)
Interventions	11a	Interventions for each group with sufficient detail to allow replication, including how and when they will be administered	8-9
	11b	Criteria for discontinuing or modifying allocated interventions for a given trial participant (eg, drug dose change in response to harms, participant request, or improving/worsening disease)	N/A
	11c	Strategies to improve adherence to intervention protocols, and any procedures for monitoring adherence (eg, drug tablet return, laboratory tests)	N/A
	11d	Relevant concomitant care and interventions that are permitted or prohibited during the trial	9-10
Outcomes	12	Primary, secondary, and other outcomes, including the specific measurement variable (eg, systolic blood pressure), analysis metric (eg, change from baseline, final value, time to event), method of aggregation (eg, median, proportion), and time point for each outcome. Explanation of the clinical relevance of chosen efficacy and harm outcomes is strongly recommended	6 (and table 1)
Participant timeline	13	Time schedule of enrolment, interventions (including any run-ins and washouts), assessments, and visits for participants. A schematic diagram is highly recommended (see Figure)	Figure 1
Sample size	14	Estimated number of participants needed to achieve study objectives and how it was determined, including clinical and statistical assumptions supporting any sample size calculations	11-12
Recruitment	15	Strategies for achieving adequate participant enrolment to reach target sample size	6-7

Methods: Assignment of interventions (for controlled trials)

Allocation:

Sequence generation	16a	Method of generating the allocation sequence (eg, computer-generated random numbers), and list of any factors for stratification. To reduce predictability of a random sequence, details of any planned restriction (eg, blocking) should be provided in a separate document that is unavailable to those who enrol participants or assign interventions	8
Allocation concealment mechanism	16b	Mechanism of implementing the allocation sequence (eg, central telephone; sequentially numbered, opaque, sealed envelopes), describing any steps to conceal the sequence until interventions are assigned	8
Implementation	16c	Who will generate the allocation sequence, who will enrol participants, and who will assign participants to interventions	8
Blinding (masking)	17a	Who will be blinded after assignment to interventions (eg, trial participants, care providers, outcome assessors, data analysts), and how	8
	17b	If blinded, circumstances under which unblinding is permissible, and procedure for revealing a participant's allocated intervention during the trial	8

Methods: Data collection, management, and analysis

Data collection methods	18a	Plans for assessment and collection of outcome, baseline, and other trial data, including any related processes to promote data quality (eg, duplicate measurements, training of assessors) and a description of study instruments (eg, questionnaires, laboratory tests) along with their reliability and validity, if known. Reference to where data collection forms can be found, if not in the protocol	Table 1, Figure 1

For peer review only - http://bmjopen.bmj.com/site/about/guidelines.xhtml

	18b	Plans to promote participant retention and complete follow-up, including list of any outcome data to be collected for participants who discontinue or deviate from intervention protocols	Figure 1
Data management	19	Plans for data entry, coding, security, and storage, including any related processes to promote data quality (eg, double data entry; range checks for data values). Reference to where details of data management procedures can be found, if not in the protocol	11
Statistical methods	20a	Statistical methods for analysing primary and secondary outcomes. Reference to where other details of the statistical analysis plan can be found, if not in the protocol	11-12, Figure 2
	20b	Methods for any additional analyses (eg, subgroup and adjusted analyses)	Table 1
	20c	Definition of analysis population relating to protocol non-adherence (eg, as randomised analysis), and any statistical methods to handle missing data (eg, multiple imputation)	N/A
**Methods: Monitoring**			
Data monitoring	21a	Composition of data monitoring committee (DMC); summary of its role and reporting structure; statement of whether it is independent from the sponsor and competing interests; and reference to where further details about its charter can be found, if not in the protocol. Alternatively, an explanation of why a DMC is not needed	11
	21b	Description of any interim analyses and stopping guidelines, including who will have access to these interim results and make the final decision to terminate the trial	11

Harms	22	Plans for collecting, assessing, reporting, and managing solicited and spontaneously reported adverse events and other unintended effects of trial interventions or trial conduct	11
Auditing	23	Frequency and procedures for auditing trial conduct, if any, and whether the process will be independent from investigators and the sponsor	8, 11
**Ethics and dissemination**			
Research ethics approval	24	Plans for seeking research ethics committee/institutional review board (REC/IRB) approval	12
Protocol amendments	25	Plans for communicating important protocol modifications (eg, changes to eligibility criteria, outcomes, analyses) to relevant parties (eg, investigators, REC/IRBs, trial participants, trial registries, journals, regulators)	12
Consent or assent	26a	Who will obtain informed consent or assent from potential trial participants or authorised surrogates, and how (see Item 32)	6-7
	26b	Additional consent provisions for collection and use of participant data and biological specimens in ancillary studies, if applicable	N/A
Confidentiality	27	How personal information about potential and enrolled participants will be collected, shared, and maintained in order to protect confidentiality before, during, and after the trial	11
Declaration of interests	28	Financial and other competing interests for principal investigators for the overall trial and each study site	17
Access to data	29	Statement of who will have access to the final trial dataset, and disclosure of contractual agreements that limit such access for investigators	N/A

For peer review only

Ancillary and post-trial care	30	Provisions, if any, for ancillary and post-trial care, and for compensation to those who suffer harm from trial participation	12
Dissemination policy	31a	Plans for investigators and sponsor to communicate trial results to participants, healthcare professionals, the public, and other relevant groups (eg, via publication, reporting in results databases, or other data sharing arrangements), including any publication restrictions	12
	31b	Authorship eligibility guidelines and any intended use of professional writers	17
	31c	Plans, if any, for granting public access to the full protocol, participant-level dataset, and statistical code	N/A
**Appendices**			
Informed consent materials	32	Model consent form and other related documentation given to participants and authorised surrogates	Supplementary
Biological specimens	33	Plans for collection, laboratory evaluation, and storage of biological specimens for genetic or molecular analysis in the current trial and for future use in ancillary studies, if applicable	Figure 1, Table 1

*It is strongly recommended that this checklist be read in conjunction with the SPIRIT 2013 Explanation & Elaboration for important clarification on the items. Amendments to the protocol should be tracked and dated. The SPIRIT checklist is copyrighted by the SPIRIT Group under the Creative Commons “Attribution-NonCommercial-NoDerivs 3.0 Unported” license.

For peer review only
